# Supplementary material for: Consultation patterns and frequent attenders in UK primary care from 2000 to 2019: a retrospective cohort analysis of consultation events across 845 general practices
Source: BMJ Open. 2021 Dec 20;11(12):e054666. doi: 10.1136/bmjopen-2021-054666 (PMC8718478; doi:10.1136/bmjopen-2021-054666)
Supplement: Supplementary data [file bmjopen-2021-054666supp001.pdf]

Supplementary file to "Consultation patterns and frequent attenders in UK primary care from 2000 to 2019: a retrospective cohort analysis of consultation events across 845 general practices"

Defining the consultation groups

GPs defined as: 1,2,3,4,5,6,7,8,9,10,47,50 in Table 1 (in red font).

Table 1: Staff Role

Code	Description
0	Data Not Entered
1	Senior Partner
2	Partner
3	Assistant
4	Associate
5	Non-commercial local rota of less than 10 GPs
6	Commercial Deputising service
7	Locum
8	GP Registrar
9	Consultant
10	Sole Practitioner
11	Practice Nurse
12	Health Visitor
13	Community Nurse
14	Midwife
15	Community Psychiatric Nurse
16	Social Worker
17	Pharmacist
18	Dispenser
19	Non-qualified Dispenser
20	Practice Manager
21	Fund Manager
22	Business Manager
23	Administrator
24	Secretary
25	Receptionist
26	Physiotherapist
27	Chiropodist
28	Dentist
29	Dietician
30	Counsellor
31	Osteopath
32	Maintenance staff
33	Other Health Care Professional
34	Hospital Nurse
35	Community Medical Officer
36	School Nurse

Kontopantelis E, et al. BMJ Open 2021; 11:e054666. doi: 10.1136/bmjopen-2021-054666

Code	Description
37	Health Education Officer
38	Contact Tracing Nurse
39	Stomatherapist
40	Computer Manager
41	Interpreter/Link Worker
42	Chiropractor
43	Acupuncturist
44	Homeopath
45	Mental Handicap Nurse
46	Carer
47	Salaried Partner
48	Occupational Therapist
49	Speech Therapist
50	GP Retainer
51	Phlebotomist
52	Other Medical & Dental
53	Other Students
54	Other Nursing & Midwifery
55	Other Allied Health Professionals
56	Other Professional Scientific & Technical
57	Other Healthcare Scientists
58	Other Additional Clinical Services
59	Other Admin & Clerical
60	Clinical Practitioner Access Role
61	Nurse Access Role
62	Nurse Manager Access Role
63	Health Professional Access Role
64	Healthcare Student Access Role
65	Biomedical Scientist Access Role
66	Clinical Coder Access Role
67	Optometrist
68	Radiographer

Face-to-face consultations defined as 1,2,3,4,6,7,8,9,11,18,24,27,28,30,31,32,33,34,36,37,38,50,61 in Table 2 (in red font).

Table 2: Consultation type

Code	Description
0	Data Not Entered
1	Clinic
2	Night visit, Deputising service
3	Follow-up/routine visit
4	Night visit, Local rota
5	Mail from patient
6	Night visit , practice
7	Out of hours, Practice
8	Out of hours, Non Practice

Kontopantelis E, *et al. BMJ Open* 2021; 11:e054666. doi: 10.1136/bmjopen-2021-054666

Code	Description
9	Surgery consultation
10	Telephone call from a patient
11	Acute visit
12	Discharge details
13	Letter from Outpatients
14	Repeat Issue
15	Other
16	Results recording
17	Mail to patient
18	Emergency Consultation
19	Administration
20	Casualty Attendance
21	Telephone call to a patient
22	Third Party Consultation
23	Hospital Admission
24	Children's Home Visit
25	Day Case Report
26	GOS18 Report
27	Home Visit
28	Hotel Visit
29	NHS Direct Report
30	Nursing Home Visit
31	Residential Home Visit
32	Twilight Visit
33	Triage
34	Walk-in Centre
35	Co-op Telephone advice
36	Co-op Surgery Consultation
37	Co-op Home Visit
38	Minor Injury Service
39	Medicine Management
40	Community Clinic
41	Community Nursing Note
42	Community Nursing Report
43	Data Transferred from other system
44	Health Authority Entry
45	Health Visitor Note
46	Health Visitor Report
47	Hospital Inpatient Report
48	Initial Post Discharge Review
49	Laboratory Request
50	Night Visit
51	Radiology Request
52	Radiology Result
53	Referral Letter
54	Social Services Report
55	Telephone Consultation

Kontopantelis E, *et al. BMJ Open* 2021; 11:e054666. doi: 10.1136/bmjopen-2021-054666

**Code**	**Description**
56	Template Entry
57	GP to GP communication transaction
58	Non-consultation medication data
59	Non-consultation data
60	ePharmacy message
61	Extended Hours

Kontopantelis E, *et al. BMJ Open* 2021; 11:e054666. doi: 10.1136/bmjopen-2021-054666

Median (50th centile)

Table 3 Medians (25th – 75th centiles) of median number of consultations by consultation type, all practices

Year	All consultations by all staff	All consultations by GPs	Face-to-face consultations by all staff	Face-to-face consultations by GPs
**2000-2001**	7 (6-8)	3 (3-4)	4 (3-4)	2 (2-3)
**2001-2002**	8 (7-9)	3 (3-4)	4 (3-4)	2 (2-3)
**2002-2003**	9 (7-10)	3 (3-4)	3 (3-4)	2 (2-3)
**2003-2004**	9 (7-11)	3 (3-4)	3 (3-4)	2 (2-3)
**2004-2005**	9 (7-11)	3 (3-4)	3 (3-4)	2 (2-3)
**2005-2006**	10 (8-13)	4 (3-4)	4 (3-4)	2 (2-3)
**2006-2007**	10 (8-13)	4 (3-4)	3 (3-4)	2 (2-3)
**2007-2008**	11 (9-13)	4 (3-5)	3 (3-4)	2 (2-3)
**2008-2009**	12 (10-14)	4 (3-5)	3 (3-4)	2 (2-3)
**2009-2010**	13 (11-15)	4 (3-5)	4 (3-4)	2 (2-3)
**2010-2011**	13 (11-15)	4 (3-5)	3 (3-4)	2 (2-3)
**2011-2012**	13 (11-15)	4 (4-5)	3 (3-4)	2 (2-3)
**2012-2013**	14 (12-16)	4 (4-5)	4 (3-4)	2 (2-3)
**2013-2014**	14 (12-17)	5 (4-5)	4 (3-4)	2 (2-3)
**2014-2015**	14 (12-17)	5 (4-6)	3 (3-4)	2 (2-3)
**2015-2016**	14 (12-17)	4 (3-5)	3 (3-4)	2 (1-2)
**2016-2017**	15 (12-17)	5 (4-6)	3 (3-4)	2 (1-2)
**2017-2018**	15 (13-18)	5 (4-6)	3 (3-4)	2 (1-2)
**2018-2019**	16 (13-18)	5 (4-6)	3 (2-4)	2 (1-2)

Kontopantelis E, *et al. BMJ Open* 2021; 11:e054666. doi: 10.1136/bmjopen-2021-054666

Table 4 Medians (25th – 75th centiles) of proportion of high volume (≥ 50th centile) by consultation type, all practices

Year	All consultations by all staff	All consultations by GPs	Face-to-face consultations by all staff	Face-to-face consultations by GPs
**2000-2001**	87.0 (86.2-87.8)	90.2 (88.2-92.4)	87.9 (86.0-90.1)	91.6 (87.9-94.0)
**2001-2002**	87.3 (86.4-88.1)	90.3 (88.4-92.5)	88.4 (86.8-90.4)	92.2 (88.7-94.0)
**2002-2003**	87.4 (86.6-88.2)	90.8 (89.1-93.0)	88.8 (87.2-90.5)	92.7 (89.2-94.2)
**2003-2004**	87.6 (86.9-88.4)	90.9 (89.3-92.8)	89.1 (87.5-90.8)	92.7 (89.6-94.1)
**2004-2005**	88.3 (87.5-89.1)	91.8 (89.7-93.9)	90.2 (88.4-92.6)	93.3 (90.5-94.9)
**2005-2006**	88.0 (87.2-88.8)	91.5 (89.7-93.2)	89.5 (87.8-91.5)	92.9 (90.3-94.4)
**2006-2007**	88.4 (87.5-89.3)	92.1 (90.1-94.1)	90.7 (88.5-92.7)	93.4 (91.0-95.2)
**2007-2008**	88.1 (87.2-88.9)	91.5 (89.9-93.0)	89.7 (87.9-91.7)	93.0 (90.7-94.5)
**2008-2009**	88.1 (87.3-88.8)	91.5 (90.0-93.0)	90.0 (88.3-91.9)	93.4 (91.2-94.8)
**2009-2010**	87.9 (87.2-88.7)	91.3 (90.0-93.0)	89.8 (88.2-91.9)	93.2 (90.8-94.7)
**2010-2011**	88.0 (87.3-88.8)	91.4 (90.0-93.0)	90.0 (88.3-92.0)	93.3 (91.2-94.8)
**2011-2012**	88.1 (87.4-88.8)	91.4 (90.1-92.6)	90.2 (88.6-92.0)	93.3 (91.4-95.0)
**2012-2013**	88.1 (87.4-88.9)	91.4 (90.1-92.6)	90.0 (88.5-92.0)	93.4 (91.1-94.9)
**2013-2014**	88.1 (87.3-88.8)	91.5 (90.4-92.7)	90.3 (88.8-92.0)	93.5 (91.7-95.2)
**2014-2015**	88.3 (87.4-88.9)	91.6 (90.4-93.0)	90.9 (89.3-92.5)	93.7 (91.8-95.3)
**2015-2016**	87.7 (86.8-88.7)	92.2 (90.7-94.2)	92.0 (90.1-94.1)	94.7 (92.9-100)
**2016-2017**	88.2 (87.4-88.9)	91.8 (90.6-93.3)	91.7 (89.8-93.4)	94.4 (92.3-96.5)
**2017-2018**	88.2 (87.3-88.9)	91.7 (90.4-93.1)	91.6 (89.7-93.4)	94.4 (92.4-100)
**2018-2019**	88.2 (87.3-88.9)	91.9 (90.6-92.9)	91.8 (89.9-93.9)	94.5 (92.6-100)

Kontopantelis E, *et al. BMJ Open* 2021; 11:e054666. doi: 10.1136/bmjopen-2021-054666

Table 5: Medians (25th – 75th centiles) of ratio for users ≥50th centile over the rest of consultations by consultation type, all practices

Year	All consultations by all staff	All consultations by GPs	Face-to-face consultations by all staff	Face-to-face consultations by GPs
2000-2001	6.1 (5.7-6.4)	7.5 (6.5-8.9)	6.0 (5.5-6.7)	7.7 (6.3-9.5)
2001-2002	6.3 (5.9-6.7)	7.7 (6.6-9.1)	6.4 (5.8-7.1)	8.2 (6.6-9.6)
2002-2003	6.5 (6.0-6.9)	8.0 (6.9-9.7)	6.5 (6.0-7.4)	8.5 (6.8-9.8)
2003-2004	6.6 (6.2-7.1)	8.4 (7.3-10.1)	6.7 (6.2-7.6)	8.7 (7.2-10.1)
2004-2005	7.0 (6.6-7.6)	9.2 (7.6-12.0)	7.5 (6.6-9.7)	9.4 (7.7-11.4)
2005-2006	6.9 (6.5-7.4)	9.0 (7.8-10.7)	7.1 (6.4-8.2)	9.2 (7.6-10.6)
2006-2007	7.2 (6.6-7.8)	9.9 (8.0-12.6)	7.8 (6.8-10)	9.9 (8.0-12.1)
2007-2008	7.0 (6.6-7.5)	9.2 (7.9-10.8)	7.2 (6.5-8.5)	9.2 (7.7-10.7)
2008-2009	7.0 (6.6-7.5)	9.2 (8.1-11.0)	7.4 (6.6-8.6)	9.7 (8.2-11.4)
2009-2010	6.9 (6.5-7.4)	9.3 (8.0-11.0)	7.4 (6.6-8.6)	9.8 (8.0-11.2)
2010-2011	7.0 (6.6-7.5)	9.3 (8.0-11.0)	7.5 (6.6-8.6)	9.9 (8.3-11.4)
2011-2012	7.1 (6.6-7.6)	9.3 (8.1-10.7)	7.5 (6.8-8.7)	10.0 (8.4-11.4)
2012-2013	7.1 (6.7-7.6)	9.3 (8.2-10.6)	7.6 (6.8-8.8)	9.8 (8.3-11.4)
2013-2014	7.1 (6.6-7.6)	9.6 (8.5-11.0)	7.7 (7.0-8.9)	10.2 (8.7-11.9)
2014-2015	7.2 (6.7-7.7)	9.7 (8.6-11.3)	8.2 (7.3-9.5)	10.5 (8.9-12.1)
2015-2016	6.9 (6.4-7.5)	10.4 (8.6-14.4)	9.5 (7.7-11.9)	11.4 (9.6-14.2)
2016-2017	7.2 (6.6-7.7)	9.8 (8.7-12.1)	8.7 (7.6-10.5)	11.0 (9.3-13.0)
2017-2018	7.1 (6.6-7.7)	9.9 (8.7-11.8)	8.6 (7.6-10.8)	11.1 (9.2-12.9)
2018-2019	7.2 (6.6-7.8)	9.9 (8.8-11.8)	8.9 (7.8-11.2)	11.2 (9.6-13.3)

Kontopantelis E, *et al. BMJ Open* 2021; 11:e054666. doi: 10.1136/bmjopen-2021-054666

75th centile

Table 6 Medians (25th – 75th centiles) of 75th centile number of consultations by consultation type, all practices

Year	All consultations by all staff	All consultations by GPs	Face-to-face consultations by all staff	Face-to-face consultations by GPs
**2000-2001**	16 (14-18)	7 (6-10)	7 (6-9)	5 (4-6)
**2001-2002**	17.5 (15-20)	7 (6-9)	7 (6-9)	5 (4-6)
**2002-2003**	19 (16-22)	7 (6-9)	7 (6-9)	5 (4-6)
**2003-2004**	21 (17-24)	8 (6-10)	7 (6-9)	5 (4-6)
**2004-2005**	22 (18-26)	8 (7-10)	7 (6-9)	5 (4-6)
**2005-2006**	24 (19-28)	9 (7-11)	7 (6-10)	5 (4-6)
**2006-2007**	24 (20-29)	9 (7-11)	7 (6-9)	4 (4-6)
**2007-2008**	26 (21-30)	9 (7-11)	7 (6-9)	5 (4-6)
**2008-2009**	27 (23-31.5)	9 (8-11)	7 (6-9)	5 (4-6)
**2009-2010**	28 (24-33)	10 (8-12)	7 (6-9)	5 (4-6)
**2010-2011**	28 (24-33)	10 (8-12)	7 (6-9)	5 (4-6)
**2011-2012**	29 (25-34)	10 (9-12)	7 (6-9)	5 (4-6)
**2012-2013**	30 (26-35)	10 (9-12)	7 (6-9)	5 (4-6)
**2013-2014**	32 (27-37)	11 (9-13)	7 (6-9)	5 (4-6)
**2014-2015**	32 (28-37)	11 (10-13)	7 (6-9)	5 (4-6)
**2015-2016**	32 (28-37)	11 (9-13)	7 (6-8)	5 (4-5)
**2016-2017**	32 (28-38)	11 (9-13)	7 (6-8)	5 (4-6)
**2017-2018**	33 (29-38)	11 (9-13)	7 (6-8)	4 (3-5)
**2018-2019**	35 (30-40)	11 (10-13)	7 (6-8)	4 (3-5)

Table 7 Medians (25th – 75th centiles) of proportion of high volume (≥ 75th centile) by consultation type, all practices

Year	All consultations by all staff	All consultations by GPs	Face-to-face consultations by all staff	Face-to-face consultations by GPs
**2000-2001**	62.4 (61.2-63.9)	65.8 (63.5-69.1)	63.7 (61.6-66.0)	66.4 (63.8-69.9)
**2001-2002**	62.9 (61.6-64.3)	66.5 (64.0-69.1)	63.7 (61.8-66.4)	66.8 (64.3-70.4)
**2002-2003**	63.1 (61.9-64.6)	67.4 (64.7-69.8)	64.4 (62.3-66.7)	67.9 (64.3-71.3)
**2003-2004**	63.5 (62.2-64.8)	67.7 (65.4-70.2)	64.9 (62.7-67.3)	68.2 (65.0-71.8)
**2004-2005**	64.3 (63.0-65.7)	68.9 (66.1-71.7)	66.2 (63.9-69.0)	69.5 (66.1-73.4)
**2005-2006**	63.8 (62.6-65.3)	68.2 (65.9-70.8)	65.4 (63.2-67.7)	68.9 (65.7-72.2)
**2006-2007**	64.6 (63.0-66.0)	69.3 (66.3-71.9)	66.4 (64.0-69.4)	70.3 (66.7-73.9)
**2007-2008**	64.1 (62.7-65.4)	68.4 (66.4-70.7)	65.5 (63.2-68.0)	69.2 (66.0-72.8)
**2008-2009**	64.2 (62.7-65.5)	68.2 (66.5-70.7)	65.8 (63.9-68.6)	69.5 (66.4-72.7)
**2009-2010**	63.8 (62.6-65.2)	68.4 (66.2-70.5)	65.8 (63.7-68.0)	69.5 (66.6-72.4)
**2010-2011**	64.0 (62.7-65.5)	68.3 (66.4-70.4)	66.3 (64.2-68.5)	69.5 (66.8-72.4)
**2011-2012**	64.3 (62.9-65.4)	68.4 (66.5-70.2)	66.2 (64.1-68.8)	69.8 (67.0-73.0)
**2012-2013**	64.3 (62.9-65.6)	68.4 (66.8-70.2)	66.5 (64.6-68.8)	69.6 (67.0-73.1)
**2013-2014**	64.2 (62.9-65.7)	68.7 (66.8-70.5)	66.7 (64.6-69.0)	70.1 (67.4-73.4)
**2014-2015**	64.5 (62.9-65.9)	69.0 (67.3-70.8)	67.3 (65.3-69.4)	70.6 (67.9-73.8)
**2015-2016**	63.7 (62.4-65.5)	69.6 (67.4-72.5)	68.8 (66.4-71.7)	72.3 (69.1-76.8)
**2016-2017**	64.3 (62.8-65.8)	69.2 (67.3-71.2)	68.2 (66.0-70.7)	71.5 (68.3-75.6)
**2017-2018**	64.1 (62.7-65.8)	69.0 (67.1-71.3)	68.4 (65.5-71.4)	72.1 (69.0-76.1)
**2018-2019**	64.2 (62.6-65.5)	69.0 (67.3-71.3)	68.6 (66.2-71.2)	72.6 (69.3-77.2)

Table 8: Medians (25th – 75th centiles) of ratio for users ≥75th centile over the rest of consultations by consultation type, all practices

Year	All consultations by all staff	All consultations by GPs	Face-to-face consultations by all staff	Face-to-face consultations by GPs
2000-2001	4.7 (4.5-5.0)	5.1 (4.8-5.7)	4.6 (4.4-4.9)	5.0 (4.6-5.5)
2001-2002	4.8 (4.6-5.1)	5.3 (4.9-5.8)	4.7 (4.4-5.0)	5.1 (4.7-5.7)
2002-2003	4.9 (4.6-5.2)	5.4 (5.0-6.1)	4.8 (4.5-5.2)	5.1 (4.8-5.8)
2003-2004	5.0 (4.7-5.3)	5.7 (5.2-6.3)	4.9 (4.6-5.3)	5.3 (4.9-6.0)
2004-2005	5.2 (4.9-5.5)	6.0 (5.4-6.7)	5.2 (4.8-5.8)	5.7 (5.2-6.6)
2005-2006	5.1 (4.8-5.4)	5.8 (5.4-6.5)	5.0 (4.7-5.5)	5.5 (5.0-6.3)
2006-2007	5.3 (4.9-5.6)	6.2 (5.5-7.0)	5.3 (4.9-6.0)	5.9 (5.2-6.8)
2007-2008	5.2 (4.9-5.5)	5.9 (5.5-6.5)	5.1 (4.7-5.5)	5.6 (5.1-6.2)
2008-2009	5.2 (4.9-5.5)	5.9 (5.5-6.6)	5.1 (4.8-5.6)	5.6 (5.2-6.3)
2009-2010	5.1 (4.9-5.4)	6.0 (5.5-6.5)	5.1 (4.8-5.6)	5.6 (5.2-6.3)
2010-2011	5.2 (4.9-5.5)	6.0 (5.5-6.5)	5.2 (4.8-5.6)	5.6 (5.2-6.2)
2011-2012	5.2 (4.9-5.5)	6.0 (5.6-6.5)	5.2 (4.9-5.7)	5.7 (5.3-6.3)
2012-2013	5.2 (4.9-5.6)	6.0 (5.6-6.5)	5.3 (4.9-5.7)	5.8 (5.3-6.4)
2013-2014	5.2 (5.0-5.6)	6.1 (5.7-6.6)	5.3 (5.0-5.8)	5.9 (5.4-6.5)
2014-2015	5.3 (5.0-5.6)	6.2 (5.8-6.7)	5.4 (5.1-6.0)	6.0 (5.5-6.7)
2015-2016	5.2 (4.8-5.6)	6.4 (5.9-7.3)	5.9 (5.3-6.6)	6.6 (5.8-7.7)
2016-2017	5.3 (4.9-5.6)	6.3 (5.8-6.9)	5.7 (5.2-6.3)	6.3 (5.7-7.3)
2017-2018	5.2 (4.9-5.6)	6.3 (5.7-6.8)	5.7 (5.2-6.3)	6.4 (5.8-7.4)
2018-2019	5.2 (4.9-5.6)	6.3 (5.8-6.9)	5.7 (5.3-6.4)	6.5 (5.8-7.7)

Kontopantelis E, *et al. BMJ Open* 2021; 11:e054666. doi: 10.1136/bmjopen-2021-054666

Twelve or more consultations

Table 9 Medians (25th – 75th centiles) of proportion of patients with 12 or more consultations by consultation type, all practices

Year	All consultations by all staff	All consultations by GPs	Face-to-face consultations by all staff	Face-to-face consultations by GPs
**2000-2001**	36.0 (30.5-40.5)	12.6 (7.5-20.1)	10.6 (7.9-15.6)	4.9 (3.4-9.3)
**2001-2002**	38.9 (32.9-42.8)	12.5 (8.1-17.9)	11.2 (7.9-16.7)	4.6 (3.0-8.4)
**2002-2003**	40.9 (34.7-45.9)	12.7 (7.7-18.2)	10.7 (7.5-16.4)	4.3 (2.8-7.7)
**2003-2004**	43.3 (37.2-48.4)	14.4 (10.2-19.5)	10.8 (7.5-18.1)	4.2 (2.6-7.5)
**2004-2005**	44.3 (37.8-49.5)	15.7 (11.6-20.7)	11.2 (7.4-18.4)	4.1 (2.5-7.9)
**2005-2006**	47.2 (40.7-52.5)	17.0 (13.3-22.7)	11.9 (8.1-20.1)	4.3 (2.7-8.0)
**2006-2007**	47.3 (41.0-52.4)	17.5 (13.2-22.8)	11.6 (7.7-19.5)	4.2 (2.6-8.2)
**2007-2008**	49.5 (44.2-53.9)	18.0 (13.0-23.3)	11.8 (8.2-17.9)	4.2 (2.8-7.2)
**2008-2009**	51.1 (46.2-55.2)	19.0 (14.3-23.7)	11.7 (8.4-16.9)	4.4 (2.9-6.6)
**2009-2010**	52.8 (48.2-56.6)	20.0 (15.4-25.4)	12.2 (9.0-17.0)	4.7 (2.9-6.8)
**2010-2011**	52.7 (48.3-56.7)	20.7 (16.4-25.3)	12.1 (8.6-16.3)	4.7 (2.9-7.0)
**2011-2012**	53.8 (49.4-57.5)	21.8 (17.3-26.2)	12.2 (8.7-16.1)	4.7 (3.0-7.0)
**2012-2013**	54.5 (50.4-58.4)	22.3 (18.1-27.4)	12.4 (9.1-16.4)	4.6 (3.0-7.4)
**2013-2014**	55.6 (51.2-59.7)	23.8 (19.4-28.6)	12.5 (8.9-16.4)	4.6 (2.9-7.4)
**2014-2015**	56.0 (51.8-60.1)	24.1 (19.8-29.2)	12.3 (8.8-16.3)	4.6 (2.9-7.3)
**2015-2016**	56.2 (52.1-60.5)	23.0 (18.7-28.0)	11.7 (8.3-15.5)	4.5 (2.4-7.2)
**2016-2017**	56.4 (52.1-60.4)	24.0 (18.9-29.2)	11.8 (8.2-16.1)	4.5 (2.5-7.7)
**2017-2018**	56.9 (52.9-60.9)	24.0 (19.6-28.9)	11.4 (7.4-15.8)	4.2 (2.2-7.3)
**2018-2019**	58.4 (54.1-61.8)	24.7 (19.9-29.2)	11.3 (7.3-15.4)	4.3 (2.1-7.1)

Kontopantelis E, *et al. BMJ Open* 2021; 11:e054666. doi: 10.1136/bmjopen-2021-054666

Table 10 Medians (25th – 75th centiles) of proportion of high volume (12 or more consultations) by consultation type, all practices

Year	All consultations by all staff	All consultations by GPs	Face-to-face consultations by all staff	Face-to-face consultations by GPs
**2000-2001**	73.9 (68.3-78.5)	43.1 (30.6-56.4)	35.8 (28.1-47.0)	20.7 (15.7-34.1)
**2001-2002**	77.6 (71.5-81.3)	43.2 (31.7-53.7)	36.8 (28.6-48.1)	20.2 (15.1-31.7)
**2002-2003**	79.8 (74.1-83.8)	43.6 (31.1-54.8)	36.1 (28.3-49.3)	19.4 (14.0-29.8)
**2003-2004**	82.3 (76.5-85.9)	48.3 (37.5-57.2)	37.3 (28.4-52.7)	19.4 (13.8-31.0)
**2004-2005**	83.8 (78.2-87.0)	51.7 (42.4-60.2)	39.3 (29.7-55.8)	19.7 (13.6-33.3)
**2005-2006**	85.6 (80.5-88.8)	54.1 (45.7-62.5)	40.4 (30.7-57.0)	20.1 (14.4-33.4)
**2006-2007**	86.1 (81.5-89.1)	55.9 (46.9-64.2)	41.1 (31.8-56.0)	20.4 (14.8-33.2)
**2007-2008**	87.2 (83.3-89.8)	55.7 (46.2-64.0)	40.2 (31.4-52.6)	20.4 (14.8-30.6)
**2008-2009**	88.3 (84.9-90.6)	57.5 (48.5-64.5)	40.1 (32.0-51.0)	21.2 (15.1-28.9)
**2009-2010**	88.9 (86.2-91.1)	59.3 (50.6-66.6)	41.0 (33.2-51.0)	22.0 (15.5-29.5)
**2010-2011**	89.1 (86.4-91.3)	59.9 (52.3-66.7)	41.0 (32.7-50.1)	21.9 (15.7-30.1)
**2011-2012**	89.8 (87.3-91.7)	61.6 (54.1-68.4)	41.7 (33.0-49.5)	21.9 (16.0-30.0)
**2012-2013**	90.2 (87.8-92.1)	62.8 (56.1-70.3)	42.4 (33.4-50.0)	22.3 (16.1-31.3)
**2013-2014**	90.7 (88.3-92.6)	65.1 (58.4-71.4)	42.5 (33.8-50.5)	22.1 (15.8-32.1)
**2014-2015**	90.9 (88.7-92.8)	66.2 (59.6-72.0)	43.2 (34.0-50.5)	22.9 (16.1-32.4)
**2015-2016**	90.6 (88.3-92.5)	65.7 (58.4-71.6)	43.0 (33.7-50.8)	23.1 (15.2-32.4)
**2016-2017**	91.2 (88.9-93.0)	66.1 (58.7-72.7)	42.9 (33.5-51.5)	22.7 (14.5-34.0)
**2017-2018**	91.3 (89.3-93.2)	65.8 (59.2-72.2)	41.7 (32.0-51.5)	22.9 (14.0-32.5)
**2018-2019**	92.0 (90.2-93.6)	67.0 (59.9-73.0)	41.6 (31.5-50.5)	22.2 (13.4-32.0)

Kontopantelis E, *et al. BMJ Open* 2021; 11:e054666. doi: 10.1136/bmjopen-2021-054666
