# Supplementary material for: Consultation patterns and frequent attenders in UK primary care from 2000 to 2019: a retrospective cohort analysis of consultation events across 845 general practices
Source: BMJ Open. 2021 Dec 20;11(12):e054666. doi: 10.1136/bmjopen-2021-054666 (PMC8718478; doi:10.1136/bmjopen-2021-054666)
Supplement: Author's manuscript [file bmjopen-2021-054666.draft_revisions.pdf]

BMJ Open

BMJ Open is committed to open peer review. As part of this commitment we make the peer review history of every article we publish publicly available.

When an article is published we post the peer reviewers' comments and the authors' responses online. We also post the versions of the paper that were used during peer review. These are the versions that the peer review comments apply to.

The versions of the paper that follow are the versions that were submitted during the peer review process. They are not the versions of record or the final published versions. They should not be cited or distributed as the published version of this manuscript.

BMJ Open is an open access journal and the full, final, typeset and author-corrected version of record of the manuscript is available on our site with no access controls, subscription charges or pay-per-view fees (http://bmjopen.bmj.com).

If you have any questions on BMJ Open's open peer review process please email info.bmjopen@bmj.com

BMJ Open

Consultation patterns and frequent attenders in UK primary care from 2000 to 2019: a cohort analysis of 1,699,709,314 consultation events across 12,330,545 patients in 845 general practices.

Journal:	*BMJ Open*
Manuscript ID	bmjopen-2021-054666
Article Type:	Original research
Date Submitted by the Author:	21-Jun-2021
Complete List of Authors:	Kontopantelis, Evangelos; The University of Manchester, Division of Informatics, Imaging and Data Science; NIHR School for Primary Care Research, University of Oxford<br>Panagioti, Maria; The University of Manchester, Division of Population Health, Health Services Research & Primary Care; The University of Manchester, NIHR Greater Manchester Patient Safety Translational Research Centre<br>Farragher, Tracey; The University of Manchester, Division of Population Health, Health Services Research & Primary Care<br>Munford, Luke; The University of Manchester, Division of Population Health, Health Services Research & Primary Care<br>Parisi, Rosa; The University of Manchester, Division of Informatics, Imaging and Data Science<br>Planner, Claire; The University of Manchester, Division of Population Health, Health Services Research & Primary Care; NIHR School for Primary Care Research, University of Oxford<br>Spooner, Sharon; The University of Manchester, Health Organisation, Policy and Economics (HOPE) Group, Centre for Primary Care & Health Services Research; The University of Manchester, Division of Population Health, Health Services Research & Primary Care<br>Tse, Alice; The University of Manchester, Division of Population Health, Health Services Research & Primary Care<br>Ashcroft, Darren; The University of Manchester, NIHR Greater Manchester Patient Safety Translational Research Centre; The University of Manchester, Division of Pharmacy & Optometry<br>Esmail, Aneez; The University of Manchester, Division of Population Health, Health Services Research & Primary Care; NIHR School for Primary Care Research, University of Oxford
Keywords:	PRIMARY CARE, Health policy < HEALTH SERVICES ADMINISTRATION & MANAGEMENT, Organisation of health services < HEALTH SERVICES ADMINISTRATION & MANAGEMENT

For peer review only - http://bmjopen.bmj.com/site/about/guidelines.xhtml

For peer review only - http://bmjopen.bmj.com/site/about/guidelines.xhtml

I, the Submitting Author has the right to grant and does grant on behalf of all authors of the Work (as defined in the below author licence), an exclusive licence and/or a non-exclusive licence for contributions from authors who are: i) UK Crown employees; ii) where BMJ has agreed a CC-BY licence shall apply, and/or iii) in accordance with the terms applicable for US Federal Government officers or employees acting as part of their official duties; on a worldwide, perpetual, irrevocable, royalty-free basis to BMJ Publishing Group Ltd ("BMJ") its licensees and where the relevant Journal is co-owned by BMJ to the co-owners of the Journal, to publish the Work in this journal and any other BMJ products and to exploit all rights, as set out in our licence.

The Submitting Author accepts and understands that any supply made under these terms is made by BMJ to the Submitting Author unless you are acting as an employee on behalf of your employer or a postgraduate student of an affiliated institution which is paying any applicable article publishing charge ("APC") for Open Access articles. Where the Submitting Author wishes to make the Work available on an Open Access basis (and intends to pay the relevant APC), the terms of reuse of such Open Access shall be governed by a Creative Commons licence – details of these licences and which Creative Commons licence will apply to this Work are set out in our licence referred to above.

Other than as permitted in any relevant BMJ Author's Self Archiving Policies, I confirm this Work has not been accepted for publication elsewhere, is not being considered for publication elsewhere and does not duplicate material already published. I confirm all authors consent to publication of this Work and authorise the granting of this licence.

For peer review only

Consultation patterns and frequent attenders in UK primary care from 2000 to 2019: a cohort analysis of 1,699,709,314 consultation events across 12,330,545 patients in 845 general practices.

Evangelos Kontopantelis, Professor in Data Science and Health Services Research[1,2,3]
Maria Panagioti, Senior Lecturer[4,5]
Tracey Farragher, Lecturer in Healthcare Sciences[4]
Luke Munford, Lecturer in Health Economics[3,4]
Rosa Parisi, Research Fellow in Statistics[1,3]
Claire Planner, Research Associate[2,4]
Sharon Spooner, General Practitioner & Clinical Lecturer[2,3,4]
Alice Tse, General Practitioner[4]
Darren M. Ashcroft, Professor of Pharmacoepidemiology[2,5,6]
Aneez Esmail, Professor of General Practice[2,4]

1 Division of Informatics, Imaging and Data Science, University of Manchester, Manchester, England
2 NIHR School for Primary Care Research, University of Oxford, Oxford, England
3 Health Organisation, Policy and Economics (HOPE) research group; University of Manchester, Manchester, England
4 Division of Population Health, Health Services Research & Primary Care, University of Manchester, Manchester, England
5 NIHR Greater Manchester Patient Safety Translational Research Centre, Manchester, England
6 Division of Pharmacy & Optometry, University of Manchester, Manchester, England

Corresponding author:

Evangelos Kontopantelis, PhD
e.kontopantelis@manchester.ac.uk
Professor in Data Science and Health Services Research
Division of Informatics, Imaging and Data Sciences
University of Manchester
Oxford Road
M13 9PL
Manchester
UK

Keywords: primary care, consultations, workload, frequent attendance

For peer review only - http://bmjopen.bmj.com/site/about/guidelines.xhtml

Word count: 3,726

Abstract: 380

For peer review only

For peer review only - http://bmjopen.bmj.com/site/about/guidelines.xhtml

Abstract

Objective

To describe the distribution of consultations at the practice level and examine whether increases are uniform or driven by people who consult more frequently.

Design

Cohort study.

Setting

UK general practice data from the CPRD GOLD database.

Participants

1,699,709,314 consultation events from 12,330,545 patients, in 845 general practices, from 1 April 2000 to 31 March 2019.

Methods

Consultation information from the CPRD GOLD database was aggregated by financial year into four categories: all consultations/all staff; all consultations/GPs; face-to-face consultations/all staff; face-to-face consultations/GPs. We focused on patients with a number of consultations above the 90th centile, within each year and classified them as frequent attenders.

Results

Among frequent attenders, all consultations by GPs increased from a median (25th, 75th centile) of 13 (10 to 16) to 21 (18 to 25) and all consultations by all staff increased from 27 (23, 30) to 60 (51, 69) over the study period. Approximately 4/10 consultations of any type concerned frequent attenders and the proportion of consultations attributed to frequent attenders increased over time, particularly for face-to-face consultations with GPs, from a median of 38.0% (35.9%, 40.3%) in 2000/1 to 43.0% (40.6%, 46.4%) in 2018/9. Regression analyses indicated decreasing trends over time for face-to-face consultations and increasing trends for all consultation types, for both GPs and all staff. Adjusted incidence rate ratios for frequent attenders ranged between 4.992 (95%CI: 4.917, 5.068) for face-to-face consultations with all staff, and 5.603 (95%CI: 5.560, 5.647) for all consultations with GPs, compared to the rest of consulting practice population. Overall, most of the increases related to non face to face consultations, especially with other staff (not GPs) and with frequent attenders.

Conclusions

Frequent attenders largely and progressively contribute to increased workload in general practices across the UK over the last 20 years. Important knowledge gaps remain in terms of the demographic, social and health characteristics of frequent attenders in the UK and how general practices can be prepared to meet the needs of these patients.

For peer review only

Article summary

Strengths and limitations of the study

- This is a large observational study that analysed 1,699,709,314 consultation events from 12,330,545 patients, in 845 general practices, from 1 April 2000 to 31 March 2019.
- The unique contribution of our study is the investigation of distribution trends amongst the top 10% of consulters for the following groups; all consultations (covering all staff working in general practice), all consultations with GPs (including face to face consultations and telephone consultations), face to face consultations with all staff and face to face consultations with GPs.
- Our findings showed that frequent attenders account for an increasing proportion of face-to-face consultations with GPs (38-43%) and are responsible for nearly 40% of all four types of consultations fairly constantly over time.
- This is an observational study, using a database of Electronic Health Records, and it is dependent on the consultations and consultation types being recorded accurately. We were not able to ascertain information on patient and practice characteristics which is critical to understanding why this group of frequent attending patients consult their GPs.
- Future work should examine persistent frequent attendance, and the patient and practice characteristics associated with frequent attendance.

For peer review only

Introduction

General practice in the United Kingdom (UK) has been facing significant workload challenges for more than a decade. In a seminal paper published in 2016, Hobbs and colleagues showed that the crude annual consultation rate per person increased by 10.51%, from 4.67 in 2007-08 to 5.16 in 2013-14.[1] Using data covering nearly 100 million consultations from anonymised practices contributing data to the Clinical Practice Research Datalink (CPRD GOLD), their study found that there was also a substantial increase in average consultation duration and total patient-facing clinical workload (by 16%) during this period. The authors concluded that the perceptions of a rapidly rising workload in UK general practice was well founded and compounded by the complexity of care that general practitioners (GPs) were having to provide including, for example, an increasing elderly population with multiple co-morbidities.

The 2016 report by the King's Fund also confirmed that UK general practice is in crisis with a workload that has increased significantly over time. In addition to indicating that the increase was due to the needs of an ageing population and more people with complex conditions and multimorbidity, it was noted that the effect was compounded by initiatives to transfer care from hospitals to the community.[2]

Although existing work supports the view that GP workload is increasing over time,[1] up-to-date research evidence on workload rates is lacking. One important omission in previous estimates of UK GP workload is that the distribution of consultations within the patient population of general practices has not been assessed. In the Netherlands, Smits and colleagues identified a group of patients, referred to as persistent frequent attenders, occupying the top 10% of age and gender adjusted attenders over three continuous years. This group of frequent attenders may have special health needs, and may require different management in practice due to their distinct clinical and psychological characteristics.[3 4] As most of the literature on frequent attenders has been produced in the Netherlands, the generalisability of these findings and potential solutions in the UK needs to be evaluated. Pilot work in the UK supports these findings and could mean that the top 10% of attenders in the UK general practice are responsible for utilising between 30-50% of all GP consultations.[5] However, there is a paucity of large studies in the UK to establish firm estimates of the prevalence of frequent attendance in the GP workload.

Moreover, working arrangements within UK general practices change rapidly and dynamically. Major reforms are being implemented in response to the 2016 General Practice Forward View which could have an impact on workload rates.[6] For example, tele(e)-consultation options with GPs and other staff members of general practices have become increasingly prevalent, reaching a peak during the Covid-19 pandemic.[7 8] The workforce composition of general practices has also changed as more allied health professionals (e.g. advanced nurse practitioners, clinical pharmacists, and physician associates) and non-clinical staff have been appointed to increase the skill-mix in general practice and better serve the patient needs.[9 10] In addition, the health needs of local populations may affect consultation rates and workload demands differently in sub-regions within the UK .[11 12] Thus, potential variations in the GP workload across geographic regions need to be identified to tailor potential solutions.

For peer review only - http://bmjopen.bmj.com/site/about/guidelines.xhtml

This study provides new information about this under-researched topic by analysing the distribution of consultations within general practices and practice populations. More specifically we examined: a) different ways to describe consultation distributions in a way that is informative to clinical practice; b) how these vary across practices and regions, by consultation type; c) if and how they have changed over time. We also provide contemporary information about consultation rates to update the previous similar analysis undertaken five years ago.[1]

Methods

Data

We used the Clinical Practice Research Datalink (CPRD) GOLD database, which holds anonymised clinical information on patients, from participating general practices, including consultations, diagnoses, treatments, testing and referrals. Consultations include all contacts with the practice and will include requests for repeat prescriptions. They will also include consultations with practice staff (practice nurses, healthcare assistants, social prescribers, nurse practitioners and other allied health professional who are increasingly being employed by GPs. Some patients will contact a practice to speak to reception staff and these are usually identified as administrative episodes so will not be counted as consultations. Non face to face consultations will have included telephone and online consultations.

We analysed consultations data from 1 April 2000 until 31 March 2019, in annual bins of financial years. The number of practices in CPRD GOLD varied from 407 in 2000/1, to 740 in 2008/9, to 389 in 2018/19. Of these, only 113 practices contributed data throughout the whole of the study period. Although the database is representative of the UK in terms of age, gender and deprivation,[13] it is only broadly geographically representative since it collects data from practices using the Vision clinical system, and clinical system usage is geographically clustered in the UK.[14] For example, Scottish general practices are more heavily represented in more recent years of the CPRD database. Socio-economic deprivation of the practice location was available, as measured by the 2015 English Index of Multiple Deprivation (IMD) which is a composite score across seven domains: Income; Employment; Education; Skills and Training; Health and Disability; Crime; Barriers to Housing Services; Living Environment.[15] The Scottish, Welsh and Northern Irish IMDs are very similar, but with small domain changes to capture different country dynamics. Consultation information was aggregated within each financial year, for each active patient (registered for at least one day during the respective year). We aggregated the information into four consultation types: 1) all consultation types (including administrative) by all practice staff (health worker or administrator); 2) all consultation types by GPs; 3) face-to-face consultations by all staff; and 4) face-to-face consultations by all GPs (Supplementary file: Tables 1 & 2).

Statistical analyses

The distribution of consultations within each practice was quantified using various approaches, to allow for within and between practice comparisons over time. First, we calculated the mean, 10th, 25th, 50th (median), 75th and 90th centile of each of the four consultation types, for each practice within each financial year. Next we used the 50th, 75th and 90th centile to aggregate the consultation volume into

For peer review only - http://bmjopen.bmj.com/site/about/guidelines.xhtml

dichotomous patient groups: those with a number of consultations in a given year below the 50th (or 75th or 90th) centile and those with a number of consultations equal to or higher than the 50th (or 75th or 90th) centile. We report the percentage of consultations attributed to the top group (equal to or above 50th, 75th or 90th centile), within each year. Finally, to allow for comparisons across practices, we used an arbitrary threshold of 12 consultations within a year to dichotomise volume, across each consultation type. As for centiles, we report the percentage of practice consultations attributed to people with 12 or more, within each year. The reported percentages, for centiles and 12 consultations, and for each practice within a year, can be expressed as:

$$p_{ij} = 100 * con_{ij1}/(con_{ij1} + con_{ij2})$$

Where:

$i = 1,\ldots,4$ the consultation type
j denotes the 50th, 75th, 90th centile or 12 consultations;
con_{ij1} is the total consultation volume for the top group;
con_{ij2} is the total consultation volume for the bottom group.

Since the groups were not always balanced as per the centiles (e.g. dichotomising along the 50th centile did not result in two equal groups of patients), due to the relative small numbers of consultations for the average patient, we also calculated the ratio of consultations of the top group over the bottom group. This can be expressed as:

$$r_{ij} = {(con_{ij1}/pat_{ij1})}/{(con_{ij2}/pat_{ij2})}$$

Where
pat_{ij1} is the number of patients in the top group;
pat_{ij2} is the number of patients in the bottom group.

We report these metrics in violin plots,[16] across all practices and by region, over time. Violin plots can be interpreted as box plots (including the median as a marker and a box indicating the interquartile range), overlaid with the density of the distribution for better visualisation.

We used negative binomial regression models for key outcomes of interest (50th, 75th, 90th centile and 12 consultations), to examine the association between available practice characteristics and imbalanced consultation distribution. The outcome was the average number of consultations per patient within each of the two groups, for each practice within a year. This approach allowed us to adjust for group sizes, with the resulting analyses corresponding to the ratios previously described. The binary group predictor (top/bottom group) was interacted with each of the following practice characteristics, in separate models: list size (time varying), region, and practice location deprivation as measured by the 2015 Index of Multiple Deprivation.[15]

All analyses were conducted with Stata v16 and an alpha level of 5% was used for the inferential statistics. As a sensitivity, we analysed data only from the 113 practices which were active throughout the whole of the study period.

For peer review only - http://bmjopen.bmj.com/site/about/guidelines.xhtml

For peer review only

Patient and Public Involvement

The research was discussed with a PPI group (PRIMER, based at the Centre for Primary Care and Health Service Research at the University of Manchester) at a meeting held prior to analysis. The Group agreed that understanding consultation patterns was an important starting point for exploring the reasons for frequent attendance (e.g. availability of double appointments for patients with multi-morbidity), the extent to which the needs of frequent attenders are being met and their experience of primary care services.

Results

Overall, 1,699,709,314 consultation events across 12,330,545 patients in 845 general practices were analysed. We focus on the 90th centile analyses in the main paper, splitting the population within a practice to the top 10% in terms of service usage within a year, and contrasting it to the remaining 90% of the practice population. Results were consistent for the other dichotomies (median, 75th centile and 12 or more consultations in a year), with summary results presented in Supplementary file 1. Over time, the average volume of all consultations for a single patient, with GPs and all staff increased. However, face-to-face consultations remained relatively stable and face-to-face consultations with GPs even decreased (Table 1).

Over time, face-to-face consultations per annum with GPs and all staff remained stable for the top 10% group, and in 2018/19 medians (25th & 75th centiles) were 8 (6 & 10) and 12 (10 & 14), respectively (Table 2). On the other hand, medians for all consultations with GPs and all staff greatly increased over time. All consultations with GPs increased from a median (25th & 75th centiles) of 13 (10 & 16) in 2000/1 to 21 (18 & 25) in 2018/19. Similarly, all consultations with all staff increased from 27 (23 & 30) in 2000/1 to 60 (51 & 69) over the study period. However, the picture was somewhat different when quantifying service usage in relative terms, with increases over time across all consultations types for the top 10% group (Table 3 and Figure 1). The percentage of face-to-face consultations attributed to the top 10% of users within a year increased from a median (25th & 75th centiles) of 38.0% (35.9% & 40.3%) to 43.0% (40.6% & 46.4%) for interactions with GPs, and from 35.8% (34.4% & 37.7%) to 40.1% (38.3% & 42.5%) for interactions with all staff. Percentage increases for all consultations were smaller, from a median (25th & 75th centiles) of 38.0 (35.7% & 40.1%) to 40.9 (39.2% & 42.7%) with GPs, and from 35.2 (33.9% & 36.5%) to 36.6 (35.3% & 38.0%) with all staff, over the study period. These increases in service usage for the top 10% group, across all four outcomes, were also reflected in the ratio of consultations, with the largest increase observed in face-to-face consultations with GPs, from a median (25th & 75th centiles) of 4.7 (4.5 & 5.1) in 2000/1 to 5.9 (5.4 & 6.6) in 2018/19 (Table 4 and Figure 2).

There was relatively little regional variability in the median percentage attribution of all four consultation categories, across all examined patient dichotomies. For example, the median percentage of face-to-face consultations with GPs for the top 10% of patients in terms of service usage with their respective practice, varied from 35% for Wales and West Midlands to 38% for Northern Ireland, over the whole study period.

For peer review only - http://bmjopen.bmj.com/site/about/guidelines.xhtml

Results from the negative binomial regression analyses indicated decreasing trends over time for face-to-face consultations and increasing trends for all consultation types, for both GPs and all staff (Table 5 and Figure 3). Adjusted incidence rate ratios (IRRs) for the top 10% consultations group ranged between 4.99 (95% CI: 4.92 to 5.07) for face-to-face consultations with all staff, and 5.60 (95% CI: 5.56 to 5.65) for all consultations with GPs, compared to the bottom 90% consultations group. Associations between deprivation and consultations in the two groups were weak. Some regional variation was observed, with Scotland associated with the highest rate ratios for face-to-face consultations with GPs, and the South West associated with the highest rate ratios for face-to-face consultations with all staff.

Results from analyses of the other dichotomies (75-25, 50-50, 12 or more consultations) and sensitivity analyses focusing on practices for the whole of the study period were broadly similar to our main results.

Discussion

Summary of findings

This study examined distribution trends in four types of consultations (all consultations by GPs; all consultations by all staff; face-to-face consultations by GPs; face-to-face consultations by all staff) among frequent attenders (the top 10% of all consulters) and the rest of consulters in the UK general practices over the period from 2000/1 to 2018/9.

We found that all consultations by GPs and all consultations by all staff have considerably increased over this time period. Specifically, all consultations by GPs per person increased from a median of 5 to 8 and all consultations by all staff increased from 11 to 25. However, face-to-face consultations by GPs and face-to-face consultations by all staff have remained static (and may even have decreased).

Frequent attenders had five times more consultations of any type compared to the rest of consulters over the study period. Among frequent attenders, all consultations by GPs increased from a median of 13 to 21 and all consultations by all staff increased from 27 to 60 over the study period. Approximately 4 out of 10 consultations of any type concerned frequent attenders and the proportion of consultations attributed to frequent attenders increased over time, particularly for face-to-face consultations with GPs (from 38% in 2000/1 to 43% in 2018/9). We found little evidence of regional variability in the attribution of all four consultation types, across all categories of consulters. The only exemption was that the distribution of face-to-face consultations with GPs was highest in Scotland and the distribution of face-to-face consultations with all staff was highest in Northern Ireland.

Research in context

The unique contribution of our study is the investigation of distribution trends in all four types of consultations among frequent attenders (the top 10% of consulters). No previous study in the UK has examined the contribution that frequent attenders make to the workload of general practices. Our

For peer review only - http://bmjopen.bmj.com/site/about/guidelines.xhtml

findings showed that frequent attenders account for an increasing proportion of face-to-face consultations with GPs (38-43%) and are responsible for nearly 40% of all four types of consultations fairly constantly over time. This striking finding suggests that a relatively small number of patients are accounting for a large proportion of GP workload including face-to-face consultations. This could be potentially attributed to a relative reduction in accessibility to face-to-face consultations for non-frequent attenders, which may be driven by a perception of some or all actors (patient/carer/clinician/practice staff) that the need for face-to-face contact is smaller in this patient group. Despite the importance of these findings, there is very little research on frequent attenders in the UK to make direct comparisons. Outside the UK, our findings are consistent with epidemiological studies conducted in the Netherlands which has also shown that frequent attenders were responsible for nearly 40% of the face-to-face consultations.[3] Similarly, a previous review on frequent attenders reported that the top 10% of attenders accounted for 30% to 50% of all primary care consultations.[17]

Our current knowledge of the demographic, clinical and social characteristics of frequent attenders is scarce in the UK whereas findings from Europe are also inconsistent. Our analysis suggests that frequent attenders are identified across all parts of the UK and that deprivation, practice size or regional variation are not drivers for the number of frequent attenders by practice. There is evidence from Europe that frequent attenders (and particularly persistent frequent attenders) are more likely to be female and older, present more social and psychiatric problems, receive more prescriptions of psychotropic medication, have more medically unexplained physical symptoms, and more chronic medical conditions.[3 17]Our study cannot determine whether these factors are the main driving force for frequent attendance but the contribution to overall GP workload needs further examination. A systematic review focused on children also found that frequent attendance in children was associated with presence of psychosocial and mental health problems, younger age, school absence, presence of chronic conditions, and high level of anxiety in their parents.[18] However, another systematic review on older adults found that frequent attendance in late life was mainly associated with presence and severity of chronic medical conditions whereas associations with mental conditions, drug use and social support were heterogeneous.[19] Thus, although there is evidence that frequent attenders differ in terms of demographic, social, mental and physical characteristics from the rest of consulters, their profile may vary across age groups and according to different definitions of frequent attendance (e.g. persistent or no-persistent). Our findings and PPI work strongly support the need for future research to characterise frequent attenders in UK general practice taking into consideration the evidence and challenges encountered by previous studies conducted mostly in Europe.

Moreover, this study confirms and extends the secular trends first identified by Hobbs and colleagues that activity in general practice has increased over time [1]. This previous study found that the crude annual consultation rate per person increased from 4.67 in 2007-08, to 5.16 in 2013-14 and that GP telephone consultation rates doubled, compared with a 5.20% rise in face-to-face consultations. Consistent with this, we found that all consultations by GPs have increased and that face-to-face consultations are not the main driver of this increase. This finding suggests that other modalities for consulting such as telephone or online consultations may have become more important in recent years. Face-to-face consultations may have reached a capacity ceiling and other forms of consultations may be used to address otherwise unmet health needs. Moreover, Hobbs et al [1] found that the greatest increases in rates of consultations were in GPs, with a rise of 12.37% per 10,000 person-years, compared with 0.9% for practice nurses. In our study however, the highest increase over time was

For peer review only - http://bmjopen.bmj.com/site/about/guidelines.xhtml

observed in all consultations by all staff with consultation medians increasing from 11 in 2000/1 to 25 in 2018/9. This impressive increase in all consultations by all staff may suggest that the increase in multi-disciplinary staff working in general practices has resulted in a wider range of practice staff dealing with patients and that it is not all driven by face-to-face consultations. This trend appears to predate the 2016 GP Forward View and is likely to continue.

Limitations

This study has a number of important limitations. First, we used our own definition of frequent attenders (top 10% of consulters) and therefore our findings are only broadly comparable with other studies which have used other definitions (i.e. top 10% of consulters over 3 years; top 25% stratified by age, gender of health conditions). It is reassuring that our definition of frequent attendance is consistent with the recommended definition in a recent systematic review,[20] that our sensitivity analyses showed similar results, and that our findings were similar with the findings from our previous pilot study. Second, patients who have left a general practice but have not been removed from the register (also known as “ghost” patients) may exaggerate the differences between top and bottom users, and we know that their numbers vary by region.[21] This is an observational study, using a database of Electronic Health Records, and it is dependent on the consultations and consultation types being recorded accurately. Since the UK primary care has been almost fully computerised by the start of this century,[22] it is extremely unlikely that any consultations fail to be recorded. However, the default setting in recording a consultation is face-to-face, and this needs to be changed by the health professional for other consultation types. Thus, face-to-face consultations may be overestimated, if some health professionals miss that step. In addition, the underlying workload across consultation types may differ systematically, on top of expected patient variation, but this is something we cannot capture within this study of practice aggregates. Although the CPRD GOLD database is representative in terms of deprivation and population characteristics,[13] it is collecting data from a single computer system (VISION) and the contributing practices are not uniformly distributed across English regions.[14] Thus, generalisability to every English region is questionable, especially in relation to the sensitivity analyses where the sample is quite small and the distribution of the practices across regional and deprivation strata changes. IMD scores are standardised within each UK country, and between country comparisons cannot capture baseline differences across countries. However, our regression analyses on the relative difference between high and low deprivation, in the context of the outcome of interest, should be robust to these nuances. Finally, we have not examined persistent frequent attendance, or the patient or practice characteristics associated with frequent attendance.

Implications for Policy and Practice

Frequent attenders appear to be a major driver for the increase in consultations that have contributed to perceptions of increased workload in general practice. Whilst many of these patients may have co-morbidities and may need to be seen regularly, research suggest that they have wider social and psychological needs.[17] GPs should be looking at this group of patients more closely to understand who are they and why are they consulting more frequently. Our PPI work indicates that patients see this as critically important for improving quality of care and preventing patients from being stigmatised for frequent attendance. One key challenge for future policy is how can general practice be prepared to provide services and support to frequent attenders without medicalising them but addressing the wider social and psychological problems that they may have.

For peer review only - http://bmjopen.bmj.com/site/about/guidelines.xhtml

The large increase in the general practice workload the last 20 years means that having extended multidisciplinary teams to meet a wide range of patient needs through a range of ways (e.g. remote consultations) is perhaps the only solution for sustaining a viable primary care. This change in the workforce composition of general practices is reflected in the activities that we report (medians for all consultations by GPs increased 5 to 8 whereas all consultations by all staff increased from 11 to 25) and echoes recent policy documents such as the 2016 General Practice Forward View. It is also likely that a multidisciplinary workforce in general practice could also better address the wider, not always medical, needs of frequent attenders.

Conclusions

This is the first study to show that frequent attenders, a relatively small group of consulters (the top 10%), largely and progressively contribute to increased workload in general practices across the UK over the last 20 years. Important knowledge gaps remain in terms of the demographic, social and health characteristics of frequent attenders in the UK and how general practices can be prepared to meet the needs of these patients. We intend to explore these factors in a separate study. Our findings depict a new model of work in general practice whereby an increasing number of consultations are conducted by other staff members (rather than GPs) using alternative means (rather than face-to-face consultations). With the overall workload of general practices on continuous rise, multidisciplinary and technology-enabled general practices could better meet the multiple and wider needs of frequent attenders and the rest of consulters.

For peer review only

For peer review only - http://bmjopen.bmj.com/site/about/guidelines.xhtml

Acknowledgements

We thank the members of the PRIMER patient and public involvement group (funded through the NIHR School for Primary Care Research) for their input into the early stages of this research and to Julie Billsborough, Manoj Mistry, Carole Bennett and Ailsa Donnelly for their contributions.

Ethical approval

This study is based on data from the Clinical Practice Research Datalink GOLD obtained under license from the Medicines and Healthcare Products Regulatory Agency (MHRA). The data are provided by patients and collected by the NHS as part of their care and support. The interpretation and conclusions contained in this study are those of the authors alone, and not necessarily those of the MHRA, the National Institute for Health Research (NIHR), National Health Service (NHS), or the Department of Health. Approval to conduct this study using the CPRD was granted by the Independent Scientific Advisory Committee (ISAC) of the Medicine and Healthcare Regulatory Agency (MHRA) (protocol 20_192R). We thank the contributing patients and practices to the CPRD GOLD who have allowed their data to be used for research purposes.

Funding

The University of Manchester. The funder had no role in any aspect of this work.

Data sharing statement

The data analysed in this study are subject to the following licenses/restrictions: Electronic health records are, by definition, considered "sensitive" data in the UK by the Data Protection Act and cannot be shared via public deposition because of information governance restriction in place to protect patient confidentiality. Access to data are available only once approval has been obtained through the individual constituent entity controlling access to the data. The primary care data can be requested via application to the Clinical Practice Research Datalink (www.cprd.com). Requests to access this dataset should be directed to enquiries@cprd.com.

Competing interests statement

All authors have completed the ICMJE uniform disclosure form and declare: support from the University of Manchester for the submitted work; no financial relationships with any organisations that might have an interest in the submitted work in the previous three years, no other relationships or activities that could appear to have influenced the submitted work.

Contributorship statement

The research idea originated with AE and AT, but all authors designed the study. EK extracted the data and conducted the analyses, LM and RP supported him in this. TF led the ISAC submission, for access to the CPRD GOLD database. EK, MP and AE drafted the manuscript, TF, LM, RP, CP, SS, AT and DA critically revised it.

For peer review only - http://bmjopen.bmj.com/site/about/guidelines.xhtml

Author Licence

I, the Submitting Author has the right to grant and does grant on behalf of all authors of the Work (as defined in the author licence), an exclusive licence and/or a non-exclusive licence for contributions from authors who are: i) UK Crown employees; ii) where BMJ has agreed a CC-BY licence shall apply, and/or iii) in accordance with the terms applicable for US Federal Government officers or employees acting as part of their official duties; on a worldwide, perpetual, irrevocable, royalty-free basis to BMJ Publishing Group Ltd ("BMJ") its licensees.

The Submitting Author accepts and understands that any supply made under these terms is made by BMJ to the Submitting Author unless you are acting as an employee on behalf of your employer or a postgraduate student of an affiliated institution which is paying any applicable article publishing charge ("APC") for Open Access articles. Where the Submitting Author wishes to make the Work available on an Open Access basis (and intends to pay the relevant APC), the terms of reuse of such Open Access shall be governed by a Creative Commons licence – details of these licences and which licence will apply to this Work are set out in our licence referred to above.

For peer review only

References

1. Hobbs FDR, Bankhead C, Mukhtar T, Stevens S, Perera-Salazar R, Holt T, et al. Clinical workload in UK primary care: a retrospective analysis of 100 million consultations in England, 2007-14. *Lancet* 2016;387:2323-30.
2. Baird B, Charles A, Honeyman M, Maguire D, Das P. *Understanding pressures in general practice*: King's Fund London, 2016.
3. Smits FT, Brouwer HJ, ter Riet G, van Weert HC. Epidemiology of frequent attenders: a 3-year historic cohort study comparing attendance, morbidity and prescriptions of one-year and persistent frequent attenders. *BMC Public Health* 2009;9:36.
4. Smits FT, Brouwer HJ, Zwinderman AH, Mohrs J, Smeets HM, Bosmans JE, et al. Morbidity and doctor characteristics only partly explain the substantial healthcare expenditures of frequent attenders: a record linkage study between patient data and reimbursements data. *BMC Fam Pract* 2013;14:138.
5. Patel S, Kai J, Atha C, Avery A, Guo B, James M, et al. Clinical characteristics of persistent frequent attenders in primary care: case-control study. *Fam Pract* 2015;32:624-30.
6. England N. General practice forward view. 2016.
7. Salisbury H. Helen Salisbury: Teleconsultations for all. *BMJ* 2020;370:m3211.
8. Salisbury C, Murphy M, Duncan P. The Impact of Digital-First Consultations on Workload in General Practice: Modeling Study. *J Med Internet Res* 2020;22:e18203.
9. Nelson P, Martindale AM, McBride A, Checkland K, Hodgson D. Skill-mix change and the general practice workforce challenge. *Br J Gen Pract* 2018;68:66-67.
10. Nelson PA, Bradley F, Martindale AM, McBride A, Hodgson D. Skill-mix change in general practice: a qualitative comparison of three 'new' non-medical roles in English primary care. *Br J Gen Pract* 2019;69:e489-e98.
11. Kontopantelis E, Mamas MA, van Marwijk H, Ryan AM, Bower P, Guthrie B, et al. Chronic morbidity, deprivation and primary medical care spending in England in 2015-16: a cross-sectional spatial analysis. *BMC Med* 2018;16:19.
12. Spooner S, Gibson J, Checkland K, McBride A, Hodgson DE, Hann M, et al. Regional variation in practitioner employment in general practices in England: a comparative analysis. *Br J Gen Pract* 2020;70:e164-e71.
13. Herrett E, Gallagher AM, Bhaskaran K, Forbes H, Mathur R, van Staa T, et al. Data Resource Profile: Clinical Practice Research Datalink (CPRD). *Int J Epidemiol* 2015;44:827-36.
14. Kontopantelis E, Stevens RJ, Helms PJ, Edwards D, Doran T, Ashcroft DM. Spatial distribution of clinical computer systems in primary care in England in 2016 and implications for primary care electronic medical record databases: a cross-sectional population study. *BMJ Open* 2018;8:e020738.
15. Kontopantelis E, Mamas MA, van Marwijk H, Ryan AM, Buchan IE, Ashcroft DM, et al. Geographical epidemiology of health and overall deprivation in England, its changes and persistence from 2004 to 2015: a longitudinal spatial population study. *J Epidemiol Community Health* 2018;72:140-47.
16. Winter N. VIOPLOT: Stata module to produce violin plots with current graphics. 2008.
17. Vedsted P, Christensen MB. Frequent attenders in general practice care: a literature review with special reference to methodological considerations. *Public Health* 2005;119:118-37.
18. Al-Saffar MN, Hayhoe BW, Harris MJ, Majeed A, Greenfield G. Children as frequent attenders in primary care: a systematic review. *BJGP Open* 2020;4.
19. Welzel FD, Stein J, Hajek A, Konig HH, Riedel-Heller SG. Frequent attenders in late life in primary care: a systematic review of European studies. *BMC Fam Pract* 2017;18:104.
20. Luciano JV, Fernandez A, Pinto-Meza A, Lujan L, Bellon JA, Garcia-Campayo J, et al. Frequent attendance in primary care: comparison and implications of different definitions. *Br J Gen Pract* 2010;60:49-55.

For peer review only - http://bmjopen.bmj.com/site/about/guidelines.xhtml

21. Burch P, Doran T, Kontopantelis E. Regional variation and predictors of over-registration in English primary care in 2014: a spatial analysis. *J Epidemiol Community Health* 2018;72:532-38.
22. Kontopantelis E, Buchan I, Reeves D, Checkland K, Doran T. Relationship between quality of care and choice of clinical computing system: retrospective analysis of family practice performance under the UK's quality and outcomes framework. *BMJ Open* 2013;3.

For peer review only

For peer review only - http://bmjopen.bmj.com/site/about/guidelines.xhtml

Tables

Table 1 Medians (25th – 75th centiles) of average number of consultations per patient, by consultation type, all practices

Year	All consultations by all staff	All consultations by GPs	Face-to-face consultations by all staff	Face-to-face consultations by GPs
**2000-2001**	11.4 (9.8-13.0)	5.3 (4.3-6.8)	5.2 (4.5-6.3)	3.7 (3.2-4.7)
**2001-2002**	12.5 (10.5-14.2)	5.3 (4.4-6.4)	5.2 (4.5-6.4)	3.6 (3.1-4.4)
**2002-2003**	13.4 (11.2-15.7)	5.3 (4.3-6.6)	5.1 (4.3-6.4)	3.5 (2.9-4.3)
**2003-2004**	14.8 (12.0-17.3)	5.7 (4.6-6.9)	5.1 (4.3-6.8)	3.4 (2.8-4.2)
**2004-2005**	15.8 (12.5-18.3)	5.9 (4.9-7.1)	5.0 (4.1-6.7)	3.2 (2.7-4.2)
**2005-2006**	17.0 (13.8-20.1)	6.3 (5.4-7.6)	5.3 (4.4-7.0)	3.3 (2.8-4.3)
**2006-2007**	17.3 (14.0-20.5)	6.3 (5.3-7.7)	5.1 (4.2-6.9)	3.2 (2.7-4.1)
**2007-2008**	18.4 (15.3-21.2)	6.5 (5.4-7.7)	5.2 (4.3-6.6)	3.3 (2.8-4.1)
**2008-2009**	19.4 (16.5-22.4)	6.8 (5.6-7.9)	5.2 (4.4-6.4)	3.3 (2.8-3.9)
**2009-2010**	20.3 (17.7-23.3)	7.1 (5.9-8.4)	5.3 (4.5-6.4)	3.4 (2.8-4.0)
**2010-2011**	20.4 (17.8-23.8)	7.3 (6.1-8.4)	5.3 (4.4-6.3)	3.4 (2.8-4.0)
**2011-2012**	21.3 (18.5-24.6)	7.6 (6.4-8.8)	5.4 (4.5-6.4)	3.4 (2.8-4.0)
**2012-2013**	21.8 (19.0-25.2)	7.6 (6.6-9.1)	5.4 (4.6-6.4)	3.4 (2.9-4.1)
**2013-2014**	22.8 (19.7-26.3)	8.0 (6.9-9.5)	5.4 (4.6-6.4)	3.4 (2.8-4.1)
**2014-2015**	23.4 (20.3-26.7)	8.2 (6.9-9.7)	5.4 (4.6-6.3)	3.3 (2.8-4.1)
**2015-2016**	23.2 (20.0-26.8)	7.9 (6.6-9.3)	5.1 (4.3-6.1)	3.2 (2.6-3.9)
**2016-2017**	23.4 (20.3-27.4)	8.3 (6.8-9.7)	5.2 (4.3-6.2)	3.3 (2.6-4.0)
**2017-2018**	23.8 (20.7-27.4)	8.2 (7.0-9.7)	5.1 (4.1-6.1)	3.2 (2.5-4.0)
**2018-2019**	25.1 (21.7-28.7)	8.3 (7.0-9.7)	5.0 (4.2-6.0)	3.1 (2.5-3.9)

For peer review only

For peer review only - http://bmjopen.bmj.com/site/about/guidelines.xhtml

Table 2 Medians (25th – 75th centiles) of 90th centile number of consultations per patient, by consultation type, all practices

**Year**	**All consultations by all staff**	**All consultations by GPs**	**Face-to-face consultations by all staff**	**Face-to-face consultations by GPs**
**2000-2001**	27 (23-30)	13 (10-16)	12 (10-15)	9 (8-11)
**2001-2002**	30 (25-34)	13 (10-16)	12 (10-15)	8 (7-11)
**2002-2003**	32 (26-37)	13 (10-16)	12 (10-15)	8 (7-10)
**2003-2004**	35 (29-41)	14 (12-17)	12 (10-16)	8 (7-10)
**2004-2005**	38 (30-45)	15 (12-18)	12 (10-16)	8 (7-10)
**2005-2006**	41 (33-49)	16 (13-19)	12 (10-17)	8 (7-10)
**2006-2007**	42 (34-50)	16 (13-19)	12 (10-16)	8 (7-10)
**2007-2008**	44 (37-52)	16 (13-19)	12 (10-15)	8 (7-10)
**2008-2009**	47 (40-54)	17 (14-20)	12 (10-15)	8 (7-10)
**2009-2010**	49 (42-56)	18 (15-21)	12 (11-15)	8 (7-10)
**2010-2011**	49 (43-57)	18 (15-21)	12 (10-15)	8 (7-10)
**2011-2012**	51 (44-58)	19 (16-22)	13 (11-15)	8 (7-10)
**2012-2013**	52 (45-60)	19 (16-23)	13 (11-15)	8 (7-10)
**2013-2014**	55 (47-63)	20 (17-24)	13 (11-15)	8 (7-10)
**2014-2015**	56 (49-64)	20 (17-24)	13 (11-15)	8 (7-10)
**2015-2016**	55 (48-64)	20 (17-23)	13 (11-15)	8 (7-10)
**2016-2017**	56.5 (48-66)	21 (17-24)	12.5 (10-15)	8 (7-10)
**2017-2018**	57 (50-66)	20 (17-24)	12 (10-15)	8 (6-10)
**2018-2019**	60 (51-69)	21 (18-25)	12 (10-14)	8 (6-10)

For peer review only

For peer review only - http://bmjopen.bmj.com/site/about/guidelines.xhtml

Table 3 Medians (25th – 75th centiles) of proportion of high volume (≥ 90th centile) per patient, by consultation type, all practices

Year	All consultations by all staff	All consultations by GPs	Face-to-face consultations by all staff	Face-to-face consultations by GPs
**2000-2001**	35.2 (33.9-36.5)	38.0 (35.7-40.1)	35.8 (34.4-37.7)	38.0 (35.9-40.3)
**2001-2002**	35.5 (34.1-36.8)	38.4 (36.5-40.4)	36.3 (34.6-38.2)	38.5 (36.5-40.9)
**2002-2003**	35.6 (34.3-37.0)	38.7 (36.6-41.1)	36.5 (35.0-38.4)	38.5 (36.4-41.2)
**2003-2004**	35.9 (34.5-37.2)	39.1 (37.2-41.5)	36.9 (35.2-39.0)	39.0 (36.7-41.7)
**2004-2005**	36.5 (35.0-37.9)	40.1 (37.8-42.4)	37.9 (35.9-40.3)	40.1 (37.6-43.3)
**2005-2006**	36.1 (34.9-37.6)	39.8 (37.6-41.9)	37.5 (35.7-39.7)	39.8 (37.3-42.5)
**2006-2007**	36.7 (35.2-38.1)	40.5 (38.2-42.8)	38.3 (36.3-40.7)	40.7 (38.2-44.2)
**2007-2008**	36.5 (35.1-37.9)	40.0 (38.2-42.1)	37.9 (36.1-40.0)	39.9 (37.9-42.9)
**2008-2009**	36.6 (35.3-37.9)	40.2 (38.3-42.1)	38.1 (36.5-40.0)	40.1 (37.8-43.1)
**2009-2010**	36.3 (35.1-37.7)	40.0 (38.4-41.9)	38.0 (36.3-40.0)	40.3 (38.1-43.0)
**2010-2011**	36.7 (35.3-38.0)	40.1 (38.5-42.0)	38.3 (36.6-40.5)	40.6 (38.3-43.1)
**2011-2012**	36.9 (35.5-38.1)	40.4 (38.7-42.1)	38.6 (37.0-40.6)	40.9 (38.6-43.5)
**2012-2013**	36.9 (35.5-38.2)	40.3 (38.7-41.9)	38.6 (37.1-40.5)	40.9 (38.7-43.3)
**2013-2014**	36.8 (35.5-38.3)	40.5 (39.1-42.2)	38.9 (37.2-40.7)	41.3 (39.4-43.6)
**2014-2015**	37.1 (35.6-38.5)	40.8 (39.3-42.5)	39.4 (37.5-41.4)	41.5 (39.3-44.0)
**2015-2016**	36.6 (35.3-38.2)	41.4 (39.7-43.7)	40.4 (38.4-42.7)	43.1 (40.4-46.3)
**2016-2017**	36.8 (35.6-38.4)	41.0 (39.6-43.0)	40.1 (38.1-42.4)	42.5 (40.3-45.4)
**2017-2018**	36.8 (35.3-38.3)	40.9 (39.1-42.9)	40.1 (38.1-42.2)	42.8 (40.1-45.4)
**2018-2019**	36.6 (35.3-38.0)	40.9 (39.2-42.7)	40.1 (38.3-42.5)	43.0 (40.6-46.4)

For peer review only

Table 4: Medians (25th – 75th centiles) of ratio for users ≥90th centile over the rest of consultations per patient, by consultation type, all practices

Year	All consultations by all staff	All consultations by GPs	Face-to-face consultations by all staff	Face-to-face consultations by GPs
**2000-2001**	4.6 (4.4-4.9)	4.9 (4.6-5.4)	4.5 (4.3-4.8)	4.7 (4.5-5.1)
**2001-2002**	4.7 (4.5-5.0)	5.0 (4.7-5.5)	4.6 (4.4-4.9)	4.8 (4.5-5.2)
**2002-2003**	4.7 (4.5-5.1)	5.2 (4.8-5.6)	4.7 (4.4-5.0)	4.9 (4.6-5.3)
**2003-2004**	4.8 (4.6-5.1)	5.3 (5.0-5.8)	4.8 (4.5-5.1)	5.0 (4.7-5.5)
**2004-2005**	5.0 (4.7-5.3)	5.5 (5.1-6.1)	5.0 (4.6-5.4)	5.2 (4.8-5.8)
**2005-2006**	4.9 (4.6-5.2)	5.5 (5.1-6.0)	4.8 (4.6-5.3)	5.1 (4.8-5.7)
**2006-2007**	5.0 (4.7-5.4)	5.7 (5.2-6.3)	5.1 (4.7-5.6)	5.4 (4.9-6.0)
**2007-2008**	5.0 (4.7-5.3)	5.6 (5.1-6.0)	5.0 (4.7-5.4)	5.2 (4.8-5.7)
**2008-2009**	5.0 (4.7-5.4)	5.6 (5.2-6.0)	5.0 (4.7-5.4)	5.3 (4.9-5.7)
**2009-2010**	5.0 (4.7-5.3)	5.6 (5.2-6.0)	5.0 (4.7-5.4)	5.2 (4.9-5.7)
**2010-2011**	5.1 (4.8-5.4)	5.7 (5.3-6.1)	5.1 (4.7-5.5)	5.3 (4.9-5.8)
**2011-2012**	5.1 (4.8-5.4)	5.7 (5.3-6.1)	5.1 (4.8-5.6)	5.4 (5.0-5.8)
**2012-2013**	5.1 (4.8-5.4)	5.7 (5.4-6.1)	5.2 (4.8-5.6)	5.4 (5.0-5.8)
**2013-2014**	5.1 (4.8-5.5)	5.8 (5.4-6.2)	5.2 (4.9-5.6)	5.5 (5.1-6.0)
**2014-2015**	5.2 (4.9-5.5)	5.9 (5.5-6.3)	5.3 (5.0-5.8)	5.6 (5.2-6.1)
**2015-2016**	5.1 (4.8-5.5)	6.0 (5.6-6.5)	5.6 (5.1-6.2)	5.9 (5.4-6.6)
**2016-2017**	5.1 (4.9-5.5)	5.9 (5.5-6.4)	5.5 (5.1-6.0)	5.8 (5.3-6.4)
**2017-2018**	5.1 (4.8-5.5)	5.9 (5.5-6.3)	5.5 (5.1-6.0)	5.8 (5.4-6.5)
**2018-2019**	5.1 (4.8-5.4)	5.9 (5.5-6.3)	5.5 (5.1-6.0)	5.9 (5.4-6.6)

For peer review only

For peer review only - http://bmjopen.bmj.com/site/about/guidelines.xhtml

Table 5: Associations between covariates of interest and consultation volume, incidence rate ratios (and 95% CIs) from main negative binomial regression model

	All consultations – all staff	All consultations – GPs	Face-to-face consultations – all staff	Face-to-face consultations - GPs
Year	1.046 (1.045,1.047)	1.028 (1.028,1.029)	0.995 (0.994,0.997)	0.988 (0.986,0.989)
List size (per 1000 patients)	1.003 (1.002,1.004)	0.997 (0.996,0.998)	1.001 (0.998,1.003)	0.992 (0.990,0.995)
Consultation group				
Bottom 90%	Reference category			
Top 10%	5.038 (5.007,5.068)	5.603 (5.560,5.647)	4.992 (4.917,5.068)	5.253 (5.171,5.337)
IMD Deprivation quintile				
(least deprived) 1	Reference category			
2	1.005 (0.994,1.016)	1.019 (1.005,1.033)	0.993 (0.967,1.020)	1.044 (1.015,1.073)
3	1.048 (1.037,1.059)	1.049 (1.035,1.063)	1.038 (1.011,1.065)	1.076 (1.047,1.106)
4	1.019 (1.009,1.030)	1.046 (1.032,1.060)	0.981 (0.956,1.006)	1.058 (1.030,1.087)
(most deprived) 5	1.025 (1.015,1.036)	0.993 (0.980,1.006)	0.997 (0.972,1.023)	1.007 (0.981,1.035)
Region				
North East	Reference category			
North West	0.926 (0.899,0.953)	0.995 (0.958,1.034)	1.033 (0.961,1.111)	0.985 (0.913,1.063)
Yorkshire & Humber	0.952 (0.920,0.985)	1.028 (0.984,1.075)	1.023 (0.940,1.113)	1.003 (0.918,1.096)
East Midlands	0.898 (0.867,0.930)	0.912 (0.872,0.955)	0.963 (0.883,1.050)	0.915 (0.835,1.002)
West Midlands	0.840 (0.815,0.865)	1.034 (0.994,1.074)	1.074 (0.998,1.157)	1.065 (0.986,1.150)
East of England	0.853 (0.827,0.880)	0.946 (0.909,0.985)	1.012 (0.937,1.092)	0.960 (0.886,1.040)
South West	0.954 (0.926,0.983)	1.085 (1.043,1.128)	1.256 (1.166,1.353)	1.080 (0.999,1.168)
South Central	0.844 (0.819,0.870)	0.952 (0.916,0.990)	1.100 (1.021,1.186)	1.052 (0.972,1.138)
London	0.738 (0.716,0.760)	0.922 (0.887,0.958)	0.895 (0.832,0.964)	0.918 (0.850,0.992)
South East Coast	0.830 (0.805,0.855)	0.928 (0.893,0.964)	0.987 (0.918,1.063)	1.000 (0.926,1.080)
Northern Ireland	1.150 (1.115,1.187)	1.446 (1.389,1.506)	1.087 (1.006,1.174)	1.128 (1.040,1.223)
Scotland	0.785 (0.763,0.808)	1.042 (1.004,1.081)	1.249 (1.163,1.340)	1.207 (1.121,1.300)
Wales	0.883 (0.858,0.909)	0.969 (0.934,1.006)	1.045 (0.973,1.123)	1.007 (0.934,1.086)

For peer review only - http://bmjopen.bmj.com/site/about/guidelines.xhtml

Figures

Figure 1: Percentage of consultations by people consulting at the 90th centile or above within a financial year, over time

Figure 2: Ratio of consultations for people ≥90th centile vs those below within a financial year, over time

Figure 3: Consultation count estimates by group over time (Predicted incidence rates from the negative binomial regression model with a time-group interaction term)*

* Interpreted as the estimated average number of consultations for a patient within each group

For peer review only

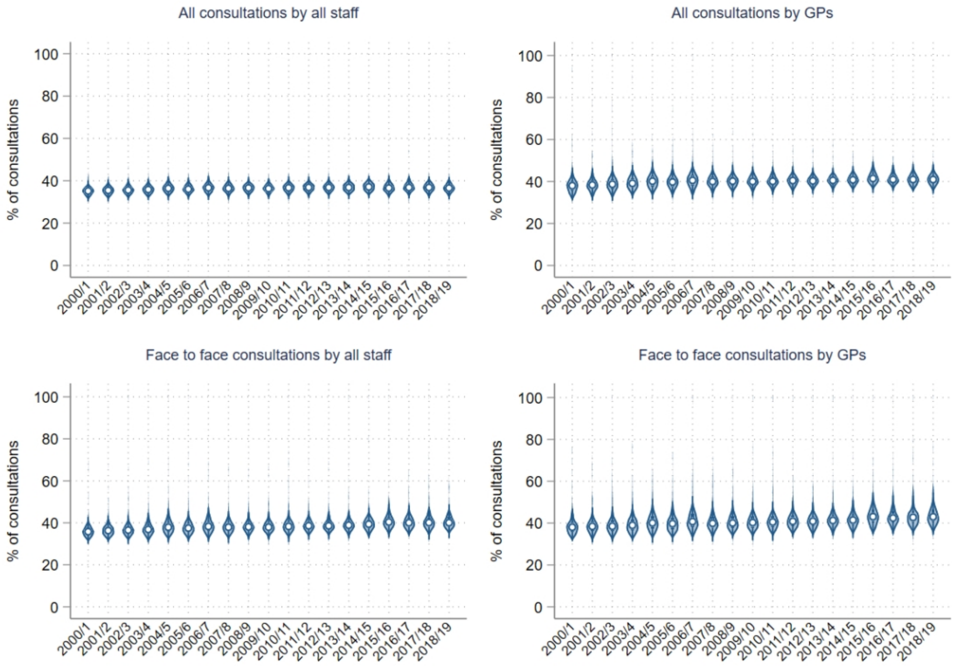

423x302mm (72 x 72 DPI)

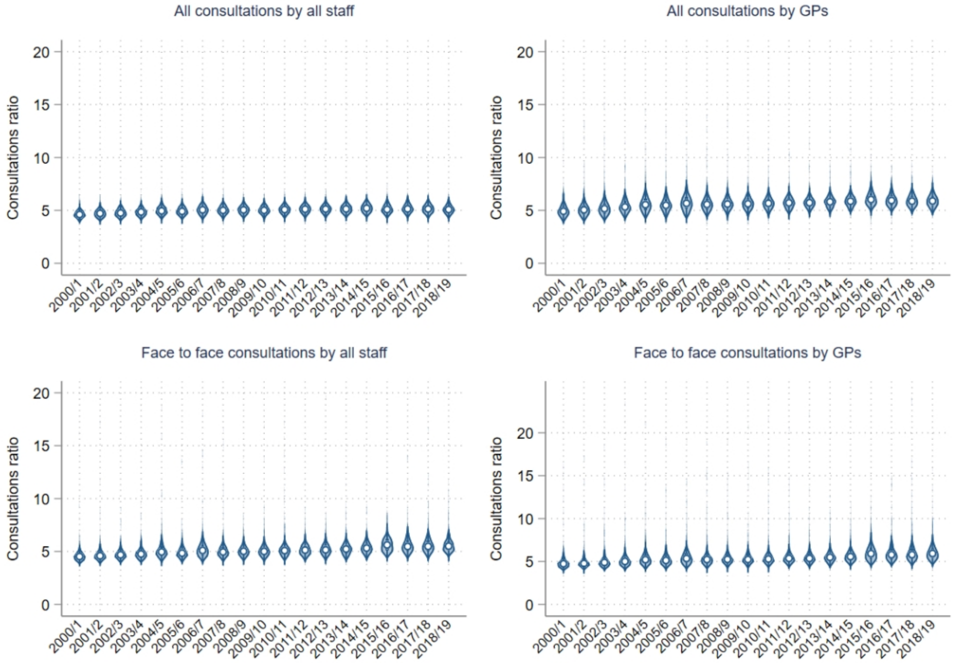

423x302mm (72 x 72 DPI)

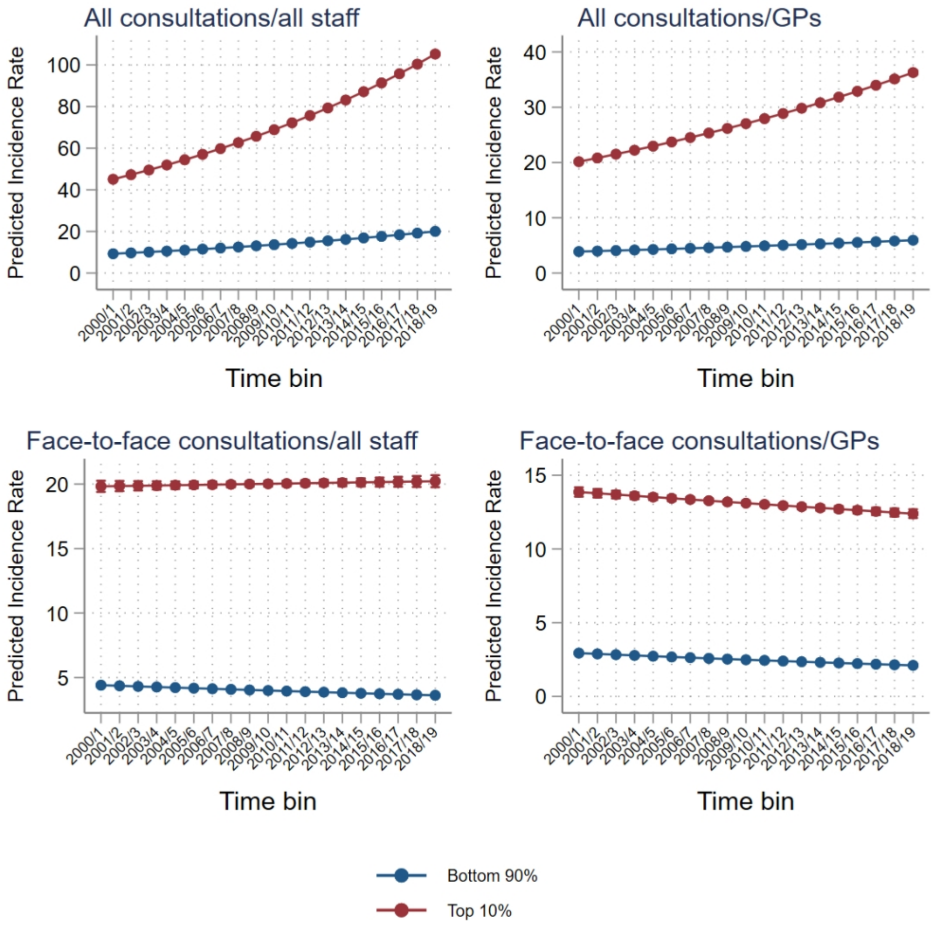

423x423mm (72 x 72 DPI)

Supplementary file to "Consultation patterns and frequent attenders in UK primary care from 2000 to 2019: a retrospective analysis of 1,699,709,314 consultation events across 12,330,545 patients in 845 general practices"

Defining the consultation groups

GPs defined as: 1,2,3,4,5,6,7,8,9,10,47,50 in Table 1 (in red font).

Table 1: Staff Role

Code	Description
0	Data Not Entered
1	Senior Partner
2	Partner
3	Assistant
4	Associate
5	Non-commercial local rota of less than 10 GPs
6	Commercial Deputising service
7	Locum
8	GP Registrar
9	Consultant
10	Sole Practitioner
11	Practice Nurse
12	Health Visitor
13	Community Nurse
14	Midwife
15	Community Psychiatric Nurse
16	Social Worker
17	Pharmacist
18	Dispenser
19	Non-qualified Dispenser
20	Practice Manager
21	Fund Manager
22	Business Manager
23	Administrator
24	Secretary
25	Receptionist
26	Physiotherapist
27	Chiropodist
28	Dentist
29	Dietician
30	Counsellor
31	Osteopath
32	Maintenance staff
33	Other Health Care Professional
34	Hospital Nurse

For peer review only - http://bmjopen.bmj.com/site/about/guidelines.xhtml

Code	Description
35	Community Medical Officer
36	School Nurse
37	Health Education Officer
38	Contact Tracing Nurse
39	Stomatherapist
40	Computer Manager
41	Interpreter/Link Worker
42	Chiropractor
43	Acupuncturist
44	Homeopath
45	Mental Handicap Nurse
46	Carer
47	Salaried Partner
48	Occupational Therapist
49	Speech Therapist
50	GP Retainer
51	Phlebotomist
52	Other Medical & Dental
53	Other Students
54	Other Nursing & Midwifery
55	Other Allied Health Professionals
56	Other Professional Scientific & Technical
57	Other Healthcare Scientists
58	Other Additional Clinical Services
59	Other Admin & Clerical
60	Clinical Practitioner Access Role
61	Nurse Access Role
62	Nurse Manager Access Role
63	Health Professional Access Role
64	Healthcare Student Access Role
65	Biomedical Scientist Access Role
66	Clinical Coder Access Role
67	Optometrist
68	Radiographer

Face-to-face consultations defined as 1,2,3,4,6,7,8,9,11,18,24,27,28,30,31,32,33,34,36,37,38,50,61 in Table 2 (in red font).

Table 2: Consultation type

Code	Description
0	Data Not Entered
1	Clinic
2	Night visit, Deputising service
3	Follow-up/routine visit
4	Night visit, Local rota
5	Mail from patient
6	Night visit , practice

For peer review only - http://bmjopen.bmj.com/site/about/guidelines.xhtml

Code	Description
7	Out of hours, Practice
8	Out of hours, Non Practice
9	Surgery consultation
10	Telephone call from a patient
11	Acute visit
12	Discharge details
13	Letter from Outpatients
14	Repeat Issue
15	Other
16	Results recording
17	Mail to patient
18	Emergency Consultation
19	Administration
20	Casualty Attendance
21	Telephone call to a patient
22	Third Party Consultation
23	Hospital Admission
24	Children's Home Visit
25	Day Case Report
26	GOS18 Report
27	Home Visit
28	Hotel Visit
29	NHS Direct Report
30	Nursing Home Visit
31	Residential Home Visit
32	Twilight Visit
33	Triage
34	Walk-in Centre
35	Co-op Telephone advice
36	Co-op Surgery Consultation
37	Co-op Home Visit
38	Minor Injury Service
39	Medicine Management
40	Community Clinic
41	Community Nursing Note
42	Community Nursing Report
43	Data Transferred from other system
44	Health Authority Entry
45	Health Visitor Note
46	Health Visitor Report
47	Hospital Inpatient Report
48	Initial Post Discharge Review
49	Laboratory Request
50	Night Visit
51	Radiology Request
52	Radiology Result
53	Referral Letter

For peer review only

Code	Description
54	Social Services Report
55	Telephone Consultation
56	Template Entry
57	GP to GP communication transaction
58	Non-consultation medication data
59	Non-consultation data
60	ePharmacy message
61	Extended Hours

For peer review only

For peer review only - http://bmjopen.bmj.com/site/about/guidelines.xhtml

Median (50th centile)

Table 3 Medians (25th – 75th centiles) of median number of consultations by consultation type, all practices

Year	All consultations by all staff	All consultations by GPs	Face-to-face consultations by all staff	Face-to-face consultations by GPs
**2000-2001**	7 (6-8)	3 (3-4)	4 (3-4)	2 (2-3)
**2001-2002**	8 (7-9)	3 (3-4)	4 (3-4)	2 (2-3)
**2002-2003**	9 (7-10)	3 (3-4)	3 (3-4)	2 (2-3)
**2003-2004**	9 (7-11)	3 (3-4)	3 (3-4)	2 (2-3)
**2004-2005**	9 (7-11)	3 (3-4)	3 (3-4)	2 (2-3)
**2005-2006**	10 (8-13)	4 (3-4)	4 (3-4)	2 (2-3)
**2006-2007**	10 (8-13)	4 (3-4)	3 (3-4)	2 (2-3)
**2007-2008**	11 (9-13)	4 (3-5)	3 (3-4)	2 (2-3)
**2008-2009**	12 (10-14)	4 (3-5)	3 (3-4)	2 (2-3)
**2009-2010**	13 (11-15)	4 (3-5)	4 (3-4)	2 (2-3)
**2010-2011**	13 (11-15)	4 (3-5)	3 (3-4)	2 (2-3)
**2011-2012**	13 (11-15)	4 (4-5)	3 (3-4)	2 (2-3)
**2012-2013**	14 (12-16)	4 (4-5)	4 (3-4)	2 (2-3)
**2013-2014**	14 (12-17)	5 (4-5)	4 (3-4)	2 (2-3)
**2014-2015**	14 (12-17)	5 (4-6)	3 (3-4)	2 (2-3)
**2015-2016**	14 (12-17)	4 (3-5)	3 (3-4)	2 (1-2)
**2016-2017**	15 (12-17)	5 (4-6)	3 (3-4)	2 (1-2)
**2017-2018**	15 (13-18)	5 (4-6)	3 (3-4)	2 (1-2)
**2018-2019**	16 (13-18)	5 (4-6)	3 (2-4)	2 (1-2)

For peer review only

Table 4 Medians (25th – 75th centiles) of proportion of high volume (≥ 50th centile) by consultation type, all practices

Year	All consultations by all staff	All consultations by GPs	Face-to-face consultations by all staff	Face-to-face consultations by GPs
**2000-2001**	87.0 (86.2-87.8)	90.2 (88.2-92.4)	87.9 (86.0-90.1)	91.6 (87.9-94.0)
**2001-2002**	87.3 (86.4-88.1)	90.3 (88.4-92.5)	88.4 (86.8-90.4)	92.2 (88.7-94.0)
**2002-2003**	87.4 (86.6-88.2)	90.8 (89.1-93.0)	88.8 (87.2-90.5)	92.7 (89.2-94.2)
**2003-2004**	87.6 (86.9-88.4)	90.9 (89.3-92.8)	89.1 (87.5-90.8)	92.7 (89.6-94.1)
**2004-2005**	88.3 (87.5-89.1)	91.8 (89.7-93.9)	90.2 (88.4-92.6)	93.3 (90.5-94.9)
**2005-2006**	88.0 (87.2-88.8)	91.5 (89.7-93.2)	89.5 (87.8-91.5)	92.9 (90.3-94.4)
**2006-2007**	88.4 (87.5-89.3)	92.1 (90.1-94.1)	90.7 (88.5-92.7)	93.4 (91.0-95.2)
**2007-2008**	88.1 (87.2-88.9)	91.5 (89.9-93.0)	89.7 (87.9-91.7)	93.0 (90.7-94.5)
**2008-2009**	88.1 (87.3-88.8)	91.5 (90.0-93.0)	90.0 (88.3-91.9)	93.4 (91.2-94.8)
**2009-2010**	87.9 (87.2-88.7)	91.3 (90.0-93.0)	89.8 (88.2-91.9)	93.2 (90.8-94.7)
**2010-2011**	88.0 (87.3-88.8)	91.4 (90.0-93.0)	90.0 (88.3-92.0)	93.3 (91.2-94.8)
**2011-2012**	88.1 (87.4-88.8)	91.4 (90.1-92.6)	90.2 (88.6-92.0)	93.3 (91.4-95.0)
**2012-2013**	88.1 (87.4-88.9)	91.4 (90.1-92.6)	90.0 (88.5-92.0)	93.4 (91.1-94.9)
**2013-2014**	88.1 (87.3-88.8)	91.5 (90.4-92.7)	90.3 (88.8-92.0)	93.5 (91.7-95.2)
**2014-2015**	88.3 (87.4-88.9)	91.6 (90.4-93.0)	90.9 (89.3-92.5)	93.7 (91.8-95.3)
**2015-2016**	87.7 (86.8-88.7)	92.2 (90.7-94.2)	92.0 (90.1-94.1)	94.7 (92.9-100)
**2016-2017**	88.2 (87.4-88.9)	91.8 (90.6-93.3)	91.7 (89.8-93.4)	94.4 (92.3-96.5)
**2017-2018**	88.2 (87.3-88.9)	91.7 (90.4-93.1)	91.6 (89.7-93.4)	94.4 (92.4-100)
**2018-2019**	88.2 (87.3-88.9)	91.9 (90.6-92.9)	91.8 (89.9-93.9)	94.5 (92.6-100)

For peer review only

Table 5: Medians (25th – 75th centiles) of ratio for users ≥50th centile over the rest of consultations by consultation type, all practices

Year	All consultations by all staff	All consultations by GPs	Face-to-face consultations by all staff	Face-to-face consultations by GPs
2000-2001	6.1 (5.7-6.4)	7.5 (6.5-8.9)	6.0 (5.5-6.7)	7.7 (6.3-9.5)
2001-2002	6.3 (5.9-6.7)	7.7 (6.6-9.1)	6.4 (5.8-7.1)	8.2 (6.6-9.6)
2002-2003	6.5 (6.0-6.9)	8.0 (6.9-9.7)	6.5 (6.0-7.4)	8.5 (6.8-9.8)
2003-2004	6.6 (6.2-7.1)	8.4 (7.3-10.1)	6.7 (6.2-7.6)	8.7 (7.2-10.1)
2004-2005	7.0 (6.6-7.6)	9.2 (7.6-12.0)	7.5 (6.6-9.7)	9.4 (7.7-11.4)
2005-2006	6.9 (6.5-7.4)	9.0 (7.8-10.7)	7.1 (6.4-8.2)	9.2 (7.6-10.6)
2006-2007	7.2 (6.6-7.8)	9.9 (8.0-12.6)	7.8 (6.8-10)	9.9 (8.0-12.1)
2007-2008	7.0 (6.6-7.5)	9.2 (7.9-10.8)	7.2 (6.5-8.5)	9.2 (7.7-10.7)
2008-2009	7.0 (6.6-7.5)	9.2 (8.1-11.0)	7.4 (6.6-8.6)	9.7 (8.2-11.4)
2009-2010	6.9 (6.5-7.4)	9.3 (8.0-11.0)	7.4 (6.6-8.6)	9.8 (8.0-11.2)
2010-2011	7.0 (6.6-7.5)	9.3 (8.0-11.0)	7.5 (6.6-8.6)	9.9 (8.3-11.4)
2011-2012	7.1 (6.6-7.6)	9.3 (8.1-10.7)	7.5 (6.8-8.7)	10.0 (8.4-11.4)
2012-2013	7.1 (6.7-7.6)	9.3 (8.2-10.6)	7.6 (6.8-8.8)	9.8 (8.3-11.4)
2013-2014	7.1 (6.6-7.6)	9.6 (8.5-11.0)	7.7 (7.0-8.9)	10.2 (8.7-11.9)
2014-2015	7.2 (6.7-7.7)	9.7 (8.6-11.3)	8.2 (7.3-9.5)	10.5 (8.9-12.1)
2015-2016	6.9 (6.4-7.5)	10.4 (8.6-14.4)	9.5 (7.7-11.9)	11.4 (9.6-14.2)
2016-2017	7.2 (6.6-7.7)	9.8 (8.7-12.1)	8.7 (7.6-10.5)	11.0 (9.3-13.0)
2017-2018	7.1 (6.6-7.7)	9.9 (8.7-11.8)	8.6 (7.6-10.8)	11.1 (9.2-12.9)
2018-2019	7.2 (6.6-7.8)	9.9 (8.8-11.8)	8.9 (7.8-11.2)	11.2 (9.6-13.3)

For peer review only

75th centile

Table 6 Medians (25th – 75th centiles) of 75th centile number of consultations by consultation type, all practices

Year	All consultations by all staff	All consultations by GPs	Face-to-face consultations by all staff	Face-to-face consultations by GPs
**2000-2001**	16 (14-18)	7 (6-10)	7 (6-9)	5 (4-6)
**2001-2002**	17.5 (15-20)	7 (6-9)	7 (6-9)	5 (4-6)
**2002-2003**	19 (16-22)	7 (6-9)	7 (6-9)	5 (4-6)
**2003-2004**	21 (17-24)	8 (6-10)	7 (6-9)	5 (4-6)
**2004-2005**	22 (18-26)	8 (7-10)	7 (6-9)	5 (4-6)
**2005-2006**	24 (19-28)	9 (7-11)	7 (6-10)	5 (4-6)
**2006-2007**	24 (20-29)	9 (7-11)	7 (6-9)	4 (4-6)
**2007-2008**	26 (21-30)	9 (7-11)	7 (6-9)	5 (4-6)
**2008-2009**	27 (23-31.5)	9 (8-11)	7 (6-9)	5 (4-6)
**2009-2010**	28 (24-33)	10 (8-12)	7 (6-9)	5 (4-6)
**2010-2011**	28 (24-33)	10 (8-12)	7 (6-9)	5 (4-6)
**2011-2012**	29 (25-34)	10 (9-12)	7 (6-9)	5 (4-6)
**2012-2013**	30 (26-35)	10 (9-12)	7 (6-9)	5 (4-6)
**2013-2014**	32 (27-37)	11 (9-13)	7 (6-9)	5 (4-6)
**2014-2015**	32 (28-37)	11 (10-13)	7 (6-9)	5 (4-6)
**2015-2016**	32 (28-37)	11 (9-13)	7 (6-8)	5 (4-5)
**2016-2017**	32 (28-38)	11 (9-13)	7 (6-8)	5 (4-6)
**2017-2018**	33 (29-38)	11 (9-13)	7 (6-8)	4 (3-5)
**2018-2019**	35 (30-40)	11 (10-13)	7 (6-8)	4 (3-5)

For peer review only

Table 7 Medians (25th – 75th centiles) of proportion of high volume (≥ 75th centile) by consultation type, all practices

Year	All consultations by all staff	All consultations by GPs	Face-to-face consultations by all staff	Face-to-face consultations by GPs
**2000-2001**	62.4 (61.2-63.9)	65.8 (63.5-69.1)	63.7 (61.6-66.0)	66.4 (63.8-69.9)
**2001-2002**	62.9 (61.6-64.3)	66.5 (64.0-69.1)	63.7 (61.8-66.4)	66.8 (64.3-70.4)
**2002-2003**	63.1 (61.9-64.6)	67.4 (64.7-69.8)	64.4 (62.3-66.7)	67.9 (64.3-71.3)
**2003-2004**	63.5 (62.2-64.8)	67.7 (65.4-70.2)	64.9 (62.7-67.3)	68.2 (65.0-71.8)
**2004-2005**	64.3 (63.0-65.7)	68.9 (66.1-71.7)	66.2 (63.9-69.0)	69.5 (66.1-73.4)
**2005-2006**	63.8 (62.6-65.3)	68.2 (65.9-70.8)	65.4 (63.2-67.7)	68.9 (65.7-72.2)
**2006-2007**	64.6 (63.0-66.0)	69.3 (66.3-71.9)	66.4 (64.0-69.4)	70.3 (66.7-73.9)
**2007-2008**	64.1 (62.7-65.4)	68.4 (66.4-70.7)	65.5 (63.2-68.0)	69.2 (66.0-72.8)
**2008-2009**	64.2 (62.7-65.5)	68.2 (66.5-70.7)	65.8 (63.9-68.6)	69.5 (66.4-72.7)
**2009-2010**	63.8 (62.6-65.2)	68.4 (66.2-70.5)	65.8 (63.7-68.0)	69.5 (66.6-72.4)
**2010-2011**	64.0 (62.7-65.5)	68.3 (66.4-70.4)	66.3 (64.2-68.5)	69.5 (66.8-72.4)
**2011-2012**	64.3 (62.9-65.4)	68.4 (66.5-70.2)	66.2 (64.1-68.8)	69.8 (67.0-73.0)
**2012-2013**	64.3 (62.9-65.6)	68.4 (66.8-70.2)	66.5 (64.6-68.8)	69.6 (67.0-73.1)
**2013-2014**	64.2 (62.9-65.7)	68.7 (66.8-70.5)	66.7 (64.6-69.0)	70.1 (67.4-73.4)
**2014-2015**	64.5 (62.9-65.9)	69.0 (67.3-70.8)	67.3 (65.3-69.4)	70.6 (67.9-73.8)
**2015-2016**	63.7 (62.4-65.5)	69.6 (67.4-72.5)	68.8 (66.4-71.7)	72.3 (69.1-76.8)
**2016-2017**	64.3 (62.8-65.8)	69.2 (67.3-71.2)	68.2 (66.0-70.7)	71.5 (68.3-75.6)
**2017-2018**	64.1 (62.7-65.8)	69.0 (67.1-71.3)	68.4 (65.5-71.4)	72.1 (69.0-76.1)
**2018-2019**	64.2 (62.6-65.5)	69.0 (67.3-71.3)	68.6 (66.2-71.2)	72.6 (69.3-77.2)

For peer review only

Table 8: Medians (25th – 75th centiles) of ratio for users ≥75th centile over the rest of consultations by consultation type, all practices

Year	All consultations by all staff	All consultations by GPs	Face-to-face consultations by all staff	Face-to-face consultations by GPs
2000-2001	4.7 (4.5-5.0)	5.1 (4.8-5.7)	4.6 (4.4-4.9)	5.0 (4.6-5.5)
2001-2002	4.8 (4.6-5.1)	5.3 (4.9-5.8)	4.7 (4.4-5.0)	5.1 (4.7-5.7)
2002-2003	4.9 (4.6-5.2)	5.4 (5.0-6.1)	4.8 (4.5-5.2)	5.1 (4.8-5.8)
2003-2004	5.0 (4.7-5.3)	5.7 (5.2-6.3)	4.9 (4.6-5.3)	5.3 (4.9-6.0)
2004-2005	5.2 (4.9-5.5)	6.0 (5.4-6.7)	5.2 (4.8-5.8)	5.7 (5.2-6.6)
2005-2006	5.1 (4.8-5.4)	5.8 (5.4-6.5)	5.0 (4.7-5.5)	5.5 (5.0-6.3)
2006-2007	5.3 (4.9-5.6)	6.2 (5.5-7.0)	5.3 (4.9-6.0)	5.9 (5.2-6.8)
2007-2008	5.2 (4.9-5.5)	5.9 (5.5-6.5)	5.1 (4.7-5.5)	5.6 (5.1-6.2)
2008-2009	5.2 (4.9-5.5)	5.9 (5.5-6.6)	5.1 (4.8-5.6)	5.6 (5.2-6.3)
2009-2010	5.1 (4.9-5.4)	6.0 (5.5-6.5)	5.1 (4.8-5.6)	5.6 (5.2-6.3)
2010-2011	5.2 (4.9-5.5)	6.0 (5.5-6.5)	5.2 (4.8-5.6)	5.6 (5.2-6.2)
2011-2012	5.2 (4.9-5.5)	6.0 (5.6-6.5)	5.2 (4.9-5.7)	5.7 (5.3-6.3)
2012-2013	5.2 (4.9-5.6)	6.0 (5.6-6.5)	5.3 (4.9-5.7)	5.8 (5.3-6.4)
2013-2014	5.2 (5.0-5.6)	6.1 (5.7-6.6)	5.3 (5.0-5.8)	5.9 (5.4-6.5)
2014-2015	5.3 (5.0-5.6)	6.2 (5.8-6.7)	5.4 (5.1-6.0)	6.0 (5.5-6.7)
2015-2016	5.2 (4.8-5.6)	6.4 (5.9-7.3)	5.9 (5.3-6.6)	6.6 (5.8-7.7)
2016-2017	5.3 (4.9-5.6)	6.3 (5.8-6.9)	5.7 (5.2-6.3)	6.3 (5.7-7.3)
2017-2018	5.2 (4.9-5.6)	6.3 (5.7-6.8)	5.7 (5.2-6.3)	6.4 (5.8-7.4)
2018-2019	5.2 (4.9-5.6)	6.3 (5.8-6.9)	5.7 (5.3-6.4)	6.5 (5.8-7.7)

For peer review only

Twelve or more consultations

Table 9 Medians (25th – 75th centiles) of proportion of patients with 12 or more consultations by consultation type, all practices

Year	All consultations by all staff	All consultations by GPs	Face-to-face consultations by all staff	Face-to-face consultations by GPs
**2000-2001**	36.0 (30.5-40.5)	12.6 (7.5-20.1)	10.6 (7.9-15.6)	4.9 (3.4-9.3)
**2001-2002**	38.9 (32.9-42.8)	12.5 (8.1-17.9)	11.2 (7.9-16.7)	4.6 (3.0-8.4)
**2002-2003**	40.9 (34.7-45.9)	12.7 (7.7-18.2)	10.7 (7.5-16.4)	4.3 (2.8-7.7)
**2003-2004**	43.3 (37.2-48.4)	14.4 (10.2-19.5)	10.8 (7.5-18.1)	4.2 (2.6-7.5)
**2004-2005**	44.3 (37.8-49.5)	15.7 (11.6-20.7)	11.2 (7.4-18.4)	4.1 (2.5-7.9)
**2005-2006**	47.2 (40.7-52.5)	17.0 (13.3-22.7)	11.9 (8.1-20.1)	4.3 (2.7-8.0)
**2006-2007**	47.3 (41.0-52.4)	17.5 (13.2-22.8)	11.6 (7.7-19.5)	4.2 (2.6-8.2)
**2007-2008**	49.5 (44.2-53.9)	18.0 (13.0-23.3)	11.8 (8.2-17.9)	4.2 (2.8-7.2)
**2008-2009**	51.1 (46.2-55.2)	19.0 (14.3-23.7)	11.7 (8.4-16.9)	4.4 (2.9-6.6)
**2009-2010**	52.8 (48.2-56.6)	20.0 (15.4-25.4)	12.2 (9.0-17.0)	4.7 (2.9-6.8)
**2010-2011**	52.7 (48.3-56.7)	20.7 (16.4-25.3)	12.1 (8.6-16.3)	4.7 (2.9-7.0)
**2011-2012**	53.8 (49.4-57.5)	21.8 (17.3-26.2)	12.2 (8.7-16.1)	4.7 (3.0-7.0)
**2012-2013**	54.5 (50.4-58.4)	22.3 (18.1-27.4)	12.4 (9.1-16.4)	4.6 (3.0-7.4)
**2013-2014**	55.6 (51.2-59.7)	23.8 (19.4-28.6)	12.5 (8.9-16.4)	4.6 (2.9-7.4)
**2014-2015**	56.0 (51.8-60.1)	24.1 (19.8-29.2)	12.3 (8.8-16.3)	4.6 (2.9-7.3)
**2015-2016**	56.2 (52.1-60.5)	23.0 (18.7-28.0)	11.7 (8.3-15.5)	4.5 (2.4-7.2)
**2016-2017**	56.4 (52.1-60.4)	24.0 (18.9-29.2)	11.8 (8.2-16.1)	4.5 (2.5-7.7)
**2017-2018**	56.9 (52.9-60.9)	24.0 (19.6-28.9)	11.4 (7.4-15.8)	4.2 (2.2-7.3)
**2018-2019**	58.4 (54.1-61.8)	24.7 (19.9-29.2)	11.3 (7.3-15.4)	4.3 (2.1-7.1)

For peer review only

Table 10 Medians (25th – 75th centiles) of proportion of high volume (12 or more consultations) by consultation type, all practices

Year	All consultations by all staff	All consultations by GPs	Face-to-face consultations by all staff	Face-to-face consultations by GPs
**2000-2001**	73.9 (68.3-78.5)	43.1 (30.6-56.4)	35.8 (28.1-47.0)	20.7 (15.7-34.1)
**2001-2002**	77.6 (71.5-81.3)	43.2 (31.7-53.7)	36.8 (28.6-48.1)	20.2 (15.1-31.7)
**2002-2003**	79.8 (74.1-83.8)	43.6 (31.1-54.8)	36.1 (28.3-49.3)	19.4 (14.0-29.8)
**2003-2004**	82.3 (76.5-85.9)	48.3 (37.5-57.2)	37.3 (28.4-52.7)	19.4 (13.8-31.0)
**2004-2005**	83.8 (78.2-87.0)	51.7 (42.4-60.2)	39.3 (29.7-55.8)	19.7 (13.6-33.3)
**2005-2006**	85.6 (80.5-88.8)	54.1 (45.7-62.5)	40.4 (30.7-57.0)	20.1 (14.4-33.4)
**2006-2007**	86.1 (81.5-89.1)	55.9 (46.9-64.2)	41.1 (31.8-56.0)	20.4 (14.8-33.2)
**2007-2008**	87.2 (83.3-89.8)	55.7 (46.2-64.0)	40.2 (31.4-52.6)	20.4 (14.8-30.6)
**2008-2009**	88.3 (84.9-90.6)	57.5 (48.5-64.5)	40.1 (32.0-51.0)	21.2 (15.1-28.9)
**2009-2010**	88.9 (86.2-91.1)	59.3 (50.6-66.6)	41.0 (33.2-51.0)	22.0 (15.5-29.5)
**2010-2011**	89.1 (86.4-91.3)	59.9 (52.3-66.7)	41.0 (32.7-50.1)	21.9 (15.7-30.1)
**2011-2012**	89.8 (87.3-91.7)	61.6 (54.1-68.4)	41.7 (33.0-49.5)	21.9 (16.0-30.0)
**2012-2013**	90.2 (87.8-92.1)	62.8 (56.1-70.3)	42.4 (33.4-50.0)	22.3 (16.1-31.3)
**2013-2014**	90.7 (88.3-92.6)	65.1 (58.4-71.4)	42.5 (33.8-50.5)	22.1 (15.8-32.1)
**2014-2015**	90.9 (88.7-92.8)	66.2 (59.6-72.0)	43.2 (34.0-50.5)	22.9 (16.1-32.4)
**2015-2016**	90.6 (88.3-92.5)	65.7 (58.4-71.6)	43.0 (33.7-50.8)	23.1 (15.2-32.4)
**2016-2017**	91.2 (88.9-93.0)	66.1 (58.7-72.7)	42.9 (33.5-51.5)	22.7 (14.5-34.0)
**2017-2018**	91.3 (89.3-93.2)	65.8 (59.2-72.2)	41.7 (32.0-51.5)	22.9 (14.0-32.5)
**2018-2019**	92.0 (90.2-93.6)	67.0 (59.9-73.0)	41.6 (31.5-50.5)	22.2 (13.4-32.0)

For peer review only

STROBE Statement—Checklist of items that should be included in reports of ***cohort studies***

	Item No	Recommendation	
**Title and abstract**	1	(*a*) Indicate the study's design with a commonly used term in the title or the abstract	p1
		(*b*) Provide in the abstract an informative and balanced summary of what was done and what was found	p3
**Introduction**			
Background/rationale	2	Explain the scientific background and rationale for the investigation being reported	p4
Objectives	3	State specific objectives, including any prespecified hypotheses	p5
**Methods**			
Study design	4	Present key elements of study design early in the paper	p5
Setting	5	Describe the setting, locations, and relevant dates, including periods of recruitment, exposure, follow-up, and data collection	p5
Participants	6	(*a*) Give the eligibility criteria, and the sources and methods of selection of participants. Describe methods of follow-up	p5
		(*b*) For matched studies, give matching criteria and number of exposed and unexposed	NA
Variables	7	Clearly define all outcomes, exposures, predictors, potential confounders, and effect modifiers. Give diagnostic criteria, if applicable	p5-6
Data sources/ measurement	8*	For each variable of interest, give sources of data and details of methods of assessment (measurement). Describe comparability of assessment methods if there is more than one group	p5-6
Bias	9	Describe any efforts to address potential sources of bias	p6
Study size	10	Explain how the study size was arrived at	p5
Quantitative variables	11	Explain how quantitative variables were handled in the analyses. If applicable, describe which groupings were chosen and why	p5-6
Statistical methods	12	(*a*) Describe all statistical methods, including those used to control for confounding	p6
		(*b*) Describe any methods used to examine subgroups and interactions	p6
		(*c*) Explain how missing data were addressed	NA
		(*d*) If applicable, explain how loss to follow-up was addressed	NA
		(*e*) Describe any sensitivity analyses	p6
**Results**			
Participants	13*	(a) Report numbers of individuals at each stage of study—eg numbers potentially eligible, examined for eligibility, confirmed eligible, included in the study, completing follow-up, and analysed	p7
		(b) Give reasons for non-participation at each stage	NA
		(c) Consider use of a flow diagram	NA
Descriptive data	14*	(a) Give characteristics of study participants (eg demographic, clinical, social) and information on exposures and potential confounders	NA
		(b) Indicate number of participants with missing data for each variable of interest	NA
		(c) Summarise follow-up time (eg, average and total amount)	NA
Outcome data	15*	Report numbers of outcome events or summary measures over time	p7
Main results	16	(*a*) Give unadjusted estimates and, if applicable, confounder-adjusted estimates and their precision (eg, 95% confidence interval). Make clear which confounders were adjusted for and why they were included	p7
		(*b*) Report category boundaries when continuous variables were categorized	NA
		(*c*) If relevant, consider translating estimates of relative risk into absolute risk for a meaningful time period	p7

For peer review only

Other analyses	17	Report other analyses done—eg analyses of subgroups and interactions, and sensitivity analyses	p8
**Discussion**			
Key results	18	Summarise key results with reference to study objectives	p8
Limitations	19	Discuss limitations of the study, taking into account sources of potential bias or imprecision. Discuss both direction and magnitude of any potential bias	p9-10
Interpretation	20	Give a cautious overall interpretation of results considering objectives, limitations, multiplicity of analyses, results from similar studies, and other relevant evidence	p10
Generalisability	21	Discuss the generalisability (external validity) of the study results	p10
**Other information**			
Funding	22	Give the source of funding and the role of the funders for the present study and, if applicable, for the original study on which the present article is based	p11

*Give information separately for exposed and unexposed groups.

Note: An Explanation and Elaboration article discusses each checklist item and gives methodological background and published examples of transparent reporting. The STROBE checklist is best used in conjunction with this article (freely available on the Web sites of PLoS Medicine at http://www.plosmedicine.org/, Annals of Internal Medicine at http://www.annals.org/, and Epidemiology at http://www.epidem.com/). Information on the STROBE Initiative is available at http://www.strobe-statement.org.

For peer review only

For peer review only - http://bmjopen.bmj.com/site/about/guidelines.xhtml

BMJ Open

Consultation patterns and frequent attenders in UK primary care from 2000 to 2019: a cohort analysis of consultation events across 845 general practices.

Journal:	*BMJ Open*
Manuscript ID	bmjopen-2021-054666.R1
Article Type:	Original research
Date Submitted by the Author:	02-Sep-2021
Complete List of Authors:	Kontopantelis, Evangelos; The University of Manchester, Division of Informatics, Imaging and Data Science; NIHR School for Primary Care Research, University of Oxford<br>Panagioti, Maria; The University of Manchester, Division of Population Health, Health Services Research & Primary Care; The University of Manchester, NIHR Greater Manchester Patient Safety Translational Research Centre<br>Farragher, Tracey; The University of Manchester, Division of Population Health, Health Services Research & Primary Care<br>Munford, Luke; The University of Manchester, Division of Population Health, Health Services Research & Primary Care<br>Parisi, Rosa; The University of Manchester, Division of Informatics, Imaging and Data Science<br>Planner, Claire; The University of Manchester, Division of Population Health, Health Services Research & Primary Care; NIHR School for Primary Care Research, University of Oxford<br>Spooner, Sharon; The University of Manchester, Health Organisation, Policy and Economics (HOPE) Group, Centre for Primary Care & Health Services Research; The University of Manchester, Division of Population Health, Health Services Research & Primary Care<br>Tse, Alice; The University of Manchester, Division of Population Health, Health Services Research & Primary Care<br>Ashcroft, Darren; The University of Manchester, NIHR Greater Manchester Patient Safety Translational Research Centre; The University of Manchester, Division of Pharmacy & Optometry<br>Esmail, Aneez; The University of Manchester, Division of Population Health, Health Services Research & Primary Care; NIHR School for Primary Care Research, University of Oxford
**Primary Subject Heading**:	General practice / Family practice
Secondary Subject Heading:	Health economics, Health services research, Medical management
Keywords:	PRIMARY CARE, Health policy < HEALTH SERVICES ADMINISTRATION & MANAGEMENT, Organisation of health services < HEALTH SERVICES ADMINISTRATION & MANAGEMENT

I, the Submitting Author has the right to grant and does grant on behalf of all authors of the Work (as defined in the below author licence), an exclusive licence and/or a non-exclusive licence for contributions from authors who are: i) UK Crown employees; ii) where BMJ has agreed a CC-BY licence shall apply, and/or iii) in accordance with the terms applicable for US Federal Government officers or employees acting as part of their official duties; on a worldwide, perpetual, irrevocable, royalty-free basis to BMJ Publishing Group Ltd ("BMJ") its licensees and where the relevant Journal is co-owned by BMJ to the co-owners of the Journal, to publish the Work in this journal and any other BMJ products and to exploit all rights, as set out in our licence.

The Submitting Author accepts and understands that any supply made under these terms is made by BMJ to the Submitting Author unless you are acting as an employee on behalf of your employer or a postgraduate student of an affiliated institution which is paying any applicable article publishing charge ("APC") for Open Access articles. Where the Submitting Author wishes to make the Work available on an Open Access basis (and intends to pay the relevant APC), the terms of reuse of such Open Access shall be governed by a Creative Commons licence – details of these licences and which Creative Commons licence will apply to this Work are set out in our licence referred to above.

Other than as permitted in any relevant BMJ Author's Self Archiving Policies, I confirm this Work has not been accepted for publication elsewhere, is not being considered for publication elsewhere and does not duplicate material already published. I confirm all authors consent to publication of this Work and authorise the granting of this licence.

For peer review only

Consultation patterns and frequent attenders in UK primary care from 2000 to 2019: a cohort analysis of consultation events across 845 general practices.

Evangelos Kontopantelis, Professor in Data Science and Health Services Research[1,2,3]
Maria Panagioti, Senior Lecturer[4,5]
Tracey Farragher, Lecturer in Healthcare Sciences[4]
Luke Munford, Lecturer in Health Economics[3,4]
Rosa Parisi, Research Fellow in Statistics[1,3]
Claire Planner, Research Associate[2,4]
Sharon Spooner, General Practitioner & Clinical Lecturer[2,3,4]
Alice Tse, General Practitioner[4]
Darren M. Ashcroft, Professor of Pharmacoepidemiology[2,5,6]
Aneez Esmail, Professor of General Practice[2,4]

1 Division of Informatics, Imaging and Data Science, University of Manchester, Manchester, England
2 NIHR School for Primary Care Research, University of Oxford, Oxford, England
3 Health Organisation, Policy and Economics (HOPE) research group; University of Manchester, Manchester, England
4 Division of Population Health, Health Services Research & Primary Care, University of Manchester, Manchester, England
5 NIHR Greater Manchester Patient Safety Translational Research Centre, Manchester, England
6 Division of Pharmacy & Optometry, University of Manchester, Manchester, England

Corresponding author:

Evangelos Kontopantelis, PhD
e.kontopantelis@manchester.ac.uk
Professor in Data Science and Health Services Research
Division of Informatics, Imaging and Data Sciences
University of Manchester
Oxford Road
M13 9PL
Manchester
UK

Keywords: primary care, consultations, workload, frequent attendance

Word count: 3,726
Abstract: 380

For peer review only - http://bmjopen.bmj.com/site/about/guidelines.xhtml

Abstract

Objective

To describe the distribution of consultations at the practice level and examine whether increases are uniform or driven by people who consult more frequently.

Design

Cohort study.

Setting

UK general practice data from CPRD GOLD.

Participants

1,699,709,314 consultation events from 12,330,545 patients, in 845 general practices (1/4/2000, 31/3/2019).

Methods

Consultation information was aggregated by financial year into: all consultations/all staff; all consultations/GPs; face-to-face consultations/all staff; face-to-face consultations/GPs. Patients with a number of consultations above the 90th centile, within each year, were classified as frequent attenders. Negative binomial regressions examined the association between available practice characteristics and consultation distribution.

Results

Among frequent attenders, all consultations by GPs increased from a median (25th, 75th centile) of 13 (10 to 16) to 21 (18 to 25) and all consultations by all staff increased from 27 (23, 30) to 60 (51, 69) over the study period. Approximately 4/10 consultations of any type concerned frequent attenders and the proportion of consultations attributed to them increased over time, particularly for face-to-face consultations with GPs, from a median of 38.0% (35.9%, 40.3%) in 2000/1 to 43.0% (40.6%, 46.4%) in 2018/9. Regression analyses indicated decreasing trends over time for face-to-face consultations and increasing trends for all consultation types, for both GPs and all staff. Frequent attenders consulted approximately 5 times more than the rest of the practice population, on average, with adjusted incidence rate ratios ranging between 4.992 (95%CI: 4.917, 5.068) for face-to-face consultations with all staff, and 5.603 (95%CI: 5.560, 5.647) for all consultations with GPs.

Conclusions

Frequent attenders progressively contributed to increased workload in general practices across the UK over the last 20 years. Important knowledge gaps remain in terms of the demographic, social and health characteristics of frequent attenders and how UK general practices can be prepared to meet the needs of these patients.

For peer review only

Article summary

Strengths and limitations of the study

- This is a large observational study that analysed 1,699,709,314 consultation events from 12,330,545 patients, in 845 general practices, from 1 April 2000 to 31 March 2019.
- The unique contribution of our study is the investigation of distribution trends amongst the top 10% of consulters for the following groups; all consultations (covering all staff working in general practice), all consultations with GPs (including face to face consultations and telephone consultations), face to face consultations with all staff and face to face consultations with GPs.
- This is an observational study, using a database of Electronic Health Records, and it is dependent on the consultations and consultation types being recorded accurately. We were not able to ascertain information on patient and practice characteristics which is critical to understanding why this group of frequent attending patients consult their GPs.
- Future work should examine persistent frequent attendance, and the patient and practice characteristics associated with frequent attendance.

For peer review only

Introduction

General practice in the United Kingdom (UK) has been facing significant workload challenges for more than a decade. In a seminal paper published in 2016, Hobbs and colleagues showed that the crude annual consultation rate per person increased by 10.51%, from 4.67 in 2007-08 to 5.16 in 2013-14.[1] Using data covering nearly 100 million consultations from anonymised practices contributing data to the Clinical Practice Research Datalink (CPRD GOLD), their study found that there was also a substantial increase in average consultation duration and total patient-facing clinical workload (by 16%) during this period. The authors concluded that the perceptions of a rapidly rising workload in UK general practice was well founded and compounded by the complexity of care that general practitioners (GPs) were having to provide including, for example, an increasing elderly population with multiple co-morbidities. The 2016 report by the King's Fund also confirmed that UK general practice is in crisis with a workload that has increased significantly over time. In addition to indicating that the increase was due to the needs of an ageing population and more people with complex conditions and multimorbidity, it was noted that the effect was compounded by initiatives to transfer care from hospitals to the community.[2]

Although existing work supports the view that GP workload is increasing over time,[1] up-to-date research evidence on workload rates is lacking. One important omission in previous estimates of UK GP workload is that the distribution of consultations within the patient population of general practices has not been assessed. In the Netherlands, Smits and colleagues identified a group of patients, referred to as persistent frequent attenders, occupying the top 10% of age and gender adjusted attenders over three continuous years. This group of frequent attenders may have special health needs, and may require different management in practice due to their distinct clinical and psychological characteristics.[3 4] As most of the literature on frequent attenders has been produced in the Netherlands, the generalisability of these findings and potential solutions in the UK needs to be evaluated. Pilot work in the UK supports these findings and could mean that the top 10% of attenders in the UK general practice are responsible for utilising between 30-50% of all GP consultations.[5] However, there is a paucity of large studies in the UK to establish firm estimates of the prevalence of frequent attendance in the GP workload.

Moreover, working arrangements within UK general practices change rapidly and dynamically. Major reforms are being implemented in response to the 2016 General Practice Forward View which could have an impact on workload rates.[6] For example, tele(e)-consultation options with GPs and other staff members of general practices have become increasingly prevalent, reaching a peak during the Covid-19 pandemic.[7 8] The workforce composition of general practices has also changed as more allied health professionals (e.g. advanced nurse practitioners, clinical pharmacists, and physician associates) and non-clinical staff have been appointed to increase the skill-mix in general practice and better serve the patient needs.[9 10] In addition, the health needs of local populations may affect consultation rates and workload demands differently in sub-regions within the UK .[11 12] Thus, potential variations in the GP workload across geographic regions need to be identified to tailor potential solutions.

This study provides new information about this under-researched topic by analysing the distribution of consultations within general practices and practice populations, with a focus on frequent attenders.

For peer review only - http://bmjopen.bmj.com/site/about/guidelines.xhtml

More specifically we examined: a) different ways to describe consultation distributions in a way that is informative to clinical practice, dichotomising into high and low attendance groups; b) how these vary across practices and regions, by consultation type; c) if and how they have changed over time. We also provide contemporary information about consultation rates to update the previous similar analysis undertaken five years ago.[1]

Methods

Data

We used the Clinical Practice Research Datalink (CPRD) GOLD database, which holds anonymised clinical information on patients, from participating general practices, including consultations, diagnoses, treatments, testing and referrals. Consultations include all contacts with the practice and will include requests for repeat prescriptions. They will also include consultations with practice staff (practice nurses, healthcare assistants, social prescribers, nurse practitioners and other allied health professional who are increasingly being employed by GPs). Some patients will contact a practice to speak to reception staff and these are usually identified as administrative episodes so will not be counted as consultations. Non face to face consultations will have included telephone and online consultations.

We analysed consultations data from 1 April 2000 until 31 March 2019, in annual bins of financial years. The number of practices in CPRD GOLD varied from 407 in 2000/1, to 740 in 2008/9, to 389 in 2018/19. Of these, only 113 practices contributed data throughout the whole of the study period. Although the database is representative of the UK in terms of age, gender and deprivation,[13] it is only broadly geographically representative since it collects data from practices using the Vision clinical system, and clinical system usage is geographically clustered in the UK.[14] For example, Scottish general practices are more heavily represented in more recent years of the CPRD database. Socio-economic deprivation of the practice location was available, as measured by the 2015 English Index of Multiple Deprivation (IMD) which is a composite score across seven domains: Income; Employment; Education; Skills and Training; Health and Disability; Crime; Barriers to Housing Services; Living Environment.[15] The Scottish, Welsh and Northern Irish IMDs are very similar, but with small domain changes to capture different country dynamics. Consultation information was aggregated within each financial year, for each active patient (registered for at least one day during the respective year). We aggregated the information into four consultation types: 1) all consultation types (including administrative) by all practice staff (health worker or administrator); 2) all consultation types by GPs; 3) face-to-face consultations by all staff; and 4) face-to-face consultations by all GPs (Supplementary file: Tables 1 & 2).

Statistical analyses

The distribution of consultations within each practice was quantified using various approaches, to allow for within and between practice comparisons over time. First, we calculated the mean, 10th, 25th, 50th (median), 75th and 90th centile of each of the four consultation types, for each practice within each financial year. Next we used the 50th, 75th and 90th centile to aggregate the consultation volume into dichotomous patient groups: those with a number of consultations in a given year below the 50th (or

For peer review only - http://bmjopen.bmj.com/site/about/guidelines.xhtml

75th or 90th) centile and those with a number of consultations equal to or higher than the 50th (or 75th or 90th) centile. We report the percentage of consultations attributed to the top group (equal to or above 50th, 75th or 90th centile), within each year. Finally, to allow for comparisons across practices, we used an arbitrary threshold of 12 consultations within a year to dichotomise volume, across each consultation type. As for centiles, we report the percentage of practice consultations attributed to people with 12 or more, within each year. The reported percentages, for centiles and 12 consultations, and for each practice within a year, can be expressed as:

$$p_{ij} = 100 * con_{ij1}/(con_{ij1} + con_{ij2})$$

Where:

$i = 1,\ldots,4$ the consultation type
j denotes the 50th, 75th, 90th centile or 12 consultations;
con_{ij1} is the total consultation volume for the top group;
con_{ij2} is the total consultation volume for the bottom group.

Since the groups were not always balanced as per the centiles (e.g. dichotomising along the 50th centile did not result in two equal groups of patients), due to the relative small numbers of consultations for the average patient, we also calculated the ratio of consultations of the top group over the bottom group. This can be expressed as:

$$r_{ij} = {}^{(con_{ij1}/pat_{ij1})}/{}_{(con_{ij2}/pat_{ij2})}$$

Where
pat_{ij1} is the number of patients in the top group;
pat_{ij2} is the number of patients in the bottom group.

We report these metrics in violin plots,[16] across all practices and by region, over time. Violin plots can be interpreted as box plots (including the median as a marker and a box indicating the interquartile range), overlaid with the density of the distribution for better visualisation.

We used negative binomial regression models for key outcomes of interest (50th, 75th, 90th centile and 12 consultations), to examine the association between available practice characteristics and imbalanced consultation distribution, with a focus on frequent attendance. The outcome was the average number of consultations per patient within each of the two groups, for each practice within a year, adjusted for the number of patients within each group (exposure term). The binary group predictor (top/bottom group) was interacted with each of the following practice characteristics, in separate models: list size (time varying), region, and practice location deprivation as measured by the 2015 Index of Multiple Deprivation.[15]

All analyses were conducted with Stata v16 and an alpha level of 5% was used for the inferential statistics. We focused on the 90th centile analyses in the main paper, splitting the population within a practice to the top 10% in terms of service usage within a year, and contrasting it to the remaining

For peer review only - http://bmjopen.bmj.com/site/about/guidelines.xhtml

90% of the practice population. As a sensitivity, we analysed data only from the 113 practices which were active throughout the whole of the study period.

Patient and Public Involvement

The research was discussed with a PPI group (PRIMER, based at the Centre for Primary Care and Health Service Research at the University of Manchester) at a meeting held prior to analysis. The Group agreed that understanding consultation patterns was an important starting point for exploring the reasons for frequent attendance (e.g. availability of double appointments for patients with multi-morbidity), the extent to which the needs of frequent attenders are being met and their experience of primary care services.

For peer review only

Results

Overall, 1,699,709,314 consultation events across 12,330,545 patients in 845 general practices were analysed. We present the 90th centile analyses in the main paper, and results were consistent for the other dichotomies (median, 75th centile and 12 or more consultations in a year), with summary results presented in the Supplementary file (Tables 3 to 10). Over time, the average volume of all consultations for a single patient, with GPs and all staff increased. However, face-to-face consultations remained relatively stable and face-to-face consultations with GPs even decreased (Table 1).

Over time, face-to-face consultations per annum with GPs and all staff remained stable for the top 10% group, and in 2018/19 medians (25th & 75th centiles) were 8 (6 & 10) and 12 (10 & 14), respectively (Table 2). On the other hand, medians for all consultations with GPs and all staff greatly increased over time. All consultations with GPs increased from a median (25th & 75th centiles) of 13 (10 & 16) in 2000/1 to 21 (18 & 25) in 2018/19. Similarly, all consultations with all staff increased from 27 (23 & 30) in 2000/1 to 60 (51 & 69) over the study period. However, the picture was somewhat different when quantifying service usage in relative terms, with increases over time across all consultations types for the top 10% group (Table 3 and Figure 1). The percentage of face-to-face consultations attributed to the top 10% of users within a year increased from a median (25th & 75th centiles) of 38.0% (35.9% & 40.3%) to 43.0% (40.6% & 46.4%) for interactions with GPs, and from 35.8% (34.4% & 37.7%) to 40.1% (38.3% & 42.5%) for interactions with all staff. Percentage increases for all consultations were smaller, from a median (25th & 75th centiles) of 38.0 (35.7% & 40.1%) to 40.9 (39.2% & 42.7%) with GPs, and from 35.2 (33.9% & 36.5%) to 36.6 (35.3% & 38.0%) with all staff, over the study period. These increases in service usage for the top 10% group, across all four outcomes, were also reflected in the ratio of consultations, with the largest increase observed in face-to-face consultations with GPs, from a median (25th & 75th centiles) of 4.7 (4.5 & 5.1) in 2000/1 to 5.9 (5.4 & 6.6) in 2018/19 (Table 4 and Figure 2).

There was relatively little regional variability in the median percentage attribution of all four consultation categories, across all examined patient dichotomies. For example, the median percentage of face-to-face consultations with GPs for the top 10% of patients in terms of service usage with their respective practice, varied from 35% for Wales and West Midlands to 38% for Northern Ireland, over the whole study period.

For peer review only - http://bmjopen.bmj.com/site/about/guidelines.xhtml

Results from the negative binomial regression analyses indicated decreasing trends over time for face-to-face consultations and increasing trends for all consultation types, for both GPs and all staff (Table 5 and Figure 3). Adjusted incidence rate ratios (IRRs) for the top 10% consultations group ranged between 4.99 (95% CI: 4.92 to 5.07) for face-to-face consultations with all staff, and 5.60 (95% CI: 5.56 to 5.65) for all consultations with GPs, compared to the bottom 90% consultations group. Associations between deprivation and consultations in the two groups were weak. Some regional variation was observed, with Scotland associated with the highest rate ratios for face-to-face consultations with GPs, and the South West associated with the highest rate ratios for face-to-face consultations with all staff. Pseudo-R^2 values for the four models varied between 0.048 for face-to-face consultations by all staff and 0.109 for all consultations by all staff.

Results from analyses of the other dichotomies (75-25, 50-50, 12 or more consultations) and sensitivity analyses focusing on practices for the whole of the study period were broadly similar to our main results.

Discussion

Summary of findings

This study examined distribution trends in four types of consultations (all consultations by GPs; all consultations by all staff; face-to-face consultations by GPs; face-to-face consultations by all staff) among frequent attenders (the top 10% of all consulters) and the rest of consulters in the UK general practices over the period from 2000/1 to 2018/9.

We found that all consultations by GPs and all consultations by all staff have considerably increased over this time period. Specifically, all consultations by GPs per person increased from a median of 5 to 8 and all consultations by all staff increased from 11 to 25. However, face-to-face consultations by GPs and face-to-face consultations by all staff have remained static (and may even have decreased).

Frequent attenders had five times more consultations of any type compared to the rest of consulters over the study period. Among frequent attenders, all consultations by GPs increased from a median of 13 to 21 and all consultations by all staff increased from 27 to 60 over the study period. Approximately 4 out of 10 consultations of any type concerned frequent attenders and the proportion of consultations attributed to frequent attenders increased over time, particularly for face-to-face consultations with GPs (from 38% in 2000/1 to 43% in 2018/9). We found little evidence of regional variability in the attribution of all four consultation types, across all categories of consulters. The only exemption was that the distribution of face-to-face consultations with GPs was highest in Scotland and the distribution of face-to-face consultations with all staff was highest in Northern Ireland.

Research in context

The unique contribution of our study is the investigation of distribution trends in all four types of consultations among frequent attenders (the top 10% of consulters). No previous study in the UK has

For peer review only - http://bmjopen.bmj.com/site/about/guidelines.xhtml

examined the contribution that frequent attenders make to the workload of general practices. Our findings showed that frequent attenders account for an increasing proportion of face-to-face consultations with GPs (38-43%) and are responsible for nearly 40% of all four types of consultations fairly constantly over time. This striking finding suggests that a relatively small number of patients are accounting for a large proportion of GP workload including face-to-face consultations. This could be potentially attributed to a relative reduction in accessibility to face-to-face consultations for non-frequent attenders, which may be driven by a perception of some or all actors (patient/carer/clinician/practice staff) that the need for face-to-face contact is smaller in this patient group. Despite the importance of these findings, there is very little research on frequent attenders in the UK to make direct comparisons. Outside the UK, our findings are consistent with epidemiological studies conducted in the Netherlands which has also shown that frequent attenders were responsible for nearly 40% of the face-to-face consultations.[3] Similarly, a previous review on frequent attenders reported that the top 10% of attenders accounted for 30% to 50% of all primary care consultations.[17]

Our current knowledge of the demographic, clinical and social characteristics of frequent attenders is scarce in the UK whereas findings from Europe are also inconsistent. Our analysis suggests that frequent attenders are identified across all parts of the UK and that deprivation, practice size or regional variation are not drivers for the number of frequent attenders by practice. There is evidence from Europe that frequent attenders (and particularly persistent frequent attenders) are more likely to be female and older, present more social and psychiatric problems, receive more prescriptions of psychotropic medication, have more medically unexplained physical symptoms, and more chronic medical conditions.[3 17]Our study cannot determine whether these factors are the main driving force for frequent attendance but the contribution to overall GP workload needs further examination. A systematic review focused on children also found that frequent attendance in children was associated with presence of psychosocial and mental health problems, younger age, school absence, presence of chronic conditions, and high level of anxiety in their parents.[18] However, another systematic review on older adults found that frequent attendance in late life was mainly associated with presence and severity of chronic medical conditions whereas associations with mental conditions, drug use and social support were heterogeneous.[19] Thus, although there is evidence that frequent attenders differ in terms of demographic, social, mental and physical characteristics from the rest of consulters, their profile may vary across age groups and according to different definitions of frequent attendance (e.g. persistent or no-persistent). Our findings and PPI work strongly support the need for future research to characterise frequent attenders in UK general practice taking into consideration the evidence and challenges encountered by previous studies conducted mostly in Europe.

Moreover, this study confirms and extends the secular trends first identified by Hobbs and colleagues that activity in general practice has increased over time [1]. This previous study found that the crude annual consultation rate per person increased from 4.67 in 2007-08, to 5.16 in 2013-14 and that GP telephone consultation rates doubled, compared with a 5.20% rise in face-to-face consultations. Consistent with this, we found that all consultations by GPs have increased and that face-to-face consultations are not the main driver of this increase. This finding suggests that other modalities for consulting such as telephone or online consultations may have become more important in recent years. Face-to-face consultations may have reached a capacity ceiling and other forms of consultations may be used to address otherwise unmet health needs. Moreover, Hobbs et al [1] found that the greatest increases in rates of consultations were in GPs, with a rise of 12.37% per 10,000 person-years,

For peer review only - http://bmjopen.bmj.com/site/about/guidelines.xhtml

compared with 0.9% for practice nurses. In our study however, the highest increase over time was observed in all consultations by all staff with consultation medians increasing from 11 in 2000/1 to 25 in 2018/9. This impressive increase in all consultations by all staff may suggest that the increase in multi-disciplinary staff working in general practices has resulted in a wider range of practice staff dealing with patients and that it is not all driven by face-to-face consultations. This trend appears to predate the 2016 GP Forward View and is likely to continue.

Limitations

This study has a number of important limitations. First, we used our own definition of frequent attenders (top 10% of consulters) and therefore our findings are only broadly comparable with other studies which have used other definitions (i.e. top 10% of consulters over 3 years; top 25% stratified by age, gender of health conditions). It is reassuring that our definition of frequent attendance is consistent with the recommended definition in a recent systematic review,[20] that our sensitivity analyses showed similar results, and that our findings were similar with the findings from our previous pilot study. Second, patients who have left a general practice but have not been removed from the register (also known as "ghost" patients) may exaggerate the differences between top and bottom users, and we know that their numbers vary by region.[21] This is an observational study, using a database of Electronic Health Records, and it is dependent on the consultations and consultation types being recorded accurately. Since the UK primary care has been almost fully computerised by the start of this century,[22] it is extremely unlikely that any consultations fail to be recorded. However, the default setting in recording a consultation is face-to-face, and this needs to be changed by the health professional for other consultation types. Thus, face-to-face consultations may be overestimated, if some health professionals miss that step. In addition, the underlying workload across consultation types may differ systematically, on top of expected patient variation, but this is something we cannot capture within this study of practice aggregates. Although the CPRD GOLD database is representative in terms of deprivation and population characteristics,[13] it is collecting data from a single computer system (VISION) and the contributing practices are not uniformly distributed across English regions, while its market share is in decline.[14] Thus, generalisability to every English region is questionable, especially in relation to the sensitivity analyses where the sample is quite small and the distribution of the practices across regional and deprivation strata changes. IMD scores are standardised within each UK country, and between country comparisons cannot capture baseline differences across countries. However, our regression analyses on the relative difference between high and low deprivation, in the context of the outcome of interest, should be robust to these nuances. Finally, we have not examined persistent frequent attendance, or the patient or practice characteristics associated with frequent attendance.

Implications for Policy and Practice

Frequent attenders appear to be a major driver for the increase in consultations that have contributed to perceptions of increased workload in general practice. Whilst many of these patients may have co-morbidities and may need to be seen regularly, research suggest that they have wider social and psychological needs.[17] GPs should be looking at this group of patients more closely to understand who are they and why are they consulting more frequently. Our PPI work indicates that patients see this as critically important for improving quality of care and preventing patients from being stigmatised for frequent attendance. One key challenge for future policy is how can general practice be prepared to

For peer review only - http://bmjopen.bmj.com/site/about/guidelines.xhtml

provide services and support to frequent attenders without medicalising them but addressing the wider social and psychological problems that they may have.

The large increase in the general practice workload the last 20 years means that having extended multidisciplinary teams to meet a wide range of patient needs through a range of ways (e.g. remote consultations) is perhaps the only solution for sustaining a viable primary care. This change in the workforce composition of general practices is reflected in the activities that we report (medians for all consultations by GPs increased 5 to 8 whereas all consultations by all staff increased from 11 to 25) and echoes recent policy documents such as the 2016 General Practice Forward View.[6] It is also likely that a multidisciplinary workforce in general practice could also better address the wider, not always medical, needs of frequent attenders.

Conclusions

This is the first study to show that frequent attenders, a relatively small group of consulters (the top 10%), largely and progressively contribute to increased workload in general practices across the UK over the last 20 years. Important knowledge gaps remain in terms of the demographic, social and health characteristics of frequent attenders in the UK and how general practices can be prepared to meet the needs of these patients. We intend to explore these factors in a separate study. Our findings depict a new model of work in general practice whereby an increasing number of consultations are conducted by other staff members (rather than GPs) using alternative means (rather than face-to-face consultations). With the overall workload of general practices on continuous rise, multidisciplinary and technology-enabled general practices could better meet the multiple and wider needs of frequent attenders and the rest of consulters.

For peer review only

Acknowledgements

We thank the members of the PRIMER patient and public involvement group (funded through the NIHR School for Primary Care Research) for their input into the early stages of this research and to Julie Billsborough, Manoj Mistry, Carole Bennett and Ailsa Donnelly for their contributions.

Ethical approval

This study is based on data from the Clinical Practice Research Datalink GOLD obtained under license from the Medicines and Healthcare Products Regulatory Agency (MHRA). The data are provided by patients and collected by the NHS as part of their care and support. The interpretation and conclusions contained in this study are those of the authors alone, and not necessarily those of the MHRA, the National Institute for Health Research (NIHR), National Health Service (NHS), or the Department of Health. Approval to conduct this study using the CPRD was granted by the Independent Scientific Advisory Committee (ISAC) of the Medicine and Healthcare Regulatory Agency (MHRA) (protocol 20_192R). We thank the contributing patients and practices to the CPRD GOLD who have allowed their data to be used for research purposes.

Funding

The University of Manchester. The funder had no role in any aspect of this work.

Data sharing statement

The data analysed in this study are subject to the following licenses/restrictions: Electronic health records are, by definition, considered "sensitive" data in the UK by the Data Protection Act and cannot be shared via public deposition because of information governance restriction in place to protect patient confidentiality. Access to data are available only once approval has been obtained through the individual constituent entity controlling access to the data. The primary care data can be requested via application to the Clinical Practice Research Datalink (www.cprd.com). Requests to access this dataset should be directed to enquiries@cprd.com.

Competing interests statement

All authors have completed the ICMJE uniform disclosure form and declare: support from the University of Manchester for the submitted work; no financial relationships with any organisations that might have an interest in the submitted work in the previous three years, no other relationships or activities that could appear to have influenced the submitted work.

Contributorship statement

The research idea originated with AE and AT, but all authors designed the study. EK extracted the data and conducted the analyses, LM and RP supported him in this. TF led the ISAC submission, for access to the CPRD GOLD database. EK, MP and AE drafted the manuscript, TF, LM, RP, CP, SS, AT and DA critically revised it.

For peer review only - http://bmjopen.bmj.com/site/about/guidelines.xhtml

Author Licence

I, the Submitting Author has the right to grant and does grant on behalf of all authors of the Work (as defined in the author licence), an exclusive licence and/or a non-exclusive licence for contributions from authors who are: i) UK Crown employees; ii) where BMJ has agreed a CC-BY licence shall apply, and/or iii) in accordance with the terms applicable for US Federal Government officers or employees acting as part of their official duties; on a worldwide, perpetual, irrevocable, royalty-free basis to BMJ Publishing Group Ltd ("BMJ") its licensees.

The Submitting Author accepts and understands that any supply made under these terms is made by BMJ to the Submitting Author unless you are acting as an employee on behalf of your employer or a postgraduate student of an affiliated institution which is paying any applicable article publishing charge ("APC") for Open Access articles. Where the Submitting Author wishes to make the Work available on an Open Access basis (and intends to pay the relevant APC), the terms of reuse of such Open Access shall be governed by a Creative Commons licence – details of these licences and which licence will apply to this Work are set out in our licence referred to above.

For peer review only

References

1. Hobbs FDR, Bankhead C, Mukhtar T, Stevens S, Perera-Salazar R, Holt T, et al. Clinical workload in UK primary care: a retrospective analysis of 100 million consultations in England, 2007-14. *Lancet* 2016;387:2323-30.
2. Baird B, Charles A, Honeyman M, Maguire D, Das P. *Understanding pressures in general practice*: King's Fund London, 2016.
3. Smits FT, Brouwer HJ, ter Riet G, van Weert HC. Epidemiology of frequent attenders: a 3-year historic cohort study comparing attendance, morbidity and prescriptions of one-year and persistent frequent attenders. *BMC Public Health* 2009;9:36.
4. Smits FT, Brouwer HJ, Zwinderman AH, Mohrs J, Smeets HM, Bosmans JE, et al. Morbidity and doctor characteristics only partly explain the substantial healthcare expenditures of frequent attenders: a record linkage study between patient data and reimbursements data. *BMC Fam Pract* 2013;14:138.
5. Patel S, Kai J, Atha C, Avery A, Guo B, James M, et al. Clinical characteristics of persistent frequent attenders in primary care: case-control study. *Fam Pract* 2015;32:624-30.
6. England N. General practice forward view. 2016.
7. Salisbury H. Helen Salisbury: Teleconsultations for all. *BMJ* 2020;370:m3211.
8. Salisbury C, Murphy M, Duncan P. The Impact of Digital-First Consultations on Workload in General Practice: Modeling Study. *J Med Internet Res* 2020;22:e18203.
9. Nelson P, Martindale AM, McBride A, Checkland K, Hodgson D. Skill-mix change and the general practice workforce challenge. *Br J Gen Pract* 2018;68:66-67.
10. Nelson PA, Bradley F, Martindale AM, McBride A, Hodgson D. Skill-mix change in general practice: a qualitative comparison of three 'new' non-medical roles in English primary care. *Br J Gen Pract* 2019;69:e489-e98.
11. Kontopantelis E, Mamas MA, van Marwijk H, Ryan AM, Bower P, Guthrie B, et al. Chronic morbidity, deprivation and primary medical care spending in England in 2015-16: a cross-sectional spatial analysis. *BMC Med* 2018;16:19.
12. Spooner S, Gibson J, Checkland K, McBride A, Hodgson DE, Hann M, et al. Regional variation in practitioner employment in general practices in England: a comparative analysis. *Br J Gen Pract* 2020;70:e164-e71.
13. Herrett E, Gallagher AM, Bhaskaran K, Forbes H, Mathur R, van Staa T, et al. Data Resource Profile: Clinical Practice Research Datalink (CPRD). *Int J Epidemiol* 2015;44:827-36.
14. Kontopantelis E, Stevens RJ, Helms PJ, Edwards D, Doran T, Ashcroft DM. Spatial distribution of clinical computer systems in primary care in England in 2016 and implications for primary care electronic medical record databases: a cross-sectional population study. *Bmj Open* 2018;8:e020738.
15. Kontopantelis E, Mamas MA, van Marwijk H, Ryan AM, Buchan IE, Ashcroft DM, et al. Geographical epidemiology of health and overall deprivation in England, its changes and persistence from 2004 to 2015: a longitudinal spatial population study. *J Epidemiol Community Health* 2018;72:140-47.
16. Winter N. VIOPLOT: Stata module to produce violin plots with current graphics. 2008.
17. Vedsted P, Christensen MB. Frequent attenders in general practice care: a literature review with special reference to methodological considerations. *Public Health* 2005;119:118-37.
18. Al-Saffar MN, Hayhoe BW, Harris MJ, Majeed A, Greenfield G. Children as frequent attenders in primary care: a systematic review. *BJGP Open* 2020;4.
19. Welzel FD, Stein J, Hajek A, Konig HH, Riedel-Heller SG. Frequent attenders in late life in primary care: a systematic review of European studies. *BMC Fam Pract* 2017;18:104.
20. Luciano JV, Fernandez A, Pinto-Meza A, Lujan L, Bellon JA, Garcia-Campayo J, et al. Frequent attendance in primary care: comparison and implications of different definitions. *Br J Gen Pract* 2010;60:49-55.

For peer review only - http://bmjopen.bmj.com/site/about/guidelines.xhtml

21. Burch P, Doran T, Kontopantelis E. Regional variation and predictors of over-registration in English primary care in 2014: a spatial analysis. *J Epidemiol Community Health* 2018;72:532-38.
22. Kontopantelis E, Buchan I, Reeves D, Checkland K, Doran T. Relationship between quality of care and choice of clinical computing system: retrospective analysis of family practice performance under the UK's quality and outcomes framework. *BMJ Open* 2013;3.

For peer review only

For peer review only - http://bmjopen.bmj.com/site/about/guidelines.xhtml

Tables

Table 1 Medians (25th – 75th centiles) of average number of consultations per patient, by consultation type, all practices

Year	All consultations by all staff	All consultations by GPs	Face-to-face consultations by all staff	Face-to-face consultations by GPs
**2000-2001**	11.4 (9.8-13.0)	5.3 (4.3-6.8)	5.2 (4.5-6.3)	3.7 (3.2-4.7)
**2001-2002**	12.5 (10.5-14.2)	5.3 (4.4-6.4)	5.2 (4.5-6.4)	3.6 (3.1-4.4)
**2002-2003**	13.4 (11.2-15.7)	5.3 (4.3-6.6)	5.1 (4.3-6.4)	3.5 (2.9-4.3)
**2003-2004**	14.8 (12.0-17.3)	5.7 (4.6-6.9)	5.1 (4.3-6.8)	3.4 (2.8-4.2)
**2004-2005**	15.8 (12.5-18.3)	5.9 (4.9-7.1)	5.0 (4.1-6.7)	3.2 (2.7-4.2)
**2005-2006**	17.0 (13.8-20.1)	6.3 (5.4-7.6)	5.3 (4.4-7.0)	3.3 (2.8-4.3)
**2006-2007**	17.3 (14.0-20.5)	6.3 (5.3-7.7)	5.1 (4.2-6.9)	3.2 (2.7-4.1)
**2007-2008**	18.4 (15.3-21.2)	6.5 (5.4-7.7)	5.2 (4.3-6.6)	3.3 (2.8-4.1)
**2008-2009**	19.4 (16.5-22.4)	6.8 (5.6-7.9)	5.2 (4.4-6.4)	3.3 (2.8-3.9)
**2009-2010**	20.3 (17.7-23.3)	7.1 (5.9-8.4)	5.3 (4.5-6.4)	3.4 (2.8-4.0)
**2010-2011**	20.4 (17.8-23.8)	7.3 (6.1-8.4)	5.3 (4.4-6.3)	3.4 (2.8-4.0)
**2011-2012**	21.3 (18.5-24.6)	7.6 (6.4-8.8)	5.4 (4.5-6.4)	3.4 (2.8-4.0)
**2012-2013**	21.8 (19.0-25.2)	7.6 (6.6-9.1)	5.4 (4.6-6.4)	3.4 (2.9-4.1)
**2013-2014**	22.8 (19.7-26.3)	8.0 (6.9-9.5)	5.4 (4.6-6.4)	3.4 (2.8-4.1)
**2014-2015**	23.4 (20.3-26.7)	8.2 (6.9-9.7)	5.4 (4.6-6.3)	3.3 (2.8-4.1)
**2015-2016**	23.2 (20.0-26.8)	7.9 (6.6-9.3)	5.1 (4.3-6.1)	3.2 (2.6-3.9)
**2016-2017**	23.4 (20.3-27.4)	8.3 (6.8-9.7)	5.2 (4.3-6.2)	3.3 (2.6-4.0)
**2017-2018**	23.8 (20.7-27.4)	8.2 (7.0-9.7)	5.1 (4.1-6.1)	3.2 (2.5-4.0)
**2018-2019**	25.1 (21.7-28.7)	8.3 (7.0-9.7)	5.0 (4.2-6.0)	3.1 (2.5-3.9)

For peer review only

For peer review only - http://bmjopen.bmj.com/site/about/guidelines.xhtml

Table 2 Medians (25th – 75th centiles) of 90th centile number of consultations per patient, by consultation type, all practices

Year	All consultations by all staff	All consultations by GPs	Face-to-face consultations by all staff	Face-to-face consultations by GPs
**2000-2001**	27 (23-30)	13 (10-16)	12 (10-15)	9 (8-11)
**2001-2002**	30 (25-34)	13 (10-16)	12 (10-15)	8 (7-11)
**2002-2003**	32 (26-37)	13 (10-16)	12 (10-15)	8 (7-10)
**2003-2004**	35 (29-41)	14 (12-17)	12 (10-16)	8 (7-10)
**2004-2005**	38 (30-45)	15 (12-18)	12 (10-16)	8 (7-10)
**2005-2006**	41 (33-49)	16 (13-19)	12 (10-17)	8 (7-10)
**2006-2007**	42 (34-50)	16 (13-19)	12 (10-16)	8 (7-10)
**2007-2008**	44 (37-52)	16 (13-19)	12 (10-15)	8 (7-10)
**2008-2009**	47 (40-54)	17 (14-20)	12 (10-15)	8 (7-10)
**2009-2010**	49 (42-56)	18 (15-21)	12 (11-15)	8 (7-10)
**2010-2011**	49 (43-57)	18 (15-21)	12 (10-15)	8 (7-10)
**2011-2012**	51 (44-58)	19 (16-22)	13 (11-15)	8 (7-10)
**2012-2013**	52 (45-60)	19 (16-23)	13 (11-15)	8 (7-10)
**2013-2014**	55 (47-63)	20 (17-24)	13 (11-15)	8 (7-10)
**2014-2015**	56 (49-64)	20 (17-24)	13 (11-15)	8 (7-10)
**2015-2016**	55 (48-64)	20 (17-23)	13 (11-15)	8 (7-10)
**2016-2017**	56.5 (48-66)	21 (17-24)	12.5 (10-15)	8 (7-10)
**2017-2018**	57 (50-66)	20 (17-24)	12 (10-15)	8 (6-10)
**2018-2019**	60 (51-69)	21 (18-25)	12 (10-14)	8 (6-10)

For peer review only

Table 3 Medians (25th – 75th centiles) of proportion of high volume (≥ 90th centile) per patient, by consultation type, all practices

Year	All consultations by all staff	All consultations by GPs	Face-to-face consultations by all staff	Face-to-face consultations by GPs
**2000-2001**	35.2 (33.9-36.5)	38.0 (35.7-40.1)	35.8 (34.4-37.7)	38.0 (35.9-40.3)
**2001-2002**	35.5 (34.1-36.8)	38.4 (36.5-40.4)	36.3 (34.6-38.2)	38.5 (36.5-40.9)
**2002-2003**	35.6 (34.3-37.0)	38.7 (36.6-41.1)	36.5 (35.0-38.4)	38.5 (36.4-41.2)
**2003-2004**	35.9 (34.5-37.2)	39.1 (37.2-41.5)	36.9 (35.2-39.0)	39.0 (36.7-41.7)
**2004-2005**	36.5 (35.0-37.9)	40.1 (37.8-42.4)	37.9 (35.9-40.3)	40.1 (37.6-43.3)
**2005-2006**	36.1 (34.9-37.6)	39.8 (37.6-41.9)	37.5 (35.7-39.7)	39.8 (37.3-42.5)
**2006-2007**	36.7 (35.2-38.1)	40.5 (38.2-42.8)	38.3 (36.3-40.7)	40.7 (38.2-44.2)
**2007-2008**	36.5 (35.1-37.9)	40.0 (38.2-42.1)	37.9 (36.1-40.0)	39.9 (37.9-42.9)
**2008-2009**	36.6 (35.3-37.9)	40.2 (38.3-42.1)	38.1 (36.5-40.0)	40.1 (37.8-43.1)
**2009-2010**	36.3 (35.1-37.7)	40.0 (38.4-41.9)	38.0 (36.3-40.0)	40.3 (38.1-43.0)
**2010-2011**	36.7 (35.3-38.0)	40.1 (38.5-42.0)	38.3 (36.6-40.5)	40.6 (38.3-43.1)
**2011-2012**	36.9 (35.5-38.1)	40.4 (38.7-42.1)	38.6 (37.0-40.6)	40.9 (38.6-43.5)
**2012-2013**	36.9 (35.5-38.2)	40.3 (38.7-41.9)	38.6 (37.1-40.5)	40.9 (38.7-43.3)
**2013-2014**	36.8 (35.5-38.3)	40.5 (39.1-42.2)	38.9 (37.2-40.7)	41.3 (39.4-43.6)
**2014-2015**	37.1 (35.6-38.5)	40.8 (39.3-42.5)	39.4 (37.5-41.4)	41.5 (39.3-44.0)
**2015-2016**	36.6 (35.3-38.2)	41.4 (39.7-43.7)	40.4 (38.4-42.7)	43.1 (40.4-46.3)
**2016-2017**	36.8 (35.6-38.4)	41.0 (39.6-43.0)	40.1 (38.1-42.4)	42.5 (40.3-45.4)
**2017-2018**	36.8 (35.3-38.3)	40.9 (39.1-42.9)	40.1 (38.1-42.2)	42.8 (40.1-45.4)
**2018-2019**	36.6 (35.3-38.0)	40.9 (39.2-42.7)	40.1 (38.3-42.5)	43.0 (40.6-46.4)

For peer review only

Table 4: Medians (25th – 75th centiles) of ratio for users ≥90th centile over the rest of consultations per patient, by consultation type, all practices

Year	All consultations by all staff	All consultations by GPs	Face-to-face consultations by all staff	Face-to-face consultations by GPs
**2000-2001**	4.6 (4.4-4.9)	4.9 (4.6-5.4)	4.5 (4.3-4.8)	4.7 (4.5-5.1)
**2001-2002**	4.7 (4.5-5.0)	5.0 (4.7-5.5)	4.6 (4.4-4.9)	4.8 (4.5-5.2)
**2002-2003**	4.7 (4.5-5.1)	5.2 (4.8-5.6)	4.7 (4.4-5.0)	4.9 (4.6-5.3)
**2003-2004**	4.8 (4.6-5.1)	5.3 (5.0-5.8)	4.8 (4.5-5.1)	5.0 (4.7-5.5)
**2004-2005**	5.0 (4.7-5.3)	5.5 (5.1-6.1)	5.0 (4.6-5.4)	5.2 (4.8-5.8)
**2005-2006**	4.9 (4.6-5.2)	5.5 (5.1-6.0)	4.8 (4.6-5.3)	5.1 (4.8-5.7)
**2006-2007**	5.0 (4.7-5.4)	5.7 (5.2-6.3)	5.1 (4.7-5.6)	5.4 (4.9-6.0)
**2007-2008**	5.0 (4.7-5.3)	5.6 (5.1-6.0)	5.0 (4.7-5.4)	5.2 (4.8-5.7)
**2008-2009**	5.0 (4.7-5.4)	5.6 (5.2-6.0)	5.0 (4.7-5.4)	5.3 (4.9-5.7)
**2009-2010**	5.0 (4.7-5.3)	5.6 (5.2-6.0)	5.0 (4.7-5.4)	5.2 (4.9-5.7)
**2010-2011**	5.1 (4.8-5.4)	5.7 (5.3-6.1)	5.1 (4.7-5.5)	5.3 (4.9-5.8)
**2011-2012**	5.1 (4.8-5.4)	5.7 (5.3-6.1)	5.1 (4.8-5.6)	5.4 (5.0-5.8)
**2012-2013**	5.1 (4.8-5.4)	5.7 (5.4-6.1)	5.2 (4.8-5.6)	5.4 (5.0-5.8)
**2013-2014**	5.1 (4.8-5.5)	5.8 (5.4-6.2)	5.2 (4.9-5.6)	5.5 (5.1-6.0)
**2014-2015**	5.2 (4.9-5.5)	5.9 (5.5-6.3)	5.3 (5.0-5.8)	5.6 (5.2-6.1)
**2015-2016**	5.1 (4.8-5.5)	6.0 (5.6-6.5)	5.6 (5.1-6.2)	5.9 (5.4-6.6)
**2016-2017**	5.1 (4.9-5.5)	5.9 (5.5-6.4)	5.5 (5.1-6.0)	5.8 (5.3-6.4)
**2017-2018**	5.1 (4.8-5.5)	5.9 (5.5-6.3)	5.5 (5.1-6.0)	5.8 (5.4-6.5)
**2018-2019**	5.1 (4.8-5.4)	5.9 (5.5-6.3)	5.5 (5.1-6.0)	5.9 (5.4-6.6)

For peer review only

Table 5: Associations between covariates of interest and consultation volume, incidence rate ratios (and 95% CIs) from main negative binomial regression model

	All consultations – all staff	All consultations – GPs	Face-to-face consultations – all staff	Face-to-face consultations - GPs
Year	1.046 (1.045,1.047)	1.028 (1.028,1.029)	0.995 (0.994,0.997)	0.988 (0.986,0.989)
List size (per 1000 patients)	1.003 (1.002,1.004)	0.997 (0.996,0.998)	1.001 (0.998,1.003)	0.992 (0.990,0.995)
Consultation group				
Bottom 90%	Reference category			
Top 10%	5.038 (5.007,5.068)	5.603 (5.560,5.647)	4.992 (4.917,5.068)	5.253 (5.171,5.337)
IMD Deprivation quintile				
(least deprived) 1	Reference category			
2	1.005 (0.994,1.016)	1.019 (1.005,1.033)	0.993 (0.967,1.020)	1.044 (1.015,1.073)
3	1.048 (1.037,1.059)	1.049 (1.035,1.063)	1.038 (1.011,1.065)	1.076 (1.047,1.106)
4	1.019 (1.009,1.030)	1.046 (1.032,1.060)	0.981 (0.956,1.006)	1.058 (1.030,1.087)
(most deprived) 5	1.025 (1.015,1.036)	0.993 (0.980,1.006)	0.997 (0.972,1.023)	1.007 (0.981,1.035)
Region				
North East	Reference category			
North West	0.926 (0.899,0.953)	0.995 (0.958,1.034)	1.033 (0.961,1.111)	0.985 (0.913,1.063)
Yorkshire & Humber	0.952 (0.920,0.985)	1.028 (0.984,1.075)	1.023 (0.940,1.113)	1.003 (0.918,1.096)
East Midlands	0.898 (0.867,0.930)	0.912 (0.872,0.955)	0.963 (0.883,1.050)	0.915 (0.835,1.002)
West Midlands	0.840 (0.815,0.865)	1.034 (0.994,1.074)	1.074 (0.998,1.157)	1.065 (0.986,1.150)
East of England	0.853 (0.827,0.880)	0.946 (0.909,0.985)	1.012 (0.937,1.092)	0.960 (0.886,1.040)
South West	0.954 (0.926,0.983)	1.085 (1.043,1.128)	1.256 (1.166,1.353)	1.080 (0.999,1.168)
South Central	0.844 (0.819,0.870)	0.952 (0.916,0.990)	1.100 (1.021,1.186)	1.052 (0.972,1.138)
London	0.738 (0.716,0.760)	0.922 (0.887,0.958)	0.895 (0.832,0.964)	0.918 (0.850,0.992)
South East Coast	0.830 (0.805,0.855)	0.928 (0.893,0.964)	0.987 (0.918,1.063)	1.000 (0.926,1.080)
Northern Ireland	1.150 (1.115,1.187)	1.446 (1.389,1.506)	1.087 (1.006,1.174)	1.128 (1.040,1.223)
Scotland	0.785 (0.763,0.808)	1.042 (1.004,1.081)	1.249 (1.163,1.340)	1.207 (1.121,1.300)
Wales	0.883 (0.858,0.909)	0.969 (0.934,1.006)	1.045 (0.973,1.123)	1.007 (0.934,1.086)

For peer review only - http://bmjopen.bmj.com/site/about/guidelines.xhtml

Figures

Figure 1: Percentage of consultations by people consulting at the 90th centile or above within a financial year, over time

Figure 2: Ratio of consultations for people ≥90th centile vs those below within a financial year, over time

Figure 3: Consultation count estimates by group over time (Predicted incidence rates from the negative binomial regression model with a time-group interaction term)*

* Interpreted as the estimated average number of consultations for a patient within each group

For peer review only

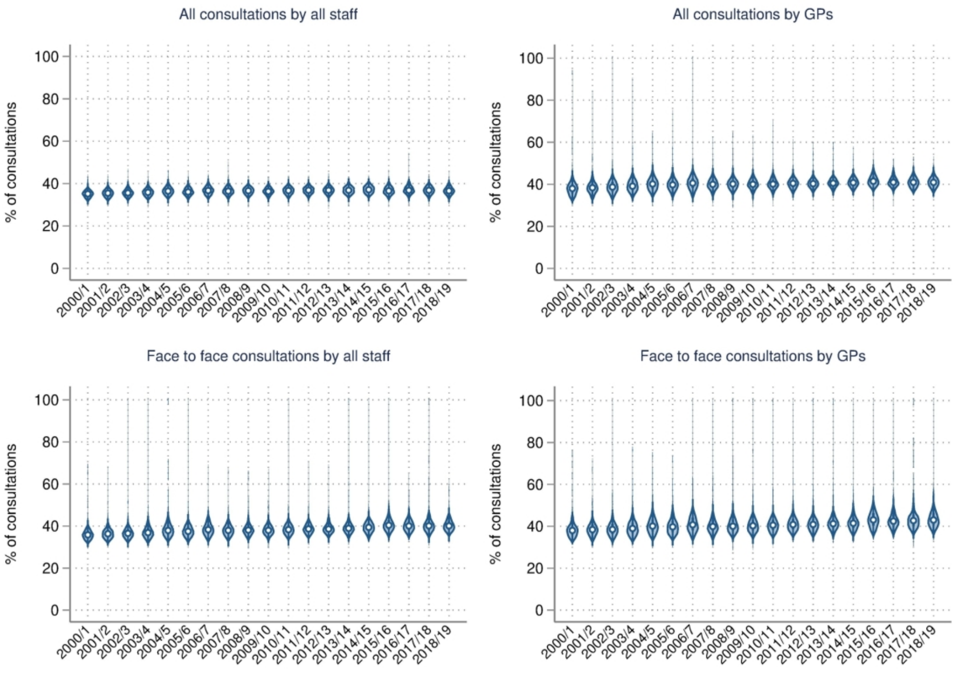

Figure 1: Percentage of consultations by people consulting at the 90th centile or above within a financial year, over time

177x127mm (300 x 300 DPI)

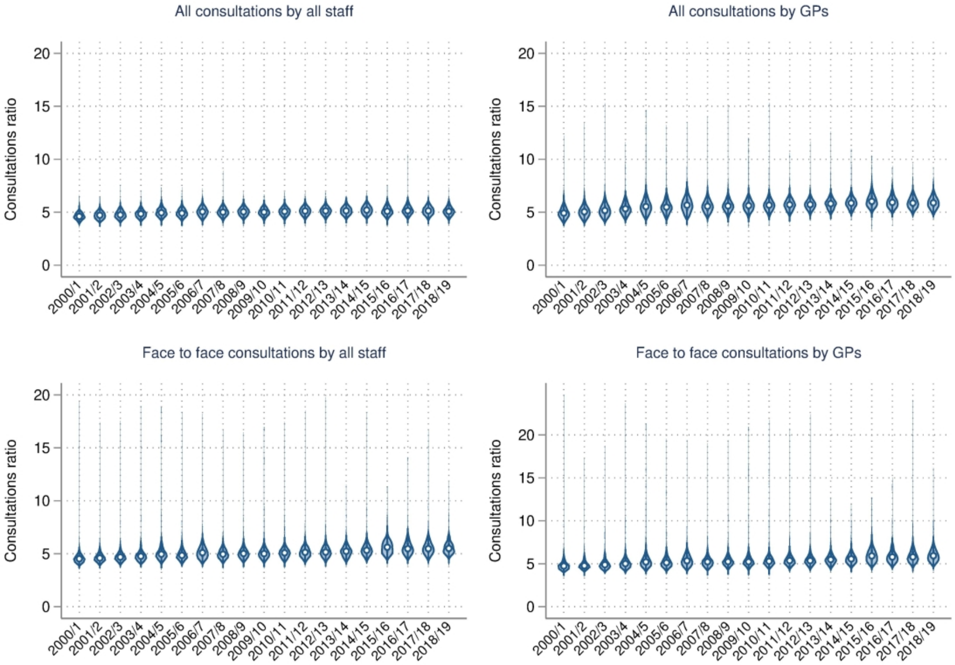

Figure 2: Ratio of consultations for people ≥90th centile vs those below within a financial year, over time

177x127mm (600 x 600 DPI)

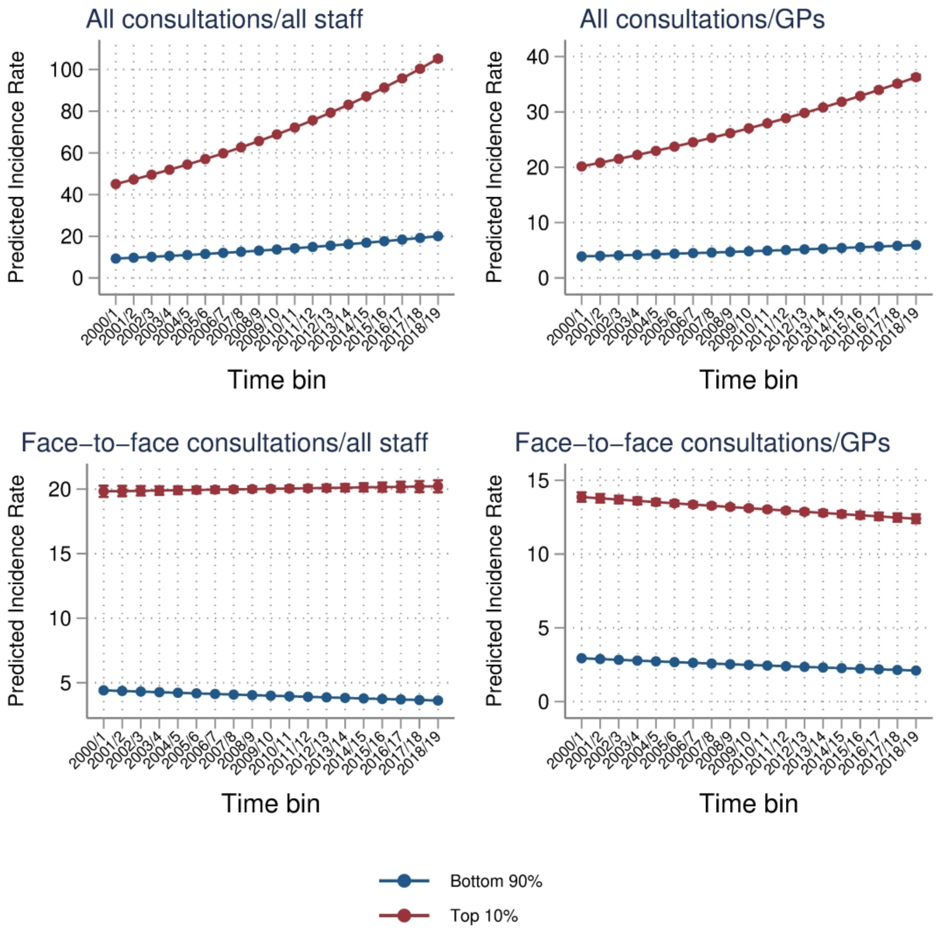

Figure 3: Consultation count estimates by group over time (Predicted incidence rates from the negative binomial regression model with a time-group interaction term)

203x203mm (600 x 600 DPI)

Supplementary file to "Consultation patterns and frequent attenders in UK primary care from 2000 to 2019: a retrospective analysis of 1,699,709,314 consultation events across 12,330,545 patients in 845 general practices"

Defining the consultation groups

GPs defined as: 1,2,3,4,5,6,7,8,9,10,47,50 in Table 1 (in red font).

Table 1: Staff Role

Code	Description
0	Data Not Entered
1	Senior Partner
2	Partner
3	Assistant
4	Associate
5	Non-commercial local rota of less than 10 GPs
6	Commercial Deputising service
7	Locum
8	GP Registrar
9	Consultant
10	Sole Practitioner
11	Practice Nurse
12	Health Visitor
13	Community Nurse
14	Midwife
15	Community Psychiatric Nurse
16	Social Worker
17	Pharmacist
18	Dispenser
19	Non-qualified Dispenser
20	Practice Manager
21	Fund Manager
22	Business Manager
23	Administrator
24	Secretary
25	Receptionist
26	Physiotherapist
27	Chiropodist
28	Dentist
29	Dietician
30	Counsellor
31	Osteopath
32	Maintenance staff
33	Other Health Care Professional
34	Hospital Nurse

For peer review only

Code	Description
35	Community Medical Officer
36	School Nurse
37	Health Education Officer
38	Contact Tracing Nurse
39	Stomatherapist
40	Computer Manager
41	Interpreter/Link Worker
42	Chiropractor
43	Acupuncturist
44	Homeopath
45	Mental Handicap Nurse
46	Carer
47	Salaried Partner
48	Occupational Therapist
49	Speech Therapist
50	GP Retainer
51	Phlebotomist
52	Other Medical & Dental
53	Other Students
54	Other Nursing & Midwifery
55	Other Allied Health Professionals
56	Other Professional Scientific & Technical
57	Other Healthcare Scientists
58	Other Additional Clinical Services
59	Other Admin & Clerical
60	Clinical Practitioner Access Role
61	Nurse Access Role
62	Nurse Manager Access Role
63	Health Professional Access Role
64	Healthcare Student Access Role
65	Biomedical Scientist Access Role
66	Clinical Coder Access Role
67	Optometrist
68	Radiographer

Face-to-face consultations defined as 1,2,3,4,6,7,8,9,11,18,24,27,28,30,31,32,33,34,36,37,38,50,61 in Table 2 (in red font).

Table 2: Consultation type

Code	Description
0	Data Not Entered
1	Clinic
2	Night visit, Deputising service
3	Follow-up/routine visit
4	Night visit, Local rota
5	Mail from patient
6	Night visit , practice

For peer review only - http://bmjopen.bmj.com/site/about/guidelines.xhtml

Code	Description
7	Out of hours, Practice
8	Out of hours, Non Practice
9	Surgery consultation
10	Telephone call from a patient
11	Acute visit
12	Discharge details
13	Letter from Outpatients
14	Repeat Issue
15	Other
16	Results recording
17	Mail to patient
18	Emergency Consultation
19	Administration
20	Casualty Attendance
21	Telephone call to a patient
22	Third Party Consultation
23	Hospital Admission
24	Children's Home Visit
25	Day Case Report
26	GOS18 Report
27	Home Visit
28	Hotel Visit
29	NHS Direct Report
30	Nursing Home Visit
31	Residential Home Visit
32	Twilight Visit
33	Triage
34	Walk-in Centre
35	Co-op Telephone advice
36	Co-op Surgery Consultation
37	Co-op Home Visit
38	Minor Injury Service
39	Medicine Management
40	Community Clinic
41	Community Nursing Note
42	Community Nursing Report
43	Data Transferred from other system
44	Health Authority Entry
45	Health Visitor Note
46	Health Visitor Report
47	Hospital Inpatient Report
48	Initial Post Discharge Review
49	Laboratory Request
50	Night Visit
51	Radiology Request
52	Radiology Result
53	Referral Letter

For peer review only

For peer review only - http://bmjopen.bmj.com/site/about/guidelines.xhtml

Code	Description
54	Social Services Report
55	Telephone Consultation
56	Template Entry
57	GP to GP communication transaction
58	Non-consultation medication data
59	Non-consultation data
60	ePharmacy message
61	Extended Hours

For peer review only

For peer review only - http://bmjopen.bmj.com/site/about/guidelines.xhtml

Median (50th centile)

Table 3 Medians (25th – 75th centiles) of median number of consultations by consultation type, all practices

Year	All consultations by all staff	All consultations by GPs	Face-to-face consultations by all staff	Face-to-face consultations by GPs
**2000-2001**	7 (6-8)	3 (3-4)	4 (3-4)	2 (2-3)
**2001-2002**	8 (7-9)	3 (3-4)	4 (3-4)	2 (2-3)
**2002-2003**	9 (7-10)	3 (3-4)	3 (3-4)	2 (2-3)
**2003-2004**	9 (7-11)	3 (3-4)	3 (3-4)	2 (2-3)
**2004-2005**	9 (7-11)	3 (3-4)	3 (3-4)	2 (2-3)
**2005-2006**	10 (8-13)	4 (3-4)	4 (3-4)	2 (2-3)
**2006-2007**	10 (8-13)	4 (3-4)	3 (3-4)	2 (2-3)
**2007-2008**	11 (9-13)	4 (3-5)	3 (3-4)	2 (2-3)
**2008-2009**	12 (10-14)	4 (3-5)	3 (3-4)	2 (2-3)
**2009-2010**	13 (11-15)	4 (3-5)	4 (3-4)	2 (2-3)
**2010-2011**	13 (11-15)	4 (3-5)	3 (3-4)	2 (2-3)
**2011-2012**	13 (11-15)	4 (4-5)	3 (3-4)	2 (2-3)
**2012-2013**	14 (12-16)	4 (4-5)	4 (3-4)	2 (2-3)
**2013-2014**	14 (12-17)	5 (4-5)	4 (3-4)	2 (2-3)
**2014-2015**	14 (12-17)	5 (4-6)	3 (3-4)	2 (2-3)
**2015-2016**	14 (12-17)	4 (3-5)	3 (3-4)	2 (1-2)
**2016-2017**	15 (12-17)	5 (4-6)	3 (3-4)	2 (1-2)
**2017-2018**	15 (13-18)	5 (4-6)	3 (3-4)	2 (1-2)
**2018-2019**	16 (13-18)	5 (4-6)	3 (2-4)	2 (1-2)

For peer review only

Table 4 Medians (25th – 75th centiles) of proportion of high volume (≥ 50th centile) by consultation type, all practices

Year	All consultations by all staff	All consultations by GPs	Face-to-face consultations by all staff	Face-to-face consultations by GPs
**2000-2001**	87.0 (86.2-87.8)	90.2 (88.2-92.4)	87.9 (86.0-90.1)	91.6 (87.9-94.0)
**2001-2002**	87.3 (86.4-88.1)	90.3 (88.4-92.5)	88.4 (86.8-90.4)	92.2 (88.7-94.0)
**2002-2003**	87.4 (86.6-88.2)	90.8 (89.1-93.0)	88.8 (87.2-90.5)	92.7 (89.2-94.2)
**2003-2004**	87.6 (86.9-88.4)	90.9 (89.3-92.8)	89.1 (87.5-90.8)	92.7 (89.6-94.1)
**2004-2005**	88.3 (87.5-89.1)	91.8 (89.7-93.9)	90.2 (88.4-92.6)	93.3 (90.5-94.9)
**2005-2006**	88.0 (87.2-88.8)	91.5 (89.7-93.2)	89.5 (87.8-91.5)	92.9 (90.3-94.4)
**2006-2007**	88.4 (87.5-89.3)	92.1 (90.1-94.1)	90.7 (88.5-92.7)	93.4 (91.0-95.2)
**2007-2008**	88.1 (87.2-88.9)	91.5 (89.9-93.0)	89.7 (87.9-91.7)	93.0 (90.7-94.5)
**2008-2009**	88.1 (87.3-88.8)	91.5 (90.0-93.0)	90.0 (88.3-91.9)	93.4 (91.2-94.8)
**2009-2010**	87.9 (87.2-88.7)	91.3 (90.0-93.0)	89.8 (88.2-91.9)	93.2 (90.8-94.7)
**2010-2011**	88.0 (87.3-88.8)	91.4 (90.0-93.0)	90.0 (88.3-92.0)	93.3 (91.2-94.8)
**2011-2012**	88.1 (87.4-88.8)	91.4 (90.1-92.6)	90.2 (88.6-92.0)	93.3 (91.4-95.0)
**2012-2013**	88.1 (87.4-88.9)	91.4 (90.1-92.6)	90.0 (88.5-92.0)	93.4 (91.1-94.9)
**2013-2014**	88.1 (87.3-88.8)	91.5 (90.4-92.7)	90.3 (88.8-92.0)	93.5 (91.7-95.2)
**2014-2015**	88.3 (87.4-88.9)	91.6 (90.4-93.0)	90.9 (89.3-92.5)	93.7 (91.8-95.3)
**2015-2016**	87.7 (86.8-88.7)	92.2 (90.7-94.2)	92.0 (90.1-94.1)	94.7 (92.9-100)
**2016-2017**	88.2 (87.4-88.9)	91.8 (90.6-93.3)	91.7 (89.8-93.4)	94.4 (92.3-96.5)
**2017-2018**	88.2 (87.3-88.9)	91.7 (90.4-93.1)	91.6 (89.7-93.4)	94.4 (92.4-100)
**2018-2019**	88.2 (87.3-88.9)	91.9 (90.6-92.9)	91.8 (89.9-93.9)	94.5 (92.6-100)

For peer review only

Table 5: Medians (25th – 75th centiles) of ratio for users ≥50th centile over the rest of consultations by consultation type, all practices

Year	All consultations by all staff	All consultations by GPs	Face-to-face consultations by all staff	Face-to-face consultations by GPs
2000-2001	6.1 (5.7-6.4)	7.5 (6.5-8.9)	6.0 (5.5-6.7)	7.7 (6.3-9.5)
2001-2002	6.3 (5.9-6.7)	7.7 (6.6-9.1)	6.4 (5.8-7.1)	8.2 (6.6-9.6)
2002-2003	6.5 (6.0-6.9)	8.0 (6.9-9.7)	6.5 (6.0-7.4)	8.5 (6.8-9.8)
2003-2004	6.6 (6.2-7.1)	8.4 (7.3-10.1)	6.7 (6.2-7.6)	8.7 (7.2-10.1)
2004-2005	7.0 (6.6-7.6)	9.2 (7.6-12.0)	7.5 (6.6-9.7)	9.4 (7.7-11.4)
2005-2006	6.9 (6.5-7.4)	9.0 (7.8-10.7)	7.1 (6.4-8.2)	9.2 (7.6-10.6)
2006-2007	7.2 (6.6-7.8)	9.9 (8.0-12.6)	7.8 (6.8-10)	9.9 (8.0-12.1)
2007-2008	7.0 (6.6-7.5)	9.2 (7.9-10.8)	7.2 (6.5-8.5)	9.2 (7.7-10.7)
2008-2009	7.0 (6.6-7.5)	9.2 (8.1-11.0)	7.4 (6.6-8.6)	9.7 (8.2-11.4)
2009-2010	6.9 (6.5-7.4)	9.3 (8.0-11.0)	7.4 (6.6-8.6)	9.8 (8.0-11.2)
2010-2011	7.0 (6.6-7.5)	9.3 (8.0-11.0)	7.5 (6.6-8.6)	9.9 (8.3-11.4)
2011-2012	7.1 (6.6-7.6)	9.3 (8.1-10.7)	7.5 (6.8-8.7)	10.0 (8.4-11.4)
2012-2013	7.1 (6.7-7.6)	9.3 (8.2-10.6)	7.6 (6.8-8.8)	9.8 (8.3-11.4)
2013-2014	7.1 (6.6-7.6)	9.6 (8.5-11.0)	7.7 (7.0-8.9)	10.2 (8.7-11.9)
2014-2015	7.2 (6.7-7.7)	9.7 (8.6-11.3)	8.2 (7.3-9.5)	10.5 (8.9-12.1)
2015-2016	6.9 (6.4-7.5)	10.4 (8.6-14.4)	9.5 (7.7-11.9)	11.4 (9.6-14.2)
2016-2017	7.2 (6.6-7.7)	9.8 (8.7-12.1)	8.7 (7.6-10.5)	11.0 (9.3-13.0)
2017-2018	7.1 (6.6-7.7)	9.9 (8.7-11.8)	8.6 (7.6-10.8)	11.1 (9.2-12.9)
2018-2019	7.2 (6.6-7.8)	9.9 (8.8-11.8)	8.9 (7.8-11.2)	11.2 (9.6-13.3)

For peer review only

75th centile

Table 6 Medians (25th – 75th centiles) of 75th centile number of consultations by consultation type, all practices

Year	All consultations by all staff	All consultations by GPs	Face-to-face consultations by all staff	Face-to-face consultations by GPs
**2000-2001**	16 (14-18)	7 (6-10)	7 (6-9)	5 (4-6)
**2001-2002**	17.5 (15-20)	7 (6-9)	7 (6-9)	5 (4-6)
**2002-2003**	19 (16-22)	7 (6-9)	7 (6-9)	5 (4-6)
**2003-2004**	21 (17-24)	8 (6-10)	7 (6-9)	5 (4-6)
**2004-2005**	22 (18-26)	8 (7-10)	7 (6-9)	5 (4-6)
**2005-2006**	24 (19-28)	9 (7-11)	7 (6-10)	5 (4-6)
**2006-2007**	24 (20-29)	9 (7-11)	7 (6-9)	4 (4-6)
**2007-2008**	26 (21-30)	9 (7-11)	7 (6-9)	5 (4-6)
**2008-2009**	27 (23-31.5)	9 (8-11)	7 (6-9)	5 (4-6)
**2009-2010**	28 (24-33)	10 (8-12)	7 (6-9)	5 (4-6)
**2010-2011**	28 (24-33)	10 (8-12)	7 (6-9)	5 (4-6)
**2011-2012**	29 (25-34)	10 (9-12)	7 (6-9)	5 (4-6)
**2012-2013**	30 (26-35)	10 (9-12)	7 (6-9)	5 (4-6)
**2013-2014**	32 (27-37)	11 (9-13)	7 (6-9)	5 (4-6)
**2014-2015**	32 (28-37)	11 (10-13)	7 (6-9)	5 (4-6)
**2015-2016**	32 (28-37)	11 (9-13)	7 (6-8)	5 (4-5)
**2016-2017**	32 (28-38)	11 (9-13)	7 (6-8)	5 (4-6)
**2017-2018**	33 (29-38)	11 (9-13)	7 (6-8)	4 (3-5)
**2018-2019**	35 (30-40)	11 (10-13)	7 (6-8)	4 (3-5)

For peer review only

Table 7 Medians (25th – 75th centiles) of proportion of high volume (≥ 75th centile) by consultation type, all practices

Year	All consultations by all staff	All consultations by GPs	Face-to-face consultations by all staff	Face-to-face consultations by GPs
**2000-2001**	62.4 (61.2-63.9)	65.8 (63.5-69.1)	63.7 (61.6-66.0)	66.4 (63.8-69.9)
**2001-2002**	62.9 (61.6-64.3)	66.5 (64.0-69.1)	63.7 (61.8-66.4)	66.8 (64.3-70.4)
**2002-2003**	63.1 (61.9-64.6)	67.4 (64.7-69.8)	64.4 (62.3-66.7)	67.9 (64.3-71.3)
**2003-2004**	63.5 (62.2-64.8)	67.7 (65.4-70.2)	64.9 (62.7-67.3)	68.2 (65.0-71.8)
**2004-2005**	64.3 (63.0-65.7)	68.9 (66.1-71.7)	66.2 (63.9-69.0)	69.5 (66.1-73.4)
**2005-2006**	63.8 (62.6-65.3)	68.2 (65.9-70.8)	65.4 (63.2-67.7)	68.9 (65.7-72.2)
**2006-2007**	64.6 (63.0-66.0)	69.3 (66.3-71.9)	66.4 (64.0-69.4)	70.3 (66.7-73.9)
**2007-2008**	64.1 (62.7-65.4)	68.4 (66.4-70.7)	65.5 (63.2-68.0)	69.2 (66.0-72.8)
**2008-2009**	64.2 (62.7-65.5)	68.2 (66.5-70.7)	65.8 (63.9-68.6)	69.5 (66.4-72.7)
**2009-2010**	63.8 (62.6-65.2)	68.4 (66.2-70.5)	65.8 (63.7-68.0)	69.5 (66.6-72.4)
**2010-2011**	64.0 (62.7-65.5)	68.3 (66.4-70.4)	66.3 (64.2-68.5)	69.5 (66.8-72.4)
**2011-2012**	64.3 (62.9-65.4)	68.4 (66.5-70.2)	66.2 (64.1-68.8)	69.8 (67.0-73.0)
**2012-2013**	64.3 (62.9-65.6)	68.4 (66.8-70.2)	66.5 (64.6-68.8)	69.6 (67.0-73.1)
**2013-2014**	64.2 (62.9-65.7)	68.7 (66.8-70.5)	66.7 (64.6-69.0)	70.1 (67.4-73.4)
**2014-2015**	64.5 (62.9-65.9)	69.0 (67.3-70.8)	67.3 (65.3-69.4)	70.6 (67.9-73.8)
**2015-2016**	63.7 (62.4-65.5)	69.6 (67.4-72.5)	68.8 (66.4-71.7)	72.3 (69.1-76.8)
**2016-2017**	64.3 (62.8-65.8)	69.2 (67.3-71.2)	68.2 (66.0-70.7)	71.5 (68.3-75.6)
**2017-2018**	64.1 (62.7-65.8)	69.0 (67.1-71.3)	68.4 (65.5-71.4)	72.1 (69.0-76.1)
**2018-2019**	64.2 (62.6-65.5)	69.0 (67.3-71.3)	68.6 (66.2-71.2)	72.6 (69.3-77.2)

For peer review only

Table 8: Medians (25th – 75th centiles) of ratio for users ≥75th centile over the rest of consultations by consultation type, all practices

Year	All consultations by all staff	All consultations by GPs	Face-to-face consultations by all staff	Face-to-face consultations by GPs
2000-2001	4.7 (4.5-5.0)	5.1 (4.8-5.7)	4.6 (4.4-4.9)	5.0 (4.6-5.5)
2001-2002	4.8 (4.6-5.1)	5.3 (4.9-5.8)	4.7 (4.4-5.0)	5.1 (4.7-5.7)
2002-2003	4.9 (4.6-5.2)	5.4 (5.0-6.1)	4.8 (4.5-5.2)	5.1 (4.8-5.8)
2003-2004	5.0 (4.7-5.3)	5.7 (5.2-6.3)	4.9 (4.6-5.3)	5.3 (4.9-6.0)
2004-2005	5.2 (4.9-5.5)	6.0 (5.4-6.7)	5.2 (4.8-5.8)	5.7 (5.2-6.6)
2005-2006	5.1 (4.8-5.4)	5.8 (5.4-6.5)	5.0 (4.7-5.5)	5.5 (5.0-6.3)
2006-2007	5.3 (4.9-5.6)	6.2 (5.5-7.0)	5.3 (4.9-6.0)	5.9 (5.2-6.8)
2007-2008	5.2 (4.9-5.5)	5.9 (5.5-6.5)	5.1 (4.7-5.5)	5.6 (5.1-6.2)
2008-2009	5.2 (4.9-5.5)	5.9 (5.5-6.6)	5.1 (4.8-5.6)	5.6 (5.2-6.3)
2009-2010	5.1 (4.9-5.4)	6.0 (5.5-6.5)	5.1 (4.8-5.6)	5.6 (5.2-6.3)
2010-2011	5.2 (4.9-5.5)	6.0 (5.5-6.5)	5.2 (4.8-5.6)	5.6 (5.2-6.2)
2011-2012	5.2 (4.9-5.5)	6.0 (5.6-6.5)	5.2 (4.9-5.7)	5.7 (5.3-6.3)
2012-2013	5.2 (4.9-5.6)	6.0 (5.6-6.5)	5.3 (4.9-5.7)	5.8 (5.3-6.4)
2013-2014	5.2 (5.0-5.6)	6.1 (5.7-6.6)	5.3 (5.0-5.8)	5.9 (5.4-6.5)
2014-2015	5.3 (5.0-5.6)	6.2 (5.8-6.7)	5.4 (5.1-6.0)	6.0 (5.5-6.7)
2015-2016	5.2 (4.8-5.6)	6.4 (5.9-7.3)	5.9 (5.3-6.6)	6.6 (5.8-7.7)
2016-2017	5.3 (4.9-5.6)	6.3 (5.8-6.9)	5.7 (5.2-6.3)	6.3 (5.7-7.3)
2017-2018	5.2 (4.9-5.6)	6.3 (5.7-6.8)	5.7 (5.2-6.3)	6.4 (5.8-7.4)
2018-2019	5.2 (4.9-5.6)	6.3 (5.8-6.9)	5.7 (5.3-6.4)	6.5 (5.8-7.7)

For peer review only

Twelve or more consultations

Table 9 Medians (25th – 75th centiles) of proportion of patients with 12 or more consultations by consultation type, all practices

Year	All consultations by all staff	All consultations by GPs	Face-to-face consultations by all staff	Face-to-face consultations by GPs
**2000-2001**	36.0 (30.5-40.5)	12.6 (7.5-20.1)	10.6 (7.9-15.6)	4.9 (3.4-9.3)
**2001-2002**	38.9 (32.9-42.8)	12.5 (8.1-17.9)	11.2 (7.9-16.7)	4.6 (3.0-8.4)
**2002-2003**	40.9 (34.7-45.9)	12.7 (7.7-18.2)	10.7 (7.5-16.4)	4.3 (2.8-7.7)
**2003-2004**	43.3 (37.2-48.4)	14.4 (10.2-19.5)	10.8 (7.5-18.1)	4.2 (2.6-7.5)
**2004-2005**	44.3 (37.8-49.5)	15.7 (11.6-20.7)	11.2 (7.4-18.4)	4.1 (2.5-7.9)
**2005-2006**	47.2 (40.7-52.5)	17.0 (13.3-22.7)	11.9 (8.1-20.1)	4.3 (2.7-8.0)
**2006-2007**	47.3 (41.0-52.4)	17.5 (13.2-22.8)	11.6 (7.7-19.5)	4.2 (2.6-8.2)
**2007-2008**	49.5 (44.2-53.9)	18.0 (13.0-23.3)	11.8 (8.2-17.9)	4.2 (2.8-7.2)
**2008-2009**	51.1 (46.2-55.2)	19.0 (14.3-23.7)	11.7 (8.4-16.9)	4.4 (2.9-6.6)
**2009-2010**	52.8 (48.2-56.6)	20.0 (15.4-25.4)	12.2 (9.0-17.0)	4.7 (2.9-6.8)
**2010-2011**	52.7 (48.3-56.7)	20.7 (16.4-25.3)	12.1 (8.6-16.3)	4.7 (2.9-7.0)
**2011-2012**	53.8 (49.4-57.5)	21.8 (17.3-26.2)	12.2 (8.7-16.1)	4.7 (3.0-7.0)
**2012-2013**	54.5 (50.4-58.4)	22.3 (18.1-27.4)	12.4 (9.1-16.4)	4.6 (3.0-7.4)
**2013-2014**	55.6 (51.2-59.7)	23.8 (19.4-28.6)	12.5 (8.9-16.4)	4.6 (2.9-7.4)
**2014-2015**	56.0 (51.8-60.1)	24.1 (19.8-29.2)	12.3 (8.8-16.3)	4.6 (2.9-7.3)
**2015-2016**	56.2 (52.1-60.5)	23.0 (18.7-28.0)	11.7 (8.3-15.5)	4.5 (2.4-7.2)
**2016-2017**	56.4 (52.1-60.4)	24.0 (18.9-29.2)	11.8 (8.2-16.1)	4.5 (2.5-7.7)
**2017-2018**	56.9 (52.9-60.9)	24.0 (19.6-28.9)	11.4 (7.4-15.8)	4.2 (2.2-7.3)
**2018-2019**	58.4 (54.1-61.8)	24.7 (19.9-29.2)	11.3 (7.3-15.4)	4.3 (2.1-7.1)

For peer review only

Table 10 Medians (25th – 75th centiles) of proportion of high volume (12 or more consultations) by consultation type, all practices

Year	All consultations by all staff	All consultations by GPs	Face-to-face consultations by all staff	Face-to-face consultations by GPs
**2000-2001**	73.9 (68.3-78.5)	43.1 (30.6-56.4)	35.8 (28.1-47.0)	20.7 (15.7-34.1)
**2001-2002**	77.6 (71.5-81.3)	43.2 (31.7-53.7)	36.8 (28.6-48.1)	20.2 (15.1-31.7)
**2002-2003**	79.8 (74.1-83.8)	43.6 (31.1-54.8)	36.1 (28.3-49.3)	19.4 (14.0-29.8)
**2003-2004**	82.3 (76.5-85.9)	48.3 (37.5-57.2)	37.3 (28.4-52.7)	19.4 (13.8-31.0)
**2004-2005**	83.8 (78.2-87.0)	51.7 (42.4-60.2)	39.3 (29.7-55.8)	19.7 (13.6-33.3)
**2005-2006**	85.6 (80.5-88.8)	54.1 (45.7-62.5)	40.4 (30.7-57.0)	20.1 (14.4-33.4)
**2006-2007**	86.1 (81.5-89.1)	55.9 (46.9-64.2)	41.1 (31.8-56.0)	20.4 (14.8-33.2)
**2007-2008**	87.2 (83.3-89.8)	55.7 (46.2-64.0)	40.2 (31.4-52.6)	20.4 (14.8-30.6)
**2008-2009**	88.3 (84.9-90.6)	57.5 (48.5-64.5)	40.1 (32.0-51.0)	21.2 (15.1-28.9)
**2009-2010**	88.9 (86.2-91.1)	59.3 (50.6-66.6)	41.0 (33.2-51.0)	22.0 (15.5-29.5)
**2010-2011**	89.1 (86.4-91.3)	59.9 (52.3-66.7)	41.0 (32.7-50.1)	21.9 (15.7-30.1)
**2011-2012**	89.8 (87.3-91.7)	61.6 (54.1-68.4)	41.7 (33.0-49.5)	21.9 (16.0-30.0)
**2012-2013**	90.2 (87.8-92.1)	62.8 (56.1-70.3)	42.4 (33.4-50.0)	22.3 (16.1-31.3)
**2013-2014**	90.7 (88.3-92.6)	65.1 (58.4-71.4)	42.5 (33.8-50.5)	22.1 (15.8-32.1)
**2014-2015**	90.9 (88.7-92.8)	66.2 (59.6-72.0)	43.2 (34.0-50.5)	22.9 (16.1-32.4)
**2015-2016**	90.6 (88.3-92.5)	65.7 (58.4-71.6)	43.0 (33.7-50.8)	23.1 (15.2-32.4)
**2016-2017**	91.2 (88.9-93.0)	66.1 (58.7-72.7)	42.9 (33.5-51.5)	22.7 (14.5-34.0)
**2017-2018**	91.3 (89.3-93.2)	65.8 (59.2-72.2)	41.7 (32.0-51.5)	22.9 (14.0-32.5)
**2018-2019**	92.0 (90.2-93.6)	67.0 (59.9-73.0)	41.6 (31.5-50.5)	22.2 (13.4-32.0)

For peer review only

For peer review only - http://bmjopen.bmj.com/site/about/guidelines.xhtml

STROBE Statement—Checklist of items that should be included in reports of ***cohort studies***

	Item No	Recommendation	
**Title and abstract**	1	(*a*) Indicate the study's design with a commonly used term in the title or the abstract	p1
		(*b*) Provide in the abstract an informative and balanced summary of what was done and what was found	p3
**Introduction**			
Background/rationale	2	Explain the scientific background and rationale for the investigation being reported	p4
Objectives	3	State specific objectives, including any prespecified hypotheses	p5
**Methods**			
Study design	4	Present key elements of study design early in the paper	p5
Setting	5	Describe the setting, locations, and relevant dates, including periods of recruitment, exposure, follow-up, and data collection	p5
Participants	6	(*a*) Give the eligibility criteria, and the sources and methods of selection of participants. Describe methods of follow-up	p5
		(*b*) For matched studies, give matching criteria and number of exposed and unexposed	NA
Variables	7	Clearly define all outcomes, exposures, predictors, potential confounders, and effect modifiers. Give diagnostic criteria, if applicable	p5-6
Data sources/ measurement	8*	For each variable of interest, give sources of data and details of methods of assessment (measurement). Describe comparability of assessment methods if there is more than one group	p5-6
Bias	9	Describe any efforts to address potential sources of bias	p6
Study size	10	Explain how the study size was arrived at	p5
Quantitative variables	11	Explain how quantitative variables were handled in the analyses. If applicable, describe which groupings were chosen and why	p5-6
Statistical methods	12	(*a*) Describe all statistical methods, including those used to control for confounding	p6
		(*b*) Describe any methods used to examine subgroups and interactions	p6
		(*c*) Explain how missing data were addressed	NA
		(*d*) If applicable, explain how loss to follow-up was addressed	NA
		(*e*) Describe any sensitivity analyses	p6
**Results**			
Participants	13*	(a) Report numbers of individuals at each stage of study—eg numbers potentially eligible, examined for eligibility, confirmed eligible, included in the study, completing follow-up, and analysed	p7
		(b) Give reasons for non-participation at each stage	NA
		(c) Consider use of a flow diagram	NA
Descriptive data	14*	(a) Give characteristics of study participants (eg demographic, clinical, social) and information on exposures and potential confounders	NA
		(b) Indicate number of participants with missing data for each variable of interest	NA
		(c) Summarise follow-up time (eg, average and total amount)	NA
Outcome data	15*	Report numbers of outcome events or summary measures over time	p7
Main results	16	(*a*) Give unadjusted estimates and, if applicable, confounder-adjusted estimates and their precision (eg, 95% confidence interval). Make clear which confounders were adjusted for and why they were included	p7
		(*b*) Report category boundaries when continuous variables were categorized	NA
		(*c*) If relevant, consider translating estimates of relative risk into absolute risk for a meaningful time period	p7

For peer review only - http://bmjopen.bmj.com/site/about/guidelines.xhtml

Other analyses	17	Report other analyses done—eg analyses of subgroups and interactions, and sensitivity analyses	p8
**Discussion**			
Key results	18	Summarise key results with reference to study objectives	p8
Limitations	19	Discuss limitations of the study, taking into account sources of potential bias or imprecision. Discuss both direction and magnitude of any potential bias	p9-10
Interpretation	20	Give a cautious overall interpretation of results considering objectives, limitations, multiplicity of analyses, results from similar studies, and other relevant evidence	p10
Generalisability	21	Discuss the generalisability (external validity) of the study results	p10
**Other information**			
Funding	22	Give the source of funding and the role of the funders for the present study and, if applicable, for the original study on which the present article is based	p11

*Give information separately for exposed and unexposed groups.

Note: An Explanation and Elaboration article discusses each checklist item and gives methodological background and published examples of transparent reporting. The STROBE checklist is best used in conjunction with this article (freely available on the Web sites of PLoS Medicine at http://www.plosmedicine.org/, Annals of Internal Medicine at http://www.annals.org/, and Epidemiology at http://www.epidem.com/). Information on the STROBE Initiative is available at http://www.strobe-statement.org.

For peer review only

For peer review only - http://bmjopen.bmj.com/site/about/guidelines.xhtml

BMJ Open

Consultation patterns and frequent attenders in UK primary care from 2000 to 2019: a cohort analysis of consultation events across 845 general practices.

Journal:	*BMJ Open*
Manuscript ID	bmjopen-2021-054666.R2
Article Type:	Original research
Date Submitted by the Author:	08-Sep-2021
Complete List of Authors:	Kontopantelis, Evangelos; The University of Manchester, Division of Informatics, Imaging and Data Science; NIHR School for Primary Care Research, University of Oxford<br>Panagioti, Maria; The University of Manchester, Division of Population Health, Health Services Research & Primary Care; The University of Manchester, NIHR Greater Manchester Patient Safety Translational Research Centre<br>Farragher, Tracey; The University of Manchester, Division of Population Health, Health Services Research & Primary Care<br>Munford, Luke; The University of Manchester, Division of Population Health, Health Services Research & Primary Care<br>Parisi, Rosa; The University of Manchester, Division of Informatics, Imaging and Data Science<br>Planner, Claire; The University of Manchester, Division of Population Health, Health Services Research & Primary Care; NIHR School for Primary Care Research, University of Oxford<br>Spooner, Sharon; The University of Manchester, Health Organisation, Policy and Economics (HOPE) Group, Centre for Primary Care & Health Services Research; The University of Manchester, Division of Population Health, Health Services Research & Primary Care<br>Tse, Alice; The University of Manchester, Division of Population Health, Health Services Research & Primary Care<br>Ashcroft, Darren; The University of Manchester, NIHR Greater Manchester Patient Safety Translational Research Centre; The University of Manchester, Division of Pharmacy & Optometry<br>Esmail, Aneez; The University of Manchester, Division of Population Health, Health Services Research & Primary Care; NIHR School for Primary Care Research, University of Oxford
**Primary Subject Heading**:	General practice / Family practice
Secondary Subject Heading:	Health economics, Health services research, Medical management
Keywords:	PRIMARY CARE, Health policy < HEALTH SERVICES ADMINISTRATION & MANAGEMENT, Organisation of health services < HEALTH SERVICES ADMINISTRATION & MANAGEMENT

For peer review only - http://bmjopen.bmj.com/site/about/guidelines.xhtml

I, the Submitting Author has the right to grant and does grant on behalf of all authors of the Work (as defined in the below author licence), an exclusive licence and/or a non-exclusive licence for contributions from authors who are: i) UK Crown employees; ii) where BMJ has agreed a CC-BY licence shall apply, and/or iii) in accordance with the terms applicable for US Federal Government officers or employees acting as part of their official duties; on a worldwide, perpetual, irrevocable, royalty-free basis to BMJ Publishing Group Ltd ("BMJ") its licensees and where the relevant Journal is co-owned by BMJ to the co-owners of the Journal, to publish the Work in this journal and any other BMJ products and to exploit all rights, as set out in our licence.

The Submitting Author accepts and understands that any supply made under these terms is made by BMJ to the Submitting Author unless you are acting as an employee on behalf of your employer or a postgraduate student of an affiliated institution which is paying any applicable article publishing charge ("APC") for Open Access articles. Where the Submitting Author wishes to make the Work available on an Open Access basis (and intends to pay the relevant APC), the terms of reuse of such Open Access shall be governed by a Creative Commons licence – details of these licences and which Creative Commons licence will apply to this Work are set out in our licence referred to above.

Other than as permitted in any relevant BMJ Author's Self Archiving Policies, I confirm this Work has not been accepted for publication elsewhere, is not being considered for publication elsewhere and does not duplicate material already published. I confirm all authors consent to publication of this Work and authorise the granting of this licence.

For peer review only

Consultation patterns and frequent attenders in UK primary care from 2000 to 2019: a retrospective cohort analysis of consultation events across 845 general practices.

Evangelos Kontopantelis, Professor in Data Science and Health Services Research[1,2,3]
Maria Panagioti, Senior Lecturer[4,5]
Tracey Farragher, Lecturer in Healthcare Sciences[4]
Luke Munford, Lecturer in Health Economics[3,4]
Rosa Parisi, Research Fellow in Statistics[1,3]
Claire Planner, Research Associate[2,4]
Sharon Spooner, General Practitioner & Clinical Lecturer[2,3,4]
Alice Hoi Ning Tse, General Practitioner[4]
Darren M. Ashcroft, Professor of Pharmacoepidemiology[2,5,6]
Aneez Esmail, Professor of General Practice[2,4]

1 Division of Informatics, Imaging and Data Science, University of Manchester, Manchester, England
2 NIHR School for Primary Care Research, University of Oxford, Oxford, England
3 Health Organisation, Policy and Economics (HOPE) research group; University of Manchester, Manchester, England
4 Division of Population Health, Health Services Research & Primary Care, University of Manchester, Manchester, England
5 NIHR Greater Manchester Patient Safety Translational Research Centre, Manchester, England
6 Division of Pharmacy & Optometry, University of Manchester, Manchester, England

Corresponding author:

Evangelos Kontopantelis, PhD
e.kontopantelis@manchester.ac.uk
Professor in Data Science and Health Services Research
Division of Informatics, Imaging and Data Sciences
University of Manchester
Oxford Road
M13 9PL
Manchester
UK

Keywords: primary care, consultations, workload, frequent attendance

Word count: 3,726
Abstract: 380

For peer review only - http://bmjopen.bmj.com/site/about/guidelines.xhtml

Abstract

Objective

To describe the distribution of consultations at the practice level and examine whether increases are uniform or driven by people who consult more frequently.

Design

Retrospective cohort study.

Setting

UK general practice data from CPRD GOLD.

Participants

1,699,709,314 consultation events from 12,330,545 patients, in 845 general practices (1/4/2000, 31/3/2019).

Methods

Consultation information was aggregated by financial year into: all consultations/all staff; all consultations/GPs; face-to-face consultations/all staff; face-to-face consultations/GPs. Patients with a number of consultations above the 90th centile, within each year, were classified as frequent attenders. Negative binomial regressions examined the association between available practice characteristics and consultation distribution.

Results

Among frequent attenders, all consultations by GPs increased from a median (25th, 75th centile) of 13 (10 to 16) to 21 (18 to 25) and all consultations by all staff increased from 27 (23, 30) to 60 (51, 69) over the study period. Approximately 4/10 consultations of any type concerned frequent attenders and the proportion of consultations attributed to them increased over time, particularly for face-to-face consultations with GPs, from a median of 38.0% (35.9%, 40.3%) in 2000/1 to 43.0% (40.6%, 46.4%) in 2018/9. Regression analyses indicated decreasing trends over time for face-to-face consultations and increasing trends for all consultation types, for both GPs and all staff. Frequent attenders consulted approximately 5 times more than the rest of the practice population, on average, with adjusted incidence rate ratios ranging between 4.992 (95%CI: 4.917, 5.068) for face-to-face consultations with all staff, and 5.603 (95%CI: 5.560, 5.647) for all consultations with GPs.

Conclusions

Frequent attenders progressively contributed to increased workload in general practices across the UK, from 2000 to 2019. Important knowledge gaps remain in terms of the demographic, social and health characteristics of frequent attenders and how UK general practices can be prepared to meet the needs of these patients.

For peer review only

Strengths and limitations of this study

- This is a large observational study that analysed 1,699,709,314 consultation events from 12,330,545 patients, in 845 general practices, from 1 April 2000 to 31 March 2019.
- The unique contribution of our study is the investigation of distribution trends amongst the top 10% of consulters for all consultations (covering all staff working in general practice), all consultations with GPs (including face-to-face consultations and all other consultations), face to face consultations with all staff, and face to face consultations with GPs.
- This is an observational study, using a database of Electronic Health Records, and it is dependent on the consultations and consultation types being recorded accurately; we were not able to ascertain information on patient and practice characteristics which is critical to understanding why this group of frequent attending patients consult their GPs.
- This is a practice-level analysis that did not focus on patients, and future work should examine persistent frequent attendance, and the patient and practice characteristics associated with frequent attendance.

For peer review only

For peer review only - http://bmjopen.bmj.com/site/about/guidelines.xhtml

Introduction

General practice in the United Kingdom (UK) has been facing significant workload challenges for more than a decade. In a seminal paper published in 2016, Hobbs and colleagues showed that the crude annual consultation rate per person increased by 10.51%, from 4.67 in 2007-08 to 5.16 in 2013-14.[1] Using data covering nearly 100 million consultations from anonymised practices contributing data to the Clinical Practice Research Datalink (CPRD GOLD), their study found that there was also a substantial increase in average consultation duration and total patient-facing clinical workload (by 16%) during this period. The authors concluded that the perceptions of a rapidly rising workload in UK general practice was well founded and compounded by the complexity of care that general practitioners (GPs) were having to provide including, for example, an increasing elderly population with multiple co-morbidities. The 2016 report by the King's Fund also confirmed that UK general practice is in crisis with a workload that has increased significantly over time. In addition to indicating that the increase was due to the needs of an ageing population and more people with complex conditions and multimorbidity, it was noted that the effect was compounded by initiatives to transfer care from hospitals to the community.[2]

Although existing work supports the view that GP workload is increasing over time,[1] up-to-date research evidence on workload rates is lacking. One important omission in previous estimates of UK GP workload is that the distribution of consultations within the patient population of general practices has not been assessed. In the Netherlands, Smits and colleagues identified a group of patients, referred to as persistent frequent attenders, occupying the top 10% of age and gender adjusted attenders over three continuous years. This group of frequent attenders may have special health needs, and may require different management in practice due to their distinct clinical and psychological characteristics.[3 4] As most of the literature on frequent attenders has been produced in the Netherlands, the generalisability of these findings and potential solutions in the UK needs to be evaluated. Pilot work in the UK supports these findings and could mean that the top 10% of attenders in the UK general practice are responsible for utilising between 30-50% of all GP consultations.[5] However, there is a paucity of large studies in the UK to establish firm estimates of the prevalence of frequent attendance in the GP workload.

Moreover, working arrangements within UK general practices change rapidly and dynamically. Major reforms are being implemented in response to the 2016 General Practice Forward View which could have an impact on workload rates.[6] For example, tele(e)-consultation options with GPs and other staff members of general practices have become increasingly prevalent, reaching a peak during the Covid-19 pandemic.[7 8] The workforce composition of general practices has also changed as more allied health professionals (e.g. advanced nurse practitioners, clinical pharmacists, and physician associates) and non-clinical staff have been appointed to increase the skill-mix in general practice and better serve the patient needs.[9 10] In addition, the health needs of local populations may affect consultation rates and workload demands differently in sub-regions within the UK .[11 12] Thus, potential variations in the GP workload across geographic regions need to be identified to tailor potential solutions.

This study provides new information about this under-researched topic by analysing the distribution of consultations within general practices and practice populations, with a focus on frequent attenders.

For peer review only - http://bmjopen.bmj.com/site/about/guidelines.xhtml

More specifically we examined: a) different ways to describe consultation distributions in a way that is informative to clinical practice, dichotomising into high and low attendance groups; b) how these vary across practices and regions, by consultation type; c) if and how they have changed over time. We also provide contemporary information about consultation rates to update the previous similar analysis undertaken five years ago.[1]

Methods

Data

We did a retrospective cohort study using the Clinical Practice Research Datalink (CPRD) GOLD database, which holds anonymised clinical information on patients, from participating general practices, including consultations, diagnoses, treatments, testing and referrals. Consultations include all contacts with the practice and will include requests for repeat prescriptions. They will also include consultations with practice staff (practice nurses, healthcare assistants, social prescribers, nurse practitioners and other allied health professional who are increasingly being employed by GPs). Some patients will contact a practice to speak to reception staff and these are usually identified as administrative episodes so will not be counted as consultations. Non face to face consultations will have included telephone and online consultations.

We analysed consultations data from 1 April 2000 until 31 March 2019, in annual bins of financial years. The number of practices in CPRD GOLD varied from 407 in 2000/1, to 740 in 2008/9, to 389 in 2018/19. Of these, only 113 practices contributed data throughout the whole of the study period. Although the database is representative of the UK in terms of age, gender and deprivation,[13] it is only broadly geographically representative since it collects data from practices using the Vision clinical system, and clinical system usage is geographically clustered in the UK.[14] For example, Scottish general practices are more heavily represented in more recent years of the CPRD database. Socio-economic deprivation of the practice location was available, as measured by the 2015 English Index of Multiple Deprivation (IMD) which is a composite score across seven domains: Income; Employment; Education; Skills and Training; Health and Disability; Crime; Barriers to Housing Services; Living Environment.[15] The Scottish, Welsh and Northern Irish IMDs are very similar, but with small domain changes to capture different country dynamics. Consultation information was aggregated within each financial year, for each active patient (registered for at least one day during the respective year). We aggregated the information into four consultation types: 1) all consultation types (including administrative) by all practice staff (health worker or administrator); 2) all consultation types by GPs; 3) face-to-face consultations by all staff; and 4) face-to-face consultations by all GPs (Supplementary file: Tables 1 & 2).

Statistical analyses

The distribution of consultations within each practice was quantified using various approaches, to allow for within and between practice comparisons over time. First, we calculated the mean, 10th, 25th, 50th (median), 75th and 90th centile of each of the four consultation types, for each practice within each financial year. Next we used the 50th, 75th and 90th centile to aggregate the consultation volume into dichotomous patient groups: those with a number of consultations in a given year below the 50th (or

For peer review only - http://bmjopen.bmj.com/site/about/guidelines.xhtml

75th or 90th) centile and those with a number of consultations equal to or higher than the 50th (or 75th or 90th) centile. We report the percentage of consultations attributed to the top group (equal to or above 50th, 75th or 90th centile), within each year. Finally, to allow for comparisons across practices, we used an arbitrary threshold of 12 consultations within a year to dichotomise volume, across each consultation type. As for centiles, we report the percentage of practice consultations attributed to people with 12 or more, within each year. The reported percentages, for centiles and 12 consultations, and for each practice within a year, can be expressed as:

$$p_{ij} = 100 * con_{ij1}/(con_{ij1} + con_{ij2})$$

Where:

$i = 1,\dots,4$ the consultation type
j denotes the 50th, 75th, 90th centile or 12 consultations;
con_{ij1} is the total consultation volume for the top group;
con_{ij2} is the total consultation volume for the bottom group.

Since the groups were not always balanced as per the centiles (e.g. dichotomising along the 50th centile did not result in two equal groups of patients), due to the relative small numbers of consultations for the average patient, we also calculated the ratio of consultations of the top group over the bottom group. This can be expressed as:

$$r_{ij} = {}^{(con_{ij1}/pat_{ij1})}/_{(con_{ij2}/pat_{ij2})}$$

Where
pat_{ij1} is the number of patients in the top group;
pat_{ij2} is the number of patients in the bottom group.

We report these metrics in violin plots,[16] across all practices and by region, over time. Violin plots can be interpreted as box plots (including the median as a marker and a box indicating the interquartile range), overlaid with the density of the distribution for better visualisation.

We used negative binomial regression models for key outcomes of interest (50th, 75th, 90th centile and 12 consultations), to examine the association between available practice characteristics and imbalanced consultation distribution, with a focus on frequent attendance. The outcome was the average number of consultations per patient within each of the two groups, for each practice within a year, adjusted for the number of patients within each group (exposure term). The binary group predictor (top/bottom group) was interacted with each of the following practice characteristics, in separate models: list size (time varying), region, and practice location deprivation as measured by the 2015 Index of Multiple Deprivation.[15]

All analyses were conducted with Stata v16 and an alpha level of 5% was used for the inferential statistics. We focused on the 90th centile analyses in the main paper, splitting the population within a practice to the top 10% in terms of service usage within a year, and contrasting it to the remaining

For peer review only - http://bmjopen.bmj.com/site/about/guidelines.xhtml

90% of the practice population. As a sensitivity, we analysed data only from the 113 practices which were active throughout the whole of the study period.

Patient and Public Involvement

The research was discussed with a Patient and Public Involvement group (PRIMER, based at the Centre for Primary Care and Health Service Research at the University of Manchester) at a meeting held prior to analysis. The Group agreed that understanding consultation patterns was an important starting point for exploring the reasons for frequent attendance (e.g. availability of double appointments for patients with multi-morbidity), the extent to which the needs of frequent attenders are being met and their experience of primary care services.

For peer review only

Results

Overall, 1,699,709,314 consultation events across 12,330,545 patients in 845 general practices were analysed. We present the 90th centile analyses in the main paper, and results were consistent for the other dichotomies (median, 75th centile and 12 or more consultations in a year), with summary results presented in the Supplementary file (Tables 3 to 10). Over time, the average volume of all consultations for a single patient, with GPs and all staff increased. However, face-to-face consultations remained relatively stable and face-to-face consultations with GPs even decreased (Table 1).

Over time, face-to-face consultations per annum with GPs and all staff remained stable for the top 10% group, and in 2018/19 medians (25th & 75th centiles) were 8 (6 & 10) and 12 (10 & 14), respectively (Table 2). On the other hand, medians for all consultations with GPs and all staff greatly increased over time. All consultations with GPs increased from a median (25th & 75th centiles) of 13 (10 & 16) in 2000/1 to 21 (18 & 25) in 2018/19. Similarly, all consultations with all staff increased from 27 (23 & 30) in 2000/1 to 60 (51 & 69) over the study period. However, the picture was somewhat different when quantifying service usage in relative terms, with increases over time across all consultations types for the top 10% group (Table 3 and Figure 1). The percentage of face-to-face consultations attributed to the top 10% of users within a year increased from a median (25th & 75th centiles) of 38.0% (35.9% & 40.3%) to 43.0% (40.6% & 46.4%) for interactions with GPs, and from 35.8% (34.4% & 37.7%) to 40.1% (38.3% & 42.5%) for interactions with all staff. Percentage increases for all consultations were smaller, from a median (25th & 75th centiles) of 38.0 (35.7% & 40.1%) to 40.9 (39.2% & 42.7%) with GPs, and from 35.2 (33.9% & 36.5%) to 36.6 (35.3% & 38.0%) with all staff, over the study period. These increases in service usage for the top 10% group, across all four outcomes, were also reflected in the ratio of consultations, with the largest increase observed in face-to-face consultations with GPs, from a median (25th & 75th centiles) of 4.7 (4.5 & 5.1) in 2000/1 to 5.9 (5.4 & 6.6) in 2018/19 (Table 4 and Figure 2).

There was relatively little regional variability in the median percentage attribution of all four consultation categories, across all examined patient dichotomies. For example, the median percentage of face-to-face consultations with GPs for the top 10% of patients in terms of service usage with their respective practice, varied from 35% for Wales and West Midlands to 38% for Northern Ireland, over the whole study period.

For peer review only - http://bmjopen.bmj.com/site/about/guidelines.xhtml

Results from the negative binomial regression analyses indicated decreasing trends over time for face-to-face consultations and increasing trends for all consultation types, for both GPs and all staff (Table 5 and Figure 3). Adjusted incidence rate ratios (IRRs) for the top 10% consultations group ranged between 4.99 (95% CI: 4.92 to 5.07) for face-to-face consultations with all staff, and 5.60 (95% CI: 5.56 to 5.65) for all consultations with GPs, compared to the bottom 90% consultations group. Associations between deprivation and consultations in the two groups were weak. Some regional variation was observed, with Scotland associated with the highest rate ratios for face-to-face consultations with GPs, and the South West associated with the highest rate ratios for face-to-face consultations with all staff. Pseudo-R^2 values for the four models varied between 0.048 for face-to-face consultations by all staff and 0.109 for all consultations by all staff.

Results from analyses of the other dichotomies (75-25, 50-50, 12 or more consultations) and sensitivity analyses focusing on practices for the whole of the study period were broadly similar to our main results.

Discussion

Summary of findings

This study examined distribution trends in four types of consultations (all consultations by GPs; all consultations by all staff; face-to-face consultations by GPs; face-to-face consultations by all staff) among frequent attenders (the top 10% of all consulters) and the rest of consulters in the UK general practices over the period from 2000/1 to 2018/9.

We found that all consultations by GPs and all consultations by all staff have considerably increased over this time period. Specifically, all consultations by GPs per person increased from a median of 5 to 8 and all consultations by all staff increased from 11 to 25. However, face-to-face consultations by GPs and face-to-face consultations by all staff have remained static (and may even have decreased).

Frequent attenders had five times more consultations of any type compared to the rest of consulters over the study period. Among frequent attenders, all consultations by GPs increased from a median of 13 to 21 and all consultations by all staff increased from 27 to 60 over the study period. Approximately 4 out of 10 consultations of any type concerned frequent attenders and the proportion of consultations attributed to frequent attenders increased over time, particularly for face-to-face consultations with GPs (from 38% in 2000/1 to 43% in 2018/9). We found little evidence of regional variability in the attribution of all four consultation types, across all categories of consulters. The only exemption was that the distribution of face-to-face consultations with GPs was highest in Scotland and the distribution of face-to-face consultations with all staff was highest in Northern Ireland.

Research in context

The unique contribution of our study is the investigation of distribution trends in all four types of consultations among frequent attenders (the top 10% of consulters). No previous study in the UK has

For peer review only - http://bmjopen.bmj.com/site/about/guidelines.xhtml

examined the contribution that frequent attenders make to the workload of general practices. Our findings showed that frequent attenders account for an increasing proportion of face-to-face consultations with GPs (38-43%) and are responsible for nearly 40% of all four types of consultations fairly constantly over time. This striking finding suggests that a relatively small number of patients are accounting for a large proportion of GP workload including face-to-face consultations. This could be potentially attributed to a relative reduction in accessibility to face-to-face consultations for non-frequent attenders, which may be driven by a perception of some or all actors (patient/carer/clinician/practice staff) that the need for face-to-face contact is smaller in this patient group. Despite the importance of these findings, there is very little research on frequent attenders in the UK to make direct comparisons. Outside the UK, our findings are consistent with epidemiological studies conducted in the Netherlands which has also shown that frequent attenders were responsible for nearly 40% of the face-to-face consultations.[3] Similarly, a previous review on frequent attenders reported that the top 10% of attenders accounted for 30% to 50% of all primary care consultations.[17]

Our current knowledge of the demographic, clinical and social characteristics of frequent attenders is scarce in the UK whereas findings from Europe are also inconsistent. Our analysis suggests that frequent attenders are identified across all parts of the UK and that deprivation, practice size or regional variation are not drivers for the number of frequent attenders by practice. There is evidence from Europe that frequent attenders (and particularly persistent frequent attenders) are more likely to be female and older, present more social and psychiatric problems, receive more prescriptions of psychotropic medication, have more medically unexplained physical symptoms, and more chronic medical conditions.[3 17]Our study cannot determine whether these factors are the main driving force for frequent attendance but the contribution to overall GP workload needs further examination. A systematic review focused on children also found that frequent attendance in children was associated with presence of psychosocial and mental health problems, younger age, school absence, presence of chronic conditions, and high level of anxiety in their parents.[18] However, another systematic review on older adults found that frequent attendance in late life was mainly associated with presence and severity of chronic medical conditions whereas associations with mental conditions, drug use and social support were heterogeneous.[19] Thus, although there is evidence that frequent attenders differ in terms of demographic, social, mental and physical characteristics from the rest of consulters, their profile may vary across age groups and according to different definitions of frequent attendance (e.g. persistent or no-persistent). Our findings and PPI work strongly support the need for future research to characterise frequent attenders in UK general practice taking into consideration the evidence and challenges encountered by previous studies conducted mostly in Europe.

Moreover, this study confirms and extends the secular trends first identified by Hobbs and colleagues that activity in general practice has increased over time [1]. This previous study found that the crude annual consultation rate per person increased from 4.67 in 2007-08, to 5.16 in 2013-14 and that GP telephone consultation rates doubled, compared with a 5.20% rise in face-to-face consultations. Consistent with this, we found that all consultations by GPs have increased and that face-to-face consultations are not the main driver of this increase. This finding suggests that other modalities for consulting such as telephone or online consultations may have become more important in recent years. Face-to-face consultations may have reached a capacity ceiling and other forms of consultations may be used to address otherwise unmet health needs. Moreover, Hobbs et al [1] found that the greatest increases in rates of consultations were in GPs, with a rise of 12.37% per 10,000 person-years,

For peer review only - http://bmjopen.bmj.com/site/about/guidelines.xhtml

compared with 0.9% for practice nurses. In our study however, the highest increase over time was observed in all consultations by all staff with consultation medians increasing from 11 in 2000/1 to 25 in 2018/9. This impressive increase in all consultations by all staff may suggest that the increase in multi-disciplinary staff working in general practices has resulted in a wider range of practice staff dealing with patients and that it is not all driven by face-to-face consultations. This trend appears to predate the 2016 GP Forward View and is likely to continue.

Limitations

This study has a number of important limitations. First, we used our own definition of frequent attenders (top 10% of consulters) and therefore our findings are only broadly comparable with other studies which have used other definitions (i.e. top 10% of consulters over 3 years; top 25% stratified by age, gender of health conditions). It is reassuring that our definition of frequent attendance is consistent with the recommended definition in a recent systematic review,[20] that our sensitivity analyses showed similar results, and that our findings were similar with the findings from our previous pilot study. Second, patients who have left a general practice but have not been removed from the register (also known as "ghost" patients) may exaggerate the differences between top and bottom users, and we know that their numbers vary by region.[21] This is an observational study, using a database of Electronic Health Records, and it is dependent on the consultations and consultation types being recorded accurately. Since the UK primary care has been almost fully computerised by the start of this century,[22] it is extremely unlikely that any consultations fail to be recorded. However, the default setting in recording a consultation is face-to-face, and this needs to be changed by the health professional for other consultation types. Thus, face-to-face consultations may be overestimated, if some health professionals miss that step. In addition, the underlying workload across consultation types may differ systematically, on top of expected patient variation, but this is something we cannot capture within this study of practice aggregates. Although the CPRD GOLD database is representative in terms of deprivation and population characteristics,[13] it is collecting data from a single computer system (VISION) and the contributing practices are not uniformly distributed across English regions, while its market share is in decline.[14] Thus, generalisability to every English region is questionable, especially in relation to the sensitivity analyses where the sample is quite small and the distribution of the practices across regional and deprivation strata changes. IMD scores are standardised within each UK country, and between country comparisons cannot capture baseline differences across countries. However, our regression analyses on the relative difference between high and low deprivation, in the context of the outcome of interest, should be robust to these nuances. Finally, we have not examined persistent frequent attendance, or the patient or practice characteristics associated with frequent attendance.

Implications for Policy and Practice

Frequent attenders appear to be a major driver for the increase in consultations that have contributed to perceptions of increased workload in general practice. Whilst many of these patients may have co-morbidities and may need to be seen regularly, research suggest that they have wider social and psychological needs.[17] GPs should be looking at this group of patients more closely to understand who are they and why are they consulting more frequently. Our PPI work indicates that patients see this as critically important for improving quality of care and preventing patients from being stigmatised for frequent attendance. One key challenge for future policy is how can general practice be prepared to

For peer review only - http://bmjopen.bmj.com/site/about/guidelines.xhtml

provide services and support to frequent attenders without medicalising them but addressing the wider social and psychological problems that they may have.

The large increase in the general practice workload the last 20 years means that having extended multidisciplinary teams to meet a wide range of patient needs through a range of ways (e.g. remote consultations) is perhaps the only solution for sustaining a viable primary care. This change in the workforce composition of general practices is reflected in the activities that we report (medians for all consultations by GPs increased 5 to 8 whereas all consultations by all staff increased from 11 to 25) and echoes recent policy documents such as the 2016 General Practice Forward View.[6] It is also likely that a multidisciplinary workforce in general practice could also better address the wider, not always medical, needs of frequent attenders.

Conclusions

This is the first study to show that frequent attenders, a relatively small group of consulters (the top 10%), largely and progressively contribute to increased workload in general practices across the UK over the last 20 years. Important knowledge gaps remain in terms of the demographic, social and health characteristics of frequent attenders in the UK and how general practices can be prepared to meet the needs of these patients. We intend to explore these factors in a separate study. Our findings depict a new model of work in general practice whereby an increasing number of consultations are conducted by other staff members (rather than GPs) using alternative means (rather than face-to-face consultations). With the overall workload of general practices on continuous rise, multidisciplinary and technology-enabled general practices could better meet the multiple and wider needs of frequent attenders and the rest of consulters.

For peer review only

Acknowledgements

We thank the members of the PRIMER patient and public involvement group (funded through the NIHR School for Primary Care Research) for their input into the early stages of this research and to Julie Billsborough, Manoj Mistry, Carole Bennett and Ailsa Donnelly for their contributions.

Ethical approval

This study is based on data from the Clinical Practice Research Datalink GOLD obtained under license from the Medicines and Healthcare Products Regulatory Agency (MHRA). The data are provided by patients and collected by the NHS as part of their care and support. The interpretation and conclusions contained in this study are those of the authors alone, and not necessarily those of the MHRA, the National Institute for Health Research (NIHR), National Health Service (NHS), or the Department of Health. Approval to conduct this study using the CPRD was granted by the Independent Scientific Advisory Committee (ISAC) of the Medicine and Healthcare Regulatory Agency (MHRA) (protocol 20_192R). We thank the contributing patients and practices to the CPRD GOLD who have allowed their data to be used for research purposes.

Funding

The University of Manchester. The funder had no role in any aspect of this work.

Data availability statement

The data analysed in this study are subject to the following licenses/restrictions: Electronic health records are, by definition, considered “sensitive” data in the UK by the Data Protection Act and cannot be shared via public deposition because of information governance restriction in place to protect patient confidentiality. Access to data are available only once approval has been obtained through the individual constituent entity controlling access to the data. The primary care data can be requested via application to the Clinical Practice Research Datalink (www.cprd.com). Requests to access this dataset should be directed to enquiries@cprd.com.

Competing interests statement

All authors have completed the ICMJE uniform disclosure form and declare: support from the University of Manchester for the submitted work; no financial relationships with any organisations that might have an interest in the submitted work in the previous three years, no other relationships or activities that could appear to have influenced the submitted work.

Contributorship statement

The research idea originated with AE and AT, but all authors designed the study. EK extracted the data and conducted the analyses, LM and RP supported him in this. TF led the ISAC submission, for access to the CPRD GOLD database. EK, MP and AE drafted the manuscript, TF, LM, RP, CP, SS, AT and DA critically revised it.

For peer review only - http://bmjopen.bmj.com/site/about/guidelines.xhtml

Author Licence

I, the Submitting Author has the right to grant and does grant on behalf of all authors of the Work (as defined in the author licence), an exclusive licence and/or a non-exclusive licence for contributions from authors who are: i) UK Crown employees; ii) where BMJ has agreed a CC-BY licence shall apply, and/or iii) in accordance with the terms applicable for US Federal Government officers or employees acting as part of their official duties; on a worldwide, perpetual, irrevocable, royalty-free basis to BMJ Publishing Group Ltd ("BMJ") its licensees.

The Submitting Author accepts and understands that any supply made under these terms is made by BMJ to the Submitting Author unless you are acting as an employee on behalf of your employer or a postgraduate student of an affiliated institution which is paying any applicable article publishing charge ("APC") for Open Access articles. Where the Submitting Author wishes to make the Work available on an Open Access basis (and intends to pay the relevant APC), the terms of reuse of such Open Access shall be governed by a Creative Commons licence – details of these licences and which licence will apply to this Work are set out in our licence referred to above.

For peer review only

For peer review only - http://bmjopen.bmj.com/site/about/guidelines.xhtml

References

1. Hobbs FDR, Bankhead C, Mukhtar T, Stevens S, Perera-Salazar R, Holt T, et al. Clinical workload in UK primary care: a retrospective analysis of 100 million consultations in England, 2007-14. *Lancet* 2016;387:2323-30.
2. Baird B, Charles A, Honeyman M, Maguire D, Das P. *Understanding pressures in general practice*: King's Fund London, 2016.
3. Smits FT, Brouwer HJ, ter Riet G, van Weert HC. Epidemiology of frequent attenders: a 3-year historic cohort study comparing attendance, morbidity and prescriptions of one-year and persistent frequent attenders. *BMC Public Health* 2009;9:36.
4. Smits FT, Brouwer HJ, Zwinderman AH, Mohrs J, Smeets HM, Bosmans JE, et al. Morbidity and doctor characteristics only partly explain the substantial healthcare expenditures of frequent attenders: a record linkage study between patient data and reimbursements data. *BMC Fam Pract* 2013;14:138.
5. Patel S, Kai J, Atha C, Avery A, Guo B, James M, et al. Clinical characteristics of persistent frequent attenders in primary care: case-control study. *Fam Pract* 2015;32:624-30.
6. England N. General practice forward view. 2016.
7. Salisbury H. Helen Salisbury: Teleconsultations for all. *BMJ* 2020;370:m3211.
8. Salisbury C, Murphy M, Duncan P. The Impact of Digital-First Consultations on Workload in General Practice: Modeling Study. *J Med Internet Res* 2020;22:e18203.
9. Nelson P, Martindale AM, McBride A, Checkland K, Hodgson D. Skill-mix change and the general practice workforce challenge. *Br J Gen Pract* 2018;68:66-67.
10. Nelson PA, Bradley F, Martindale AM, McBride A, Hodgson D. Skill-mix change in general practice: a qualitative comparison of three 'new' non-medical roles in English primary care. *Br J Gen Pract* 2019;69:e489-e98.
11. Kontopantelis E, Mamas MA, van Marwijk H, Ryan AM, Bower P, Guthrie B, et al. Chronic morbidity, deprivation and primary medical care spending in England in 2015-16: a cross-sectional spatial analysis. *BMC Med* 2018;16:19.
12. Spooner S, Gibson J, Checkland K, McBride A, Hodgson DE, Hann M, et al. Regional variation in practitioner employment in general practices in England: a comparative analysis. *Br J Gen Pract* 2020;70:e164-e71.
13. Herrett E, Gallagher AM, Bhaskaran K, Forbes H, Mathur R, van Staa T, et al. Data Resource Profile: Clinical Practice Research Datalink (CPRD). *Int J Epidemiol* 2015;44:827-36.
14. Kontopantelis E, Stevens RJ, Helms PJ, Edwards D, Doran T, Ashcroft DM. Spatial distribution of clinical computer systems in primary care in England in 2016 and implications for primary care electronic medical record databases: a cross-sectional population study. *Bmj Open* 2018;8:e020738.
15. Kontopantelis E, Mamas MA, van Marwijk H, Ryan AM, Buchan IE, Ashcroft DM, et al. Geographical epidemiology of health and overall deprivation in England, its changes and persistence from 2004 to 2015: a longitudinal spatial population study. *J Epidemiol Community Health* 2018;72:140-47.
16. Winter N. VIOPLOT: Stata module to produce violin plots with current graphics. 2008.
17. Vedsted P, Christensen MB. Frequent attenders in general practice care: a literature review with special reference to methodological considerations. *Public Health* 2005;119:118-37.
18. Al-Saffar MN, Hayhoe BW, Harris MJ, Majeed A, Greenfield G. Children as frequent attenders in primary care: a systematic review. *BJGP Open* 2020;4.
19. Welzel FD, Stein J, Hajek A, Konig HH, Riedel-Heller SG. Frequent attenders in late life in primary care: a systematic review of European studies. *BMC Fam Pract* 2017;18:104.
20. Luciano JV, Fernandez A, Pinto-Meza A, Lujan L, Bellon JA, Garcia-Campayo J, et al. Frequent attendance in primary care: comparison and implications of different definitions. *Br J Gen Pract* 2010;60:49-55.

For peer review only - http://bmjopen.bmj.com/site/about/guidelines.xhtml

21. Burch P, Doran T, Kontopantelis E. Regional variation and predictors of over-registration in English primary care in 2014: a spatial analysis. *J Epidemiol Community Health* 2018;72:532-38.
22. Kontopantelis E, Buchan I, Reeves D, Checkland K, Doran T. Relationship between quality of care and choice of clinical computing system: retrospective analysis of family practice performance under the UK's quality and outcomes framework. *BMJ Open* 2013;3.

For peer review only

For peer review only - http://bmjopen.bmj.com/site/about/guidelines.xhtml

Tables

Table 1: Medians (25th – 75th centiles) of average number of consultations per patient, by consultation type, all practices

Year	All consultations by all staff	All consultations by GPs	Face-to-face consultations by all staff	Face-to-face consultations by GPs
**2000-2001**	11.4 (9.8-13.0)	5.3 (4.3-6.8)	5.2 (4.5-6.3)	3.7 (3.2-4.7)
**2001-2002**	12.5 (10.5-14.2)	5.3 (4.4-6.4)	5.2 (4.5-6.4)	3.6 (3.1-4.4)
**2002-2003**	13.4 (11.2-15.7)	5.3 (4.3-6.6)	5.1 (4.3-6.4)	3.5 (2.9-4.3)
**2003-2004**	14.8 (12.0-17.3)	5.7 (4.6-6.9)	5.1 (4.3-6.8)	3.4 (2.8-4.2)
**2004-2005**	15.8 (12.5-18.3)	5.9 (4.9-7.1)	5.0 (4.1-6.7)	3.2 (2.7-4.2)
**2005-2006**	17.0 (13.8-20.1)	6.3 (5.4-7.6)	5.3 (4.4-7.0)	3.3 (2.8-4.3)
**2006-2007**	17.3 (14.0-20.5)	6.3 (5.3-7.7)	5.1 (4.2-6.9)	3.2 (2.7-4.1)
**2007-2008**	18.4 (15.3-21.2)	6.5 (5.4-7.7)	5.2 (4.3-6.6)	3.3 (2.8-4.1)
**2008-2009**	19.4 (16.5-22.4)	6.8 (5.6-7.9)	5.2 (4.4-6.4)	3.3 (2.8-3.9)
**2009-2010**	20.3 (17.7-23.3)	7.1 (5.9-8.4)	5.3 (4.5-6.4)	3.4 (2.8-4.0)
**2010-2011**	20.4 (17.8-23.8)	7.3 (6.1-8.4)	5.3 (4.4-6.3)	3.4 (2.8-4.0)
**2011-2012**	21.3 (18.5-24.6)	7.6 (6.4-8.8)	5.4 (4.5-6.4)	3.4 (2.8-4.0)
**2012-2013**	21.8 (19.0-25.2)	7.6 (6.6-9.1)	5.4 (4.6-6.4)	3.4 (2.9-4.1)
**2013-2014**	22.8 (19.7-26.3)	8.0 (6.9-9.5)	5.4 (4.6-6.4)	3.4 (2.8-4.1)
**2014-2015**	23.4 (20.3-26.7)	8.2 (6.9-9.7)	5.4 (4.6-6.3)	3.3 (2.8-4.1)
**2015-2016**	23.2 (20.0-26.8)	7.9 (6.6-9.3)	5.1 (4.3-6.1)	3.2 (2.6-3.9)
**2016-2017**	23.4 (20.3-27.4)	8.3 (6.8-9.7)	5.2 (4.3-6.2)	3.3 (2.6-4.0)
**2017-2018**	23.8 (20.7-27.4)	8.2 (7.0-9.7)	5.1 (4.1-6.1)	3.2 (2.5-4.0)
**2018-2019**	25.1 (21.7-28.7)	8.3 (7.0-9.7)	5.0 (4.2-6.0)	3.1 (2.5-3.9)

For peer review only

For peer review only - http://bmjopen.bmj.com/site/about/guidelines.xhtml

Table 2: Medians (25th – 75th centiles) of 90th centile number of consultations per patient, by consultation type, all practices

Year	All consultations by all staff	All consultations by GPs	Face-to-face consultations by all staff	Face-to-face consultations by GPs
**2000-2001**	27 (23-30)	13 (10-16)	12 (10-15)	9 (8-11)
**2001-2002**	30 (25-34)	13 (10-16)	12 (10-15)	8 (7-11)
**2002-2003**	32 (26-37)	13 (10-16)	12 (10-15)	8 (7-10)
**2003-2004**	35 (29-41)	14 (12-17)	12 (10-16)	8 (7-10)
**2004-2005**	38 (30-45)	15 (12-18)	12 (10-16)	8 (7-10)
**2005-2006**	41 (33-49)	16 (13-19)	12 (10-17)	8 (7-10)
**2006-2007**	42 (34-50)	16 (13-19)	12 (10-16)	8 (7-10)
**2007-2008**	44 (37-52)	16 (13-19)	12 (10-15)	8 (7-10)
**2008-2009**	47 (40-54)	17 (14-20)	12 (10-15)	8 (7-10)
**2009-2010**	49 (42-56)	18 (15-21)	12 (11-15)	8 (7-10)
**2010-2011**	49 (43-57)	18 (15-21)	12 (10-15)	8 (7-10)
**2011-2012**	51 (44-58)	19 (16-22)	13 (11-15)	8 (7-10)
**2012-2013**	52 (45-60)	19 (16-23)	13 (11-15)	8 (7-10)
**2013-2014**	55 (47-63)	20 (17-24)	13 (11-15)	8 (7-10)
**2014-2015**	56 (49-64)	20 (17-24)	13 (11-15)	8 (7-10)
**2015-2016**	55 (48-64)	20 (17-23)	13 (11-15)	8 (7-10)
**2016-2017**	56.5 (48-66)	21 (17-24)	12.5 (10-15)	8 (7-10)
**2017-2018**	57 (50-66)	20 (17-24)	12 (10-15)	8 (6-10)
**2018-2019**	60 (51-69)	21 (18-25)	12 (10-14)	8 (6-10)

For peer review only - http://bmjopen.bmj.com/site/about/guidelines.xhtml

Table 3: Medians (25th – 75th centiles) of proportion of high volume (≥ 90th centile) per patient, by consultation type, all practices

Year	All consultations by all staff	All consultations by GPs	Face-to-face consultations by all staff	Face-to-face consultations by GPs
**2000-2001**	35.2 (33.9-36.5)	38.0 (35.7-40.1)	35.8 (34.4-37.7)	38.0 (35.9-40.3)
**2001-2002**	35.5 (34.1-36.8)	38.4 (36.5-40.4)	36.3 (34.6-38.2)	38.5 (36.5-40.9)
**2002-2003**	35.6 (34.3-37.0)	38.7 (36.6-41.1)	36.5 (35.0-38.4)	38.5 (36.4-41.2)
**2003-2004**	35.9 (34.5-37.2)	39.1 (37.2-41.5)	36.9 (35.2-39.0)	39.0 (36.7-41.7)
**2004-2005**	36.5 (35.0-37.9)	40.1 (37.8-42.4)	37.9 (35.9-40.3)	40.1 (37.6-43.3)
**2005-2006**	36.1 (34.9-37.6)	39.8 (37.6-41.9)	37.5 (35.7-39.7)	39.8 (37.3-42.5)
**2006-2007**	36.7 (35.2-38.1)	40.5 (38.2-42.8)	38.3 (36.3-40.7)	40.7 (38.2-44.2)
**2007-2008**	36.5 (35.1-37.9)	40.0 (38.2-42.1)	37.9 (36.1-40.0)	39.9 (37.9-42.9)
**2008-2009**	36.6 (35.3-37.9)	40.2 (38.3-42.1)	38.1 (36.5-40.0)	40.1 (37.8-43.1)
**2009-2010**	36.3 (35.1-37.7)	40.0 (38.4-41.9)	38.0 (36.3-40.0)	40.3 (38.1-43.0)
**2010-2011**	36.7 (35.3-38.0)	40.1 (38.5-42.0)	38.3 (36.6-40.5)	40.6 (38.3-43.1)
**2011-2012**	36.9 (35.5-38.1)	40.4 (38.7-42.1)	38.6 (37.0-40.6)	40.9 (38.6-43.5)
**2012-2013**	36.9 (35.5-38.2)	40.3 (38.7-41.9)	38.6 (37.1-40.5)	40.9 (38.7-43.3)
**2013-2014**	36.8 (35.5-38.3)	40.5 (39.1-42.2)	38.9 (37.2-40.7)	41.3 (39.4-43.6)
**2014-2015**	37.1 (35.6-38.5)	40.8 (39.3-42.5)	39.4 (37.5-41.4)	41.5 (39.3-44.0)
**2015-2016**	36.6 (35.3-38.2)	41.4 (39.7-43.7)	40.4 (38.4-42.7)	43.1 (40.4-46.3)
**2016-2017**	36.8 (35.6-38.4)	41.0 (39.6-43.0)	40.1 (38.1-42.4)	42.5 (40.3-45.4)
**2017-2018**	36.8 (35.3-38.3)	40.9 (39.1-42.9)	40.1 (38.1-42.2)	42.8 (40.1-45.4)
**2018-2019**	36.6 (35.3-38.0)	40.9 (39.2-42.7)	40.1 (38.3-42.5)	43.0 (40.6-46.4)

For peer review only

For peer review only - http://bmjopen.bmj.com/site/about/guidelines.xhtml

Table 4: Medians (25th – 75th centiles) of ratio for users ≥90th centile over the rest of consultations per patient, by consultation type, all practices

Year	All consultations by all staff	All consultations by GPs	Face-to-face consultations by all staff	Face-to-face consultations by GPs
**2000-2001**	4.6 (4.4-4.9)	4.9 (4.6-5.4)	4.5 (4.3-4.8)	4.7 (4.5-5.1)
**2001-2002**	4.7 (4.5-5.0)	5.0 (4.7-5.5)	4.6 (4.4-4.9)	4.8 (4.5-5.2)
**2002-2003**	4.7 (4.5-5.1)	5.2 (4.8-5.6)	4.7 (4.4-5.0)	4.9 (4.6-5.3)
**2003-2004**	4.8 (4.6-5.1)	5.3 (5.0-5.8)	4.8 (4.5-5.1)	5.0 (4.7-5.5)
**2004-2005**	5.0 (4.7-5.3)	5.5 (5.1-6.1)	5.0 (4.6-5.4)	5.2 (4.8-5.8)
**2005-2006**	4.9 (4.6-5.2)	5.5 (5.1-6.0)	4.8 (4.6-5.3)	5.1 (4.8-5.7)
**2006-2007**	5.0 (4.7-5.4)	5.7 (5.2-6.3)	5.1 (4.7-5.6)	5.4 (4.9-6.0)
**2007-2008**	5.0 (4.7-5.3)	5.6 (5.1-6.0)	5.0 (4.7-5.4)	5.2 (4.8-5.7)
**2008-2009**	5.0 (4.7-5.4)	5.6 (5.2-6.0)	5.0 (4.7-5.4)	5.3 (4.9-5.7)
**2009-2010**	5.0 (4.7-5.3)	5.6 (5.2-6.0)	5.0 (4.7-5.4)	5.2 (4.9-5.7)
**2010-2011**	5.1 (4.8-5.4)	5.7 (5.3-6.1)	5.1 (4.7-5.5)	5.3 (4.9-5.8)
**2011-2012**	5.1 (4.8-5.4)	5.7 (5.3-6.1)	5.1 (4.8-5.6)	5.4 (5.0-5.8)
**2012-2013**	5.1 (4.8-5.4)	5.7 (5.4-6.1)	5.2 (4.8-5.6)	5.4 (5.0-5.8)
**2013-2014**	5.1 (4.8-5.5)	5.8 (5.4-6.2)	5.2 (4.9-5.6)	5.5 (5.1-6.0)
**2014-2015**	5.2 (4.9-5.5)	5.9 (5.5-6.3)	5.3 (5.0-5.8)	5.6 (5.2-6.1)
**2015-2016**	5.1 (4.8-5.5)	6.0 (5.6-6.5)	5.6 (5.1-6.2)	5.9 (5.4-6.6)
**2016-2017**	5.1 (4.9-5.5)	5.9 (5.5-6.4)	5.5 (5.1-6.0)	5.8 (5.3-6.4)
**2017-2018**	5.1 (4.8-5.5)	5.9 (5.5-6.3)	5.5 (5.1-6.0)	5.8 (5.4-6.5)
**2018-2019**	5.1 (4.8-5.4)	5.9 (5.5-6.3)	5.5 (5.1-6.0)	5.9 (5.4-6.6)

For peer review only

Table 5: Associations between covariates of interest and consultation volume, incidence rate ratios (and 95% CIs) from main negative binomial regression model

	All consultations – all staff	All consultations – GPs	Face-to-face consultations – all staff	Face-to-face consultations - GPs
Year	1.046 (1.045,1.047)	1.028 (1.028,1.029)	0.995 (0.994,0.997)	0.988 (0.986,0.989)
List size (per 1000 patients)	1.003 (1.002,1.004)	0.997 (0.996,0.998)	1.001 (0.998,1.003)	0.992 (0.990,0.995)
Consultation group				
Bottom 90%	Reference category			
Top 10%	5.038 (5.007,5.068)	5.603 (5.560,5.647)	4.992 (4.917,5.068)	5.253 (5.171,5.337)
IMD Deprivation quintile				
(least deprived) 1	Reference category			
2	1.005 (0.994,1.016)	1.019 (1.005,1.033)	0.993 (0.967,1.020)	1.044 (1.015,1.073)
3	1.048 (1.037,1.059)	1.049 (1.035,1.063)	1.038 (1.011,1.065)	1.076 (1.047,1.106)
4	1.019 (1.009,1.030)	1.046 (1.032,1.060)	0.981 (0.956,1.006)	1.058 (1.030,1.087)
(most deprived) 5	1.025 (1.015,1.036)	0.993 (0.980,1.006)	0.997 (0.972,1.023)	1.007 (0.981,1.035)
Region				
North East	Reference category			
North West	0.926 (0.899,0.953)	0.995 (0.958,1.034)	1.033 (0.961,1.111)	0.985 (0.913,1.063)
Yorkshire & Humber	0.952 (0.920,0.985)	1.028 (0.984,1.075)	1.023 (0.940,1.113)	1.003 (0.918,1.096)
East Midlands	0.898 (0.867,0.930)	0.912 (0.872,0.955)	0.963 (0.883,1.050)	0.915 (0.835,1.002)
West Midlands	0.840 (0.815,0.865)	1.034 (0.994,1.074)	1.074 (0.998,1.157)	1.065 (0.986,1.150)
East of England	0.853 (0.827,0.880)	0.946 (0.909,0.985)	1.012 (0.937,1.092)	0.960 (0.886,1.040)
South West	0.954 (0.926,0.983)	1.085 (1.043,1.128)	1.256 (1.166,1.353)	1.080 (0.999,1.168)
South Central	0.844 (0.819,0.870)	0.952 (0.916,0.990)	1.100 (1.021,1.186)	1.052 (0.972,1.138)
London	0.738 (0.716,0.760)	0.922 (0.887,0.958)	0.895 (0.832,0.964)	0.918 (0.850,0.992)
South East Coast	0.830 (0.805,0.855)	0.928 (0.893,0.964)	0.987 (0.918,1.063)	1.000 (0.926,1.080)
Northern Ireland	1.150 (1.115,1.187)	1.446 (1.389,1.506)	1.087 (1.006,1.174)	1.128 (1.040,1.223)
Scotland	0.785 (0.763,0.808)	1.042 (1.004,1.081)	1.249 (1.163,1.340)	1.207 (1.121,1.300)
Wales	0.883 (0.858,0.909)	0.969 (0.934,1.006)	1.045 (0.973,1.123)	1.007 (0.934,1.086)

For peer review only - http://bmjopen.bmj.com/site/about/guidelines.xhtml

Figures

Figure 1: Percentage of consultations by people consulting at the 90th centile or above within a financial year, over time

Figure 2: Ratio of consultations for people ≥90th centile vs those below within a financial year, over time

Figure 3: Consultation count estimates by group over time (Predicted incidence rates from the negative binomial regression model with a time-group interaction term)*
*Interpreted as the estimated average number of consultations for a patient within each group

For peer review only

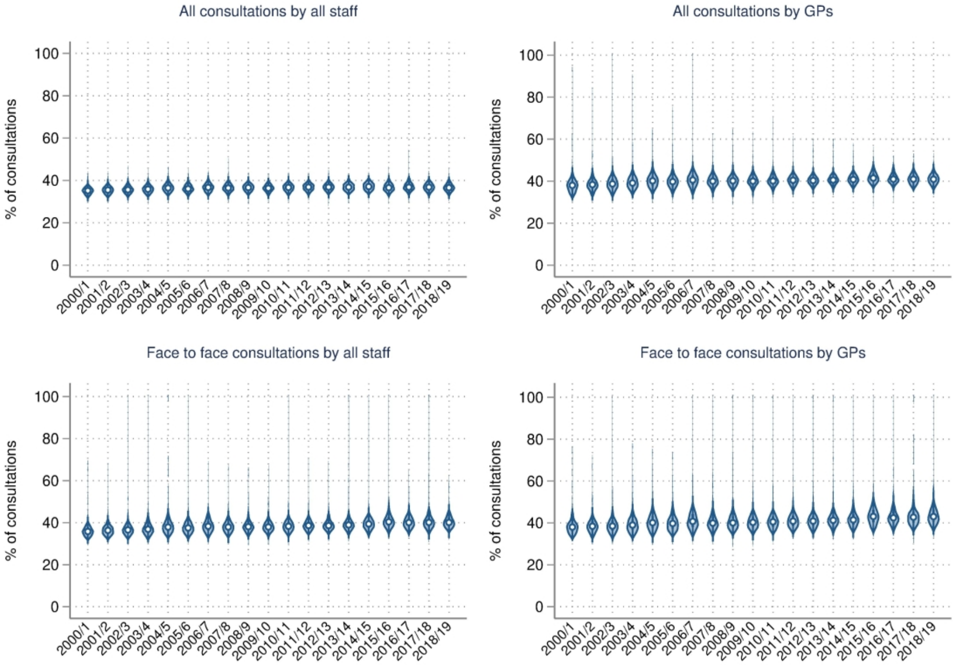

Figure 1: Percentage of consultations by people consulting at the 90th centile or above within a financial year, over time

177x127mm (600 x 600 DPI)

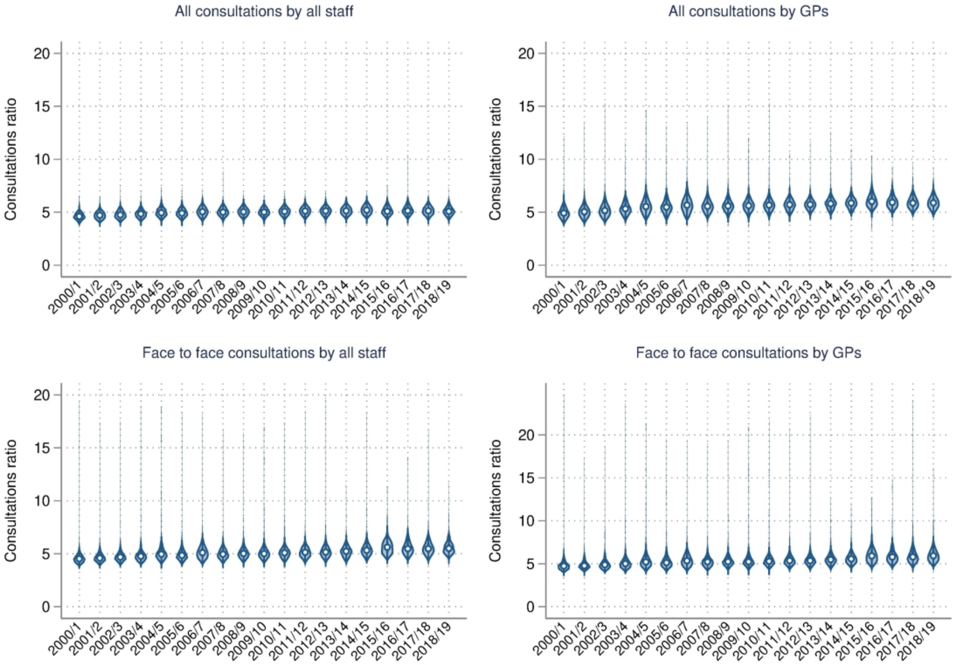

Figure 2: Ratio of consultations for people ≥90th centile vs those below within a financial year, over time

177x127mm (600 x 600 DPI)

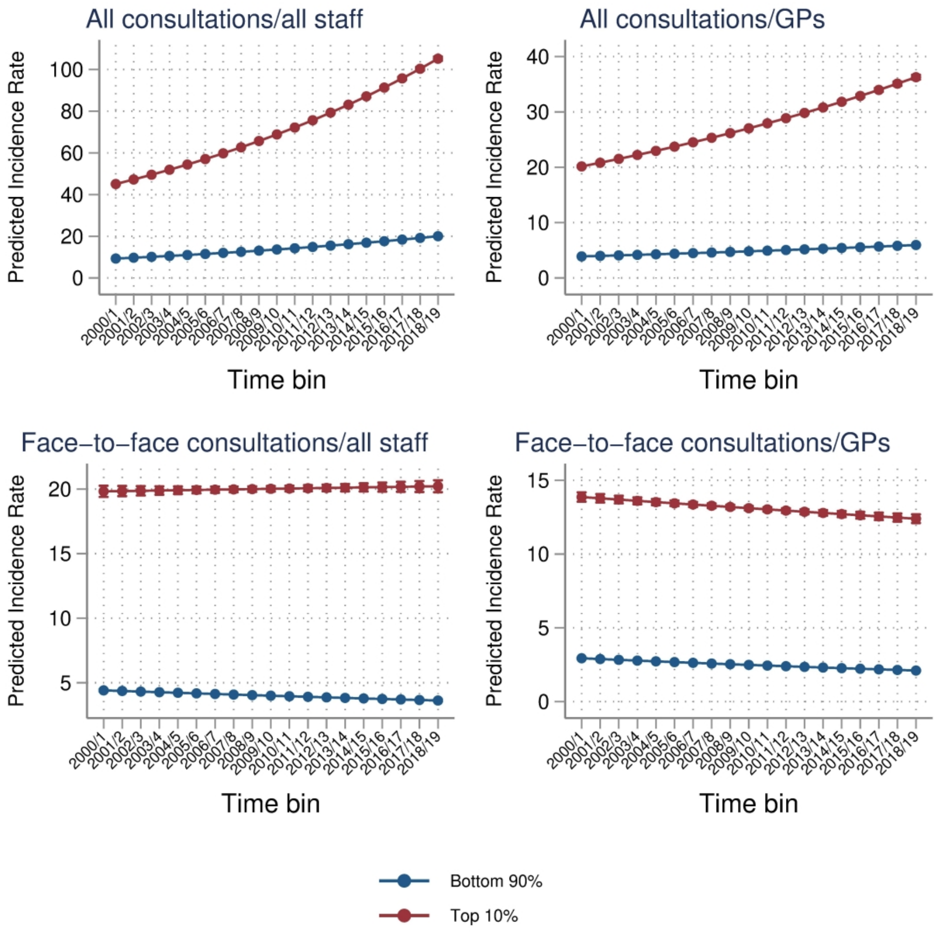

Figure 3: Consultation count estimates by group over time (Predicted incidence rates from the negative binomial regression model with a time-group interaction term)

203x203mm (600 x 600 DPI)

Supplementary file to "Consultation patterns and frequent attenders in UK primary care from 2000 to 2019: a retrospective cohort analysis of consultation events across 845 general practices"

Defining the consultation groups

GPs defined as: 1,2,3,4,5,6,7,8,9,10,47,50 in Table 1 (in red font).

Table 1: Staff Role

Code	Description
0	Data Not Entered
1	Senior Partner
2	Partner
3	Assistant
4	Associate
5	Non-commercial local rota of less than 10 GPs
6	Commercial Deputising service
7	Locum
8	GP Registrar
9	Consultant
10	Sole Practitioner
11	Practice Nurse
12	Health Visitor
13	Community Nurse
14	Midwife
15	Community Psychiatric Nurse
16	Social Worker
17	Pharmacist
18	Dispenser
19	Non-qualified Dispenser
20	Practice Manager
21	Fund Manager
22	Business Manager
23	Administrator
24	Secretary
25	Receptionist
26	Physiotherapist
27	Chiropodist
28	Dentist
29	Dietician
30	Counsellor
31	Osteopath
32	Maintenance staff
33	Other Health Care Professional
34	Hospital Nurse
35	Community Medical Officer
36	School Nurse

For peer review only - http://bmjopen.bmj.com/site/about/guidelines.xhtml

Code	Description
37	Health Education Officer
38	Contact Tracing Nurse
39	Stomatherapist
40	Computer Manager
41	Interpreter/Link Worker
42	Chiropractor
43	Acupuncturist
44	Homeopath
45	Mental Handicap Nurse
46	Carer
47	Salaried Partner
48	Occupational Therapist
49	Speech Therapist
50	GP Retainer
51	Phlebotomist
52	Other Medical & Dental
53	Other Students
54	Other Nursing & Midwifery
55	Other Allied Health Professionals
56	Other Professional Scientific & Technical
57	Other Healthcare Scientists
58	Other Additional Clinical Services
59	Other Admin & Clerical
60	Clinical Practitioner Access Role
61	Nurse Access Role
62	Nurse Manager Access Role
63	Health Professional Access Role
64	Healthcare Student Access Role
65	Biomedical Scientist Access Role
66	Clinical Coder Access Role
67	Optometrist
68	Radiographer

Face-to-face consultations defined as 1,2,3,4,6,7,8,9,11,18,24,27,28,30,31,32,33,34,36,37,38,50,61 in Table 2 (in red font).

Table 2: Consultation type

Code	Description
0	Data Not Entered
1	Clinic
2	Night visit, Deputising service
3	Follow-up/routine visit
4	Night visit, Local rota
5	Mail from patient
6	Night visit , practice
7	Out of hours, Practice
8	Out of hours, Non Practice

For peer review only - http://bmjopen.bmj.com/site/about/guidelines.xhtml

Code	Description
9	Surgery consultation
10	Telephone call from a patient
11	Acute visit
12	Discharge details
13	Letter from Outpatients
14	Repeat Issue
15	Other
16	Results recording
17	Mail to patient
18	Emergency Consultation
19	Administration
20	Casualty Attendance
21	Telephone call to a patient
22	Third Party Consultation
23	Hospital Admission
24	Children's Home Visit
25	Day Case Report
26	GOS18 Report
27	Home Visit
28	Hotel Visit
29	NHS Direct Report
30	Nursing Home Visit
31	Residential Home Visit
32	Twilight Visit
33	Triage
34	Walk-in Centre
35	Co-op Telephone advice
36	Co-op Surgery Consultation
37	Co-op Home Visit
38	Minor Injury Service
39	Medicine Management
40	Community Clinic
41	Community Nursing Note
42	Community Nursing Report
43	Data Transferred from other system
44	Health Authority Entry
45	Health Visitor Note
46	Health Visitor Report
47	Hospital Inpatient Report
48	Initial Post Discharge Review
49	Laboratory Request
50	Night Visit
51	Radiology Request
52	Radiology Result
53	Referral Letter
54	Social Services Report
55	Telephone Consultation

For peer review only

Code	Description
56	Template Entry
57	GP to GP communication transaction
58	Non-consultation medication data
59	Non-consultation data
60	ePharmacy message
61	Extended Hours

For peer review only

Median (50th centile)

Table 3 Medians (25th – 75th centiles) of median number of consultations by consultation type, all practices

Year	All consultations by all staff	All consultations by GPs	Face-to-face consultations by all staff	Face-to-face consultations by GPs
**2000-2001**	7 (6-8)	3 (3-4)	4 (3-4)	2 (2-3)
**2001-2002**	8 (7-9)	3 (3-4)	4 (3-4)	2 (2-3)
**2002-2003**	9 (7-10)	3 (3-4)	3 (3-4)	2 (2-3)
**2003-2004**	9 (7-11)	3 (3-4)	3 (3-4)	2 (2-3)
**2004-2005**	9 (7-11)	3 (3-4)	3 (3-4)	2 (2-3)
**2005-2006**	10 (8-13)	4 (3-4)	4 (3-4)	2 (2-3)
**2006-2007**	10 (8-13)	4 (3-4)	3 (3-4)	2 (2-3)
**2007-2008**	11 (9-13)	4 (3-5)	3 (3-4)	2 (2-3)
**2008-2009**	12 (10-14)	4 (3-5)	3 (3-4)	2 (2-3)
**2009-2010**	13 (11-15)	4 (3-5)	4 (3-4)	2 (2-3)
**2010-2011**	13 (11-15)	4 (3-5)	3 (3-4)	2 (2-3)
**2011-2012**	13 (11-15)	4 (4-5)	3 (3-4)	2 (2-3)
**2012-2013**	14 (12-16)	4 (4-5)	4 (3-4)	2 (2-3)
**2013-2014**	14 (12-17)	5 (4-5)	4 (3-4)	2 (2-3)
**2014-2015**	14 (12-17)	5 (4-6)	3 (3-4)	2 (2-3)
**2015-2016**	14 (12-17)	4 (3-5)	3 (3-4)	2 (1-2)
**2016-2017**	15 (12-17)	5 (4-6)	3 (3-4)	2 (1-2)
**2017-2018**	15 (13-18)	5 (4-6)	3 (3-4)	2 (1-2)
**2018-2019**	16 (13-18)	5 (4-6)	3 (2-4)	2 (1-2)

For peer review only - http://bmjopen.bmj.com/site/about/guidelines.xhtml

Table 4 Medians (25th – 75th centiles) of proportion of high volume (≥ 50th centile) by consultation type, all practices

Year	All consultations by all staff	All consultations by GPs	Face-to-face consultations by all staff	Face-to-face consultations by GPs
**2000-2001**	87.0 (86.2-87.8)	90.2 (88.2-92.4)	87.9 (86.0-90.1)	91.6 (87.9-94.0)
**2001-2002**	87.3 (86.4-88.1)	90.3 (88.4-92.5)	88.4 (86.8-90.4)	92.2 (88.7-94.0)
**2002-2003**	87.4 (86.6-88.2)	90.8 (89.1-93.0)	88.8 (87.2-90.5)	92.7 (89.2-94.2)
**2003-2004**	87.6 (86.9-88.4)	90.9 (89.3-92.8)	89.1 (87.5-90.8)	92.7 (89.6-94.1)
**2004-2005**	88.3 (87.5-89.1)	91.8 (89.7-93.9)	90.2 (88.4-92.6)	93.3 (90.5-94.9)
**2005-2006**	88.0 (87.2-88.8)	91.5 (89.7-93.2)	89.5 (87.8-91.5)	92.9 (90.3-94.4)
**2006-2007**	88.4 (87.5-89.3)	92.1 (90.1-94.1)	90.7 (88.5-92.7)	93.4 (91.0-95.2)
**2007-2008**	88.1 (87.2-88.9)	91.5 (89.9-93.0)	89.7 (87.9-91.7)	93.0 (90.7-94.5)
**2008-2009**	88.1 (87.3-88.8)	91.5 (90.0-93.0)	90.0 (88.3-91.9)	93.4 (91.2-94.8)
**2009-2010**	87.9 (87.2-88.7)	91.3 (90.0-93.0)	89.8 (88.2-91.9)	93.2 (90.8-94.7)
**2010-2011**	88.0 (87.3-88.8)	91.4 (90.0-93.0)	90.0 (88.3-92.0)	93.3 (91.2-94.8)
**2011-2012**	88.1 (87.4-88.8)	91.4 (90.1-92.6)	90.2 (88.6-92.0)	93.3 (91.4-95.0)
**2012-2013**	88.1 (87.4-88.9)	91.4 (90.1-92.6)	90.0 (88.5-92.0)	93.4 (91.1-94.9)
**2013-2014**	88.1 (87.3-88.8)	91.5 (90.4-92.7)	90.3 (88.8-92.0)	93.5 (91.7-95.2)
**2014-2015**	88.3 (87.4-88.9)	91.6 (90.4-93.0)	90.9 (89.3-92.5)	93.7 (91.8-95.3)
**2015-2016**	87.7 (86.8-88.7)	92.2 (90.7-94.2)	92.0 (90.1-94.1)	94.7 (92.9-100)
**2016-2017**	88.2 (87.4-88.9)	91.8 (90.6-93.3)	91.7 (89.8-93.4)	94.4 (92.3-96.5)
**2017-2018**	88.2 (87.3-88.9)	91.7 (90.4-93.1)	91.6 (89.7-93.4)	94.4 (92.4-100)
**2018-2019**	88.2 (87.3-88.9)	91.9 (90.6-92.9)	91.8 (89.9-93.9)	94.5 (92.6-100)

For peer review only

Table 5: Medians (25th – 75th centiles) of ratio for users ≥50th centile over the rest of consultations by consultation type, all practices

Year	All consultations by all staff	All consultations by GPs	Face-to-face consultations by all staff	Face-to-face consultations by GPs
2000-2001	6.1 (5.7-6.4)	7.5 (6.5-8.9)	6.0 (5.5-6.7)	7.7 (6.3-9.5)
2001-2002	6.3 (5.9-6.7)	7.7 (6.6-9.1)	6.4 (5.8-7.1)	8.2 (6.6-9.6)
2002-2003	6.5 (6.0-6.9)	8.0 (6.9-9.7)	6.5 (6.0-7.4)	8.5 (6.8-9.8)
2003-2004	6.6 (6.2-7.1)	8.4 (7.3-10.1)	6.7 (6.2-7.6)	8.7 (7.2-10.1)
2004-2005	7.0 (6.6-7.6)	9.2 (7.6-12.0)	7.5 (6.6-9.7)	9.4 (7.7-11.4)
2005-2006	6.9 (6.5-7.4)	9.0 (7.8-10.7)	7.1 (6.4-8.2)	9.2 (7.6-10.6)
2006-2007	7.2 (6.6-7.8)	9.9 (8.0-12.6)	7.8 (6.8-10)	9.9 (8.0-12.1)
2007-2008	7.0 (6.6-7.5)	9.2 (7.9-10.8)	7.2 (6.5-8.5)	9.2 (7.7-10.7)
2008-2009	7.0 (6.6-7.5)	9.2 (8.1-11.0)	7.4 (6.6-8.6)	9.7 (8.2-11.4)
2009-2010	6.9 (6.5-7.4)	9.3 (8.0-11.0)	7.4 (6.6-8.6)	9.8 (8.0-11.2)
2010-2011	7.0 (6.6-7.5)	9.3 (8.0-11.0)	7.5 (6.6-8.6)	9.9 (8.3-11.4)
2011-2012	7.1 (6.6-7.6)	9.3 (8.1-10.7)	7.5 (6.8-8.7)	10.0 (8.4-11.4)
2012-2013	7.1 (6.7-7.6)	9.3 (8.2-10.6)	7.6 (6.8-8.8)	9.8 (8.3-11.4)
2013-2014	7.1 (6.6-7.6)	9.6 (8.5-11.0)	7.7 (7.0-8.9)	10.2 (8.7-11.9)
2014-2015	7.2 (6.7-7.7)	9.7 (8.6-11.3)	8.2 (7.3-9.5)	10.5 (8.9-12.1)
2015-2016	6.9 (6.4-7.5)	10.4 (8.6-14.4)	9.5 (7.7-11.9)	11.4 (9.6-14.2)
2016-2017	7.2 (6.6-7.7)	9.8 (8.7-12.1)	8.7 (7.6-10.5)	11.0 (9.3-13.0)
2017-2018	7.1 (6.6-7.7)	9.9 (8.7-11.8)	8.6 (7.6-10.8)	11.1 (9.2-12.9)
2018-2019	7.2 (6.6-7.8)	9.9 (8.8-11.8)	8.9 (7.8-11.2)	11.2 (9.6-13.3)

For peer review only

75th centile

Table 6 Medians (25th – 75th centiles) of 75th centile number of consultations by consultation type, all practices

Year	All consultations by all staff	All consultations by GPs	Face-to-face consultations by all staff	Face-to-face consultations by GPs
**2000-2001**	16 (14-18)	7 (6-10)	7 (6-9)	5 (4-6)
**2001-2002**	17.5 (15-20)	7 (6-9)	7 (6-9)	5 (4-6)
**2002-2003**	19 (16-22)	7 (6-9)	7 (6-9)	5 (4-6)
**2003-2004**	21 (17-24)	8 (6-10)	7 (6-9)	5 (4-6)
**2004-2005**	22 (18-26)	8 (7-10)	7 (6-9)	5 (4-6)
**2005-2006**	24 (19-28)	9 (7-11)	7 (6-10)	5 (4-6)
**2006-2007**	24 (20-29)	9 (7-11)	7 (6-9)	4 (4-6)
**2007-2008**	26 (21-30)	9 (7-11)	7 (6-9)	5 (4-6)
**2008-2009**	27 (23-31.5)	9 (8-11)	7 (6-9)	5 (4-6)
**2009-2010**	28 (24-33)	10 (8-12)	7 (6-9)	5 (4-6)
**2010-2011**	28 (24-33)	10 (8-12)	7 (6-9)	5 (4-6)
**2011-2012**	29 (25-34)	10 (9-12)	7 (6-9)	5 (4-6)
**2012-2013**	30 (26-35)	10 (9-12)	7 (6-9)	5 (4-6)
**2013-2014**	32 (27-37)	11 (9-13)	7 (6-9)	5 (4-6)
**2014-2015**	32 (28-37)	11 (10-13)	7 (6-9)	5 (4-6)
**2015-2016**	32 (28-37)	11 (9-13)	7 (6-8)	5 (4-5)
**2016-2017**	32 (28-38)	11 (9-13)	7 (6-8)	5 (4-6)
**2017-2018**	33 (29-38)	11 (9-13)	7 (6-8)	4 (3-5)
**2018-2019**	35 (30-40)	11 (10-13)	7 (6-8)	4 (3-5)

For peer review only

Table 7 Medians (25th – 75th centiles) of proportion of high volume (≥ 75th centile) by consultation type, all practices

Year	All consultations by all staff	All consultations by GPs	Face-to-face consultations by all staff	Face-to-face consultations by GPs
**2000-2001**	62.4 (61.2-63.9)	65.8 (63.5-69.1)	63.7 (61.6-66.0)	66.4 (63.8-69.9)
**2001-2002**	62.9 (61.6-64.3)	66.5 (64.0-69.1)	63.7 (61.8-66.4)	66.8 (64.3-70.4)
**2002-2003**	63.1 (61.9-64.6)	67.4 (64.7-69.8)	64.4 (62.3-66.7)	67.9 (64.3-71.3)
**2003-2004**	63.5 (62.2-64.8)	67.7 (65.4-70.2)	64.9 (62.7-67.3)	68.2 (65.0-71.8)
**2004-2005**	64.3 (63.0-65.7)	68.9 (66.1-71.7)	66.2 (63.9-69.0)	69.5 (66.1-73.4)
**2005-2006**	63.8 (62.6-65.3)	68.2 (65.9-70.8)	65.4 (63.2-67.7)	68.9 (65.7-72.2)
**2006-2007**	64.6 (63.0-66.0)	69.3 (66.3-71.9)	66.4 (64.0-69.4)	70.3 (66.7-73.9)
**2007-2008**	64.1 (62.7-65.4)	68.4 (66.4-70.7)	65.5 (63.2-68.0)	69.2 (66.0-72.8)
**2008-2009**	64.2 (62.7-65.5)	68.2 (66.5-70.7)	65.8 (63.9-68.6)	69.5 (66.4-72.7)
**2009-2010**	63.8 (62.6-65.2)	68.4 (66.2-70.5)	65.8 (63.7-68.0)	69.5 (66.6-72.4)
**2010-2011**	64.0 (62.7-65.5)	68.3 (66.4-70.4)	66.3 (64.2-68.5)	69.5 (66.8-72.4)
**2011-2012**	64.3 (62.9-65.4)	68.4 (66.5-70.2)	66.2 (64.1-68.8)	69.8 (67.0-73.0)
**2012-2013**	64.3 (62.9-65.6)	68.4 (66.8-70.2)	66.5 (64.6-68.8)	69.6 (67.0-73.1)
**2013-2014**	64.2 (62.9-65.7)	68.7 (66.8-70.5)	66.7 (64.6-69.0)	70.1 (67.4-73.4)
**2014-2015**	64.5 (62.9-65.9)	69.0 (67.3-70.8)	67.3 (65.3-69.4)	70.6 (67.9-73.8)
**2015-2016**	63.7 (62.4-65.5)	69.6 (67.4-72.5)	68.8 (66.4-71.7)	72.3 (69.1-76.8)
**2016-2017**	64.3 (62.8-65.8)	69.2 (67.3-71.2)	68.2 (66.0-70.7)	71.5 (68.3-75.6)
**2017-2018**	64.1 (62.7-65.8)	69.0 (67.1-71.3)	68.4 (65.5-71.4)	72.1 (69.0-76.1)
**2018-2019**	64.2 (62.6-65.5)	69.0 (67.3-71.3)	68.6 (66.2-71.2)	72.6 (69.3-77.2)

For peer review only

Table 8: Medians (25th – 75th centiles) of ratio for users ≥75th centile over the rest of consultations by consultation type, all practices

Year	All consultations by all staff	All consultations by GPs	Face-to-face consultations by all staff	Face-to-face consultations by GPs
2000-2001	4.7 (4.5-5.0)	5.1 (4.8-5.7)	4.6 (4.4-4.9)	5.0 (4.6-5.5)
2001-2002	4.8 (4.6-5.1)	5.3 (4.9-5.8)	4.7 (4.4-5.0)	5.1 (4.7-5.7)
2002-2003	4.9 (4.6-5.2)	5.4 (5.0-6.1)	4.8 (4.5-5.2)	5.1 (4.8-5.8)
2003-2004	5.0 (4.7-5.3)	5.7 (5.2-6.3)	4.9 (4.6-5.3)	5.3 (4.9-6.0)
2004-2005	5.2 (4.9-5.5)	6.0 (5.4-6.7)	5.2 (4.8-5.8)	5.7 (5.2-6.6)
2005-2006	5.1 (4.8-5.4)	5.8 (5.4-6.5)	5.0 (4.7-5.5)	5.5 (5.0-6.3)
2006-2007	5.3 (4.9-5.6)	6.2 (5.5-7.0)	5.3 (4.9-6.0)	5.9 (5.2-6.8)
2007-2008	5.2 (4.9-5.5)	5.9 (5.5-6.5)	5.1 (4.7-5.5)	5.6 (5.1-6.2)
2008-2009	5.2 (4.9-5.5)	5.9 (5.5-6.6)	5.1 (4.8-5.6)	5.6 (5.2-6.3)
2009-2010	5.1 (4.9-5.4)	6.0 (5.5-6.5)	5.1 (4.8-5.6)	5.6 (5.2-6.3)
2010-2011	5.2 (4.9-5.5)	6.0 (5.5-6.5)	5.2 (4.8-5.6)	5.6 (5.2-6.2)
2011-2012	5.2 (4.9-5.5)	6.0 (5.6-6.5)	5.2 (4.9-5.7)	5.7 (5.3-6.3)
2012-2013	5.2 (4.9-5.6)	6.0 (5.6-6.5)	5.3 (4.9-5.7)	5.8 (5.3-6.4)
2013-2014	5.2 (5.0-5.6)	6.1 (5.7-6.6)	5.3 (5.0-5.8)	5.9 (5.4-6.5)
2014-2015	5.3 (5.0-5.6)	6.2 (5.8-6.7)	5.4 (5.1-6.0)	6.0 (5.5-6.7)
2015-2016	5.2 (4.8-5.6)	6.4 (5.9-7.3)	5.9 (5.3-6.6)	6.6 (5.8-7.7)
2016-2017	5.3 (4.9-5.6)	6.3 (5.8-6.9)	5.7 (5.2-6.3)	6.3 (5.7-7.3)
2017-2018	5.2 (4.9-5.6)	6.3 (5.7-6.8)	5.7 (5.2-6.3)	6.4 (5.8-7.4)
2018-2019	5.2 (4.9-5.6)	6.3 (5.8-6.9)	5.7 (5.3-6.4)	6.5 (5.8-7.7)

For peer review only

Twelve or more consultations

Table 9 Medians (25th – 75th centiles) of proportion of patients with 12 or more consultations by consultation type, all practices

Year	All consultations by all staff	All consultations by GPs	Face-to-face consultations by all staff	Face-to-face consultations by GPs
**2000-2001**	36.0 (30.5-40.5)	12.6 (7.5-20.1)	10.6 (7.9-15.6)	4.9 (3.4-9.3)
**2001-2002**	38.9 (32.9-42.8)	12.5 (8.1-17.9)	11.2 (7.9-16.7)	4.6 (3.0-8.4)
**2002-2003**	40.9 (34.7-45.9)	12.7 (7.7-18.2)	10.7 (7.5-16.4)	4.3 (2.8-7.7)
**2003-2004**	43.3 (37.2-48.4)	14.4 (10.2-19.5)	10.8 (7.5-18.1)	4.2 (2.6-7.5)
**2004-2005**	44.3 (37.8-49.5)	15.7 (11.6-20.7)	11.2 (7.4-18.4)	4.1 (2.5-7.9)
**2005-2006**	47.2 (40.7-52.5)	17.0 (13.3-22.7)	11.9 (8.1-20.1)	4.3 (2.7-8.0)
**2006-2007**	47.3 (41.0-52.4)	17.5 (13.2-22.8)	11.6 (7.7-19.5)	4.2 (2.6-8.2)
**2007-2008**	49.5 (44.2-53.9)	18.0 (13.0-23.3)	11.8 (8.2-17.9)	4.2 (2.8-7.2)
**2008-2009**	51.1 (46.2-55.2)	19.0 (14.3-23.7)	11.7 (8.4-16.9)	4.4 (2.9-6.6)
**2009-2010**	52.8 (48.2-56.6)	20.0 (15.4-25.4)	12.2 (9.0-17.0)	4.7 (2.9-6.8)
**2010-2011**	52.7 (48.3-56.7)	20.7 (16.4-25.3)	12.1 (8.6-16.3)	4.7 (2.9-7.0)
**2011-2012**	53.8 (49.4-57.5)	21.8 (17.3-26.2)	12.2 (8.7-16.1)	4.7 (3.0-7.0)
**2012-2013**	54.5 (50.4-58.4)	22.3 (18.1-27.4)	12.4 (9.1-16.4)	4.6 (3.0-7.4)
**2013-2014**	55.6 (51.2-59.7)	23.8 (19.4-28.6)	12.5 (8.9-16.4)	4.6 (2.9-7.4)
**2014-2015**	56.0 (51.8-60.1)	24.1 (19.8-29.2)	12.3 (8.8-16.3)	4.6 (2.9-7.3)
**2015-2016**	56.2 (52.1-60.5)	23.0 (18.7-28.0)	11.7 (8.3-15.5)	4.5 (2.4-7.2)
**2016-2017**	56.4 (52.1-60.4)	24.0 (18.9-29.2)	11.8 (8.2-16.1)	4.5 (2.5-7.7)
**2017-2018**	56.9 (52.9-60.9)	24.0 (19.6-28.9)	11.4 (7.4-15.8)	4.2 (2.2-7.3)
**2018-2019**	58.4 (54.1-61.8)	24.7 (19.9-29.2)	11.3 (7.3-15.4)	4.3 (2.1-7.1)

For peer review only

Table 10 Medians (25th – 75th centiles) of proportion of high volume (12 or more consultations) by consultation type, all practices

Year	All consultations by all staff	All consultations by GPs	Face-to-face consultations by all staff	Face-to-face consultations by GPs
**2000-2001**	73.9 (68.3-78.5)	43.1 (30.6-56.4)	35.8 (28.1-47.0)	20.7 (15.7-34.1)
**2001-2002**	77.6 (71.5-81.3)	43.2 (31.7-53.7)	36.8 (28.6-48.1)	20.2 (15.1-31.7)
**2002-2003**	79.8 (74.1-83.8)	43.6 (31.1-54.8)	36.1 (28.3-49.3)	19.4 (14.0-29.8)
**2003-2004**	82.3 (76.5-85.9)	48.3 (37.5-57.2)	37.3 (28.4-52.7)	19.4 (13.8-31.0)
**2004-2005**	83.8 (78.2-87.0)	51.7 (42.4-60.2)	39.3 (29.7-55.8)	19.7 (13.6-33.3)
**2005-2006**	85.6 (80.5-88.8)	54.1 (45.7-62.5)	40.4 (30.7-57.0)	20.1 (14.4-33.4)
**2006-2007**	86.1 (81.5-89.1)	55.9 (46.9-64.2)	41.1 (31.8-56.0)	20.4 (14.8-33.2)
**2007-2008**	87.2 (83.3-89.8)	55.7 (46.2-64.0)	40.2 (31.4-52.6)	20.4 (14.8-30.6)
**2008-2009**	88.3 (84.9-90.6)	57.5 (48.5-64.5)	40.1 (32.0-51.0)	21.2 (15.1-28.9)
**2009-2010**	88.9 (86.2-91.1)	59.3 (50.6-66.6)	41.0 (33.2-51.0)	22.0 (15.5-29.5)
**2010-2011**	89.1 (86.4-91.3)	59.9 (52.3-66.7)	41.0 (32.7-50.1)	21.9 (15.7-30.1)
**2011-2012**	89.8 (87.3-91.7)	61.6 (54.1-68.4)	41.7 (33.0-49.5)	21.9 (16.0-30.0)
**2012-2013**	90.2 (87.8-92.1)	62.8 (56.1-70.3)	42.4 (33.4-50.0)	22.3 (16.1-31.3)
**2013-2014**	90.7 (88.3-92.6)	65.1 (58.4-71.4)	42.5 (33.8-50.5)	22.1 (15.8-32.1)
**2014-2015**	90.9 (88.7-92.8)	66.2 (59.6-72.0)	43.2 (34.0-50.5)	22.9 (16.1-32.4)
**2015-2016**	90.6 (88.3-92.5)	65.7 (58.4-71.6)	43.0 (33.7-50.8)	23.1 (15.2-32.4)
**2016-2017**	91.2 (88.9-93.0)	66.1 (58.7-72.7)	42.9 (33.5-51.5)	22.7 (14.5-34.0)
**2017-2018**	91.3 (89.3-93.2)	65.8 (59.2-72.2)	41.7 (32.0-51.5)	22.9 (14.0-32.5)
**2018-2019**	92.0 (90.2-93.6)	67.0 (59.9-73.0)	41.6 (31.5-50.5)	22.2 (13.4-32.0)

For peer review only

STROBE Statement—Checklist of items that should be included in reports of ***cohort studies***

	Item No	Recommendation	
**Title and abstract**	1	(*a*) Indicate the study's design with a commonly used term in the title or the abstract	p1
		(*b*) Provide in the abstract an informative and balanced summary of what was done and what was found	p3
**Introduction**			
Background/rationale	2	Explain the scientific background and rationale for the investigation being reported	p4
Objectives	3	State specific objectives, including any prespecified hypotheses	p5
**Methods**			
Study design	4	Present key elements of study design early in the paper	p5
Setting	5	Describe the setting, locations, and relevant dates, including periods of recruitment, exposure, follow-up, and data collection	p5
Participants	6	(*a*) Give the eligibility criteria, and the sources and methods of selection of participants. Describe methods of follow-up	p5
		(*b*) For matched studies, give matching criteria and number of exposed and unexposed	NA
Variables	7	Clearly define all outcomes, exposures, predictors, potential confounders, and effect modifiers. Give diagnostic criteria, if applicable	p5-6
Data sources/ measurement	8*	For each variable of interest, give sources of data and details of methods of assessment (measurement). Describe comparability of assessment methods if there is more than one group	p5-6
Bias	9	Describe any efforts to address potential sources of bias	p6
Study size	10	Explain how the study size was arrived at	p5
Quantitative variables	11	Explain how quantitative variables were handled in the analyses. If applicable, describe which groupings were chosen and why	p5-6
Statistical methods	12	(*a*) Describe all statistical methods, including those used to control for confounding	p6
		(*b*) Describe any methods used to examine subgroups and interactions	p6
		(*c*) Explain how missing data were addressed	NA
		(*d*) If applicable, explain how loss to follow-up was addressed	NA
		(*e*) Describe any sensitivity analyses	p6
**Results**			
Participants	13*	(a) Report numbers of individuals at each stage of study—eg numbers potentially eligible, examined for eligibility, confirmed eligible, included in the study, completing follow-up, and analysed	p7
		(b) Give reasons for non-participation at each stage	NA
		(c) Consider use of a flow diagram	NA
Descriptive data	14*	(a) Give characteristics of study participants (eg demographic, clinical, social) and information on exposures and potential confounders	NA
		(b) Indicate number of participants with missing data for each variable of interest	NA
		(c) Summarise follow-up time (eg, average and total amount)	NA
Outcome data	15*	Report numbers of outcome events or summary measures over time	p7
Main results	16	(*a*) Give unadjusted estimates and, if applicable, confounder-adjusted estimates and their precision (eg, 95% confidence interval). Make clear which confounders were adjusted for and why they were included	p7
		(*b*) Report category boundaries when continuous variables were categorized	NA
		(*c*) If relevant, consider translating estimates of relative risk into absolute risk for a meaningful time period	p7

For peer review only - http://bmjopen.bmj.com/site/about/guidelines.xhtml

Other analyses	17	Report other analyses done—eg analyses of subgroups and interactions, and sensitivity analyses	p8
**Discussion**			
Key results	18	Summarise key results with reference to study objectives	p8
Limitations	19	Discuss limitations of the study, taking into account sources of potential bias or imprecision. Discuss both direction and magnitude of any potential bias	p9-10
Interpretation	20	Give a cautious overall interpretation of results considering objectives, limitations, multiplicity of analyses, results from similar studies, and other relevant evidence	p10
Generalisability	21	Discuss the generalisability (external validity) of the study results	p10
**Other information**			
Funding	22	Give the source of funding and the role of the funders for the present study and, if applicable, for the original study on which the present article is based	p11

*Give information separately for exposed and unexposed groups.

Note: An Explanation and Elaboration article discusses each checklist item and gives methodological background and published examples of transparent reporting. The STROBE checklist is best used in conjunction with this article (freely available on the Web sites of PLoS Medicine at http://www.plosmedicine.org/, Annals of Internal Medicine at http://www.annals.org/, and Epidemiology at http://www.epidem.com/). Information on the STROBE Initiative is available at http://www.strobe-statement.org.

For peer review only

For peer review only - http://bmjopen.bmj.com/site/about/guidelines.xhtml
